# Refined Universality for Critical KCM: Upper Bounds

**DOI:** 10.1007/s00220-023-04874-8

**Published:** 2024-01-25

**Authors:** Ivailo Hartarsky

**Affiliations:** Technische Universität Wien, Institut für Stochastik und Wirtschaftsmathematik, Wiedner Hauptstraße 8-10, 1040 Vienna, Austria

## Abstract

We study a general class of interacting particle systems called kinetically constrained models (KCM) in two dimensions. They are tightly linked to the monotone cellular automata called bootstrap percolation. Among the three classes of such models (Bollobás et al. in Combin Probab Comput 24(4):687–722, 2015), the critical ones are the most studied. Together with the companion paper by Marêché and the author (Hartarsky and Marêché in Combin Probab Comput 31(5):879–906, 2022), our work determines the logarithm of the infection time up to a constant factor for all critical KCM. This was previously known only up to logarithmic corrections (Hartarsky et al. in Probab Theory Relat Fields 178(1):289–326, 2020, Ann Probab 49(5):2141–2174, 2021, Martinelli et al. in Commun Math Phys 369(2):761–809, 2019). We establish that on this level of precision critical KCM have to be classified into seven categories. This refines the two classes present in bootstrap percolation (Bollobás et al. in Proc Lond Math Soc (3) 126(2):620–703, 2023) and the two in previous rougher results (Hartarsky et al. in Probab Theory Relat Fields 178(1):289–326, 2020, Ann Probab 49(5):2141–2174, 2021, Martinelli et al. in Commun Math Phys 369(2):761–809, 2019). In the present work we establish the upper bounds for the novel five categories and thus complete the universality program for equilibrium critical KCM. Our main innovations are the identification of the dominant relaxation mechanisms and a more sophisticated and robust version of techniques recently developed for the study of the Fredrickson-Andersen 2-spin facilitated model (Hartarsky et al. in Probab Theory Relat Fields 185(3):993–1037, 2023).

## Introduction

Kinetically constrained models (KCM) are interacting particle systems. They have challenging features including non-ergodicity, non-attractiveness, hard constraints, cooperative dynamics and dramatically diverging time scales. This prevents the use of conventional mathematical tools in the field.

KCM originated in physics in the 1980 s [12, 13] as toy models for the liquid-glass transition, which is still a hot and largely open topic for physicists [3]. The idea behind them is that one can induce glassy behaviour without the intervention of static interactions, disordered or not, but rather with simple kinetic constraints. The latter translate the phenomenological observation that at high density particles in a super-cooled liquid become trapped by their neighbours and require a scarce bit of empty space in order to move at all. We direct the reader interested in the motivations of these models and their position in the landscape of glass transition theories to [3, 14, 35].

Bootstrap percolation is the natural monotone deterministic counterpart of KCM (see [34] for an overview). Nevertheless, the two subjects arose for different reasons and remained fairly independent until the late 2000 s. That is when the very first rigorous results for KCM came to be [9], albeit much less satisfactory than their bootstrap percolation predecessors. The understanding of these two closely related fields did not truly unify until the recent series of works [20–22, 24, 30–32] elucidating the common points, as well as the serious additional difficulties in the non-monotone stochastic setting. It is the goal of this series that is accomplished by the present paper.

### Models

Let us introduce the class of $\mathcal U$-KCM introduced in [9]. In $d\geqslant 1$ dimensions an *update family* is a nonempty finite collection of finite nonempty subsets of ${\mathbb Z} ^d\setminus \{0\}$ called *update rules*. The $\mathcal U$-KCM is a continuous time Markov chain with state space ${\Omega }=\{0,1\}^{{\mathbb Z} ^d}$. Given a configuration ${\eta }\in {\Omega }$, we write ${\eta }_x$ for the state of $x\in {\mathbb Z} ^d$ in ${\eta }$ and say that *x* *is infected* (in ${\eta }$) if ${\eta }_x=0$. We write ${\eta }_A$ for the restriction of ${\eta }$ to $A\subset {\mathbb Z} ^d$ and $\textbf{0}_A$ for the completely infected configuration with *A* omitted when it is clear from the context. We say that *the constraint at *$x\in {\mathbb Z} ^d$ *is satisfied* if there exists an update rule $U\in \mathcal U$ such that $x+U=\{x+y:y\in U\}$ is fully infected. We denote the corresponding indicator by

1 $$\begin{aligned} c_x({\eta })={\mathbb {1}} _{\exists U\in \mathcal U,{\eta }_{x+U}=\textbf{0}}. \end{aligned}$$

The final parameter of the model is its *equilibrium density of infections* $q\in [0,1]$. We denote by ${\mu }$ the product measure such that ${\mu }({\eta }_x=0)=q$ for all $x\in {\mathbb Z} ^d$ and by ${\text {Var}}$ the corresponding variance. Given a finite set $A\subset {\mathbb Z} ^d$ and real function $f:{\Omega }\rightarrow {\mathbb R}$, we write ${\mu }_A(f)$ for the average ${\mu }(f({\eta })|{\eta }_{{\mathbb Z} ^d\setminus A})$ of *f* over the variables in *A*. We write ${\text {Var}}_A(f)$ for the corresponding conditional variance, which is thus also a function from ${\Omega }_{{\mathbb Z} ^d\setminus A}$ to ${\mathbb R}$, where ${\Omega }_B=\{0,1\}^B$ for $B\subset {\mathbb Z} ^d$.

With this notation the $\mathcal U$-KCM can be formally defined via its generator

2 $$\begin{aligned} \mathcal L(f)({\eta })=\sum _{x\in {\mathbb Z} ^d}c_x({\eta })\cdot \left( {\mu }_x(f)-f\right) ({\eta }) \end{aligned}$$

and its Dirichlet form reads

$$\begin{aligned} \mathcal D(f)=\sum _{x\in {\mathbb Z} ^d}{\mu }\left( c_x\cdot {\text {Var}}_x(f)\right) , \end{aligned}$$

where ${\mu }_x$ and ${\text {Var}}_x$ are shorthand for ${\mu }_{\{x\}}$ and ${\text {Var}}_{\{x\}}$. Alternatively, the process can be defined via a graphical representation as follows (see [28] for background). Each site $x\in {\mathbb Z} ^d$ is endowed with a standard Poisson process called *clock*. Whenever the clock at *x* rings we assess whether its constraint is satisfied by the current configuration. If it is, we update ${\eta }_x$ to an independent Bernoulli variable with parameter $1-q$ and call this a *legal update*. If the constraint is not satisfied, the update is *illegal*, so we discard it without modifying the configuration. It is then clear that ${\mu }$ is a reversible measure for the process (there are others, e.g. the Dirac measure on the fully non-infected configuration $\textbf{1}$).

Our regime of interest is $q\rightarrow 0$, corresponding to the low temperature limit relevant for glasses. A quantitative observable, measuring the speed of the dynamics, is the infection time of 0

$$\begin{aligned} {\tau }_0=\textrm{inf}\left\{ t\geqslant 0:{\eta }_0(t)=0\right\} , \end{aligned}$$

where $({\eta }(t))_{t\geqslant 0}$ denotes the $\mathcal U$-KCM process. More specifically, we are interested in its expectation for the stationary process ${\mathbb E} _{{\mu }}[{\tau }_0]$, namely the process with random initial condition distributed according to ${\mu }$. This quantifies the equilibrium properties of the system and is closely related e.g. to the more analytic quantity called relaxation time (i.e. inverse of the spectral gap of the generator) that the reader may be familiar with.

$\mathcal U$-bootstrap percolation is essentially the $q=1$ case of $\mathcal U$-KCM started out of equilibrium, from a product measure with $q_0\rightarrow 0$ density of infections. More conventionally, it is defined as a synchronous cellular automaton, which updates all sites of ${\mathbb Z} ^d$ simultaneously at each discrete time step, by infecting sites whose constraint is satisfied and never removing infections. As the process is monotone, it may alternatively be viewed as a growing subset of the grid generated by its initial condition. We denote by $[A]_\mathcal U$ the set of sites eventually infected by the $\mathcal U$-bootstrap percolation process with initial condition $A\subset {\mathbb Z} ^d$, that is, the sites which can become infected in the $\mathcal U$-KCM in finite time starting from ${\eta }(0)=({\mathbb {1}} _{x\not \in A})_{x\in {\mathbb Z} ^d}$. Strictly speaking, other than this notation, bootstrap percolation does not feature in our proofs, but its intuition and techniques are omnipresent. On the other hand, some of our intermediate results can translate directly to recover some bootstrap percolation results of [7, 8].

### Universality setting

We direct the reader to the companion paper by Marêché and the author [20], a monograph of Toninelli and the author [26] and the author’s PhD thesis [19, Chap. 1], for comprehensive background on the universality results for two-dimensional KCM and their history. Instead, we provide a minimalist presentation of the notions we need. The definitions in this section were progressively accumulated in [7, 8, 15, 20, 22, 31] and may differ in phrasing from the originals, but are usually equivalent thereto (see [20] for more details).

Henceforth, we restrict our attention to models in two dimensions. The Euclidean norm and scalar product are denoted by $\Vert \cdot \Vert$ and $\langle \cdot ,\cdot \rangle$, and distances are w.r.t. $\Vert \cdot \Vert$. Let $S^1=\{x\in {\mathbb R} ^2:\Vert x\Vert =1\}$ be the unit circle consisting of *directions*, which we occasionally identify with ${\mathbb R} /2\pi {\mathbb Z}$ in the standard way. We denote the open half plane with outer normal $u\in S^1$ and offset $l\in {\mathbb R}$ by

3 $$\begin{aligned} {\mathbb H} _u(l)=\left\{ x\in {\mathbb R} ^2:\langle x,u\rangle < l\right\} \end{aligned}$$

and omit *l* if it is 0. We further denote its closure by ${\overline{{\mathbb H} }}_u(l)$, omitting zero offsets. We often refer to continuous sets such as ${\mathbb H} _u$, but whenever talking about infections or sites in them, we somewhat abusively identify them with their intersections with ${\mathbb Z} ^2$ without further notice.

Fix an update family $\mathcal U$.

#### Definition 1.1

(*Stability*). A direction $u\in S^1$ is *unstable* if there exists $U\in \mathcal U$ such that $U\subset {\mathbb H} _u$ and *stable* otherwise.

It is not hard to see that unstable directions form a finite union of finite open intervals in $S^1$ [8, Theorem 1.10]. We say that a stable direction is *semi-isolated* (resp. *isolated*) if it is the endpoint of a nontrivial (resp. trivial) interval of stable directions.

#### Definition 1.2

(*Criticality*). Let $\mathcal C$ be the set of open semicircles of $S^1$. An update family is

- *supercritical* if there exists $C\in \mathcal C$ such that all $u\in C$ are unstable;
- *subcritical* if every semicircle contains infinitely many stable directions;
- *critical* otherwise.

The following notion measures “how stable” a stable direction is.

#### Definition 1.3

(*Difficulty*). For $u\in S^1$ the *difficulty* ${\alpha }(u)$ of *u* is

- 0 if *u* is unstable;
- $\infty$ if *u* is stable, but not isolated;
- $$\min \{n:\exists Z\subset {\mathbb Z} ^2,|Z|=n,|[{\mathbb H} _u\cup Z]_\mathcal U{\setminus }{\mathbb H} _u|=\infty \}$$ otherwise.

The *difficulty* of $\mathcal U$ is

$$\begin{aligned} {\alpha }=\min _{C\in \mathcal C}\max _{u\in C}{\alpha }(u). \end{aligned}$$

We say that a direction $u\in S^1$ is *hard* if ${\alpha }(u)>{\alpha }$.

**Fig. 1:**
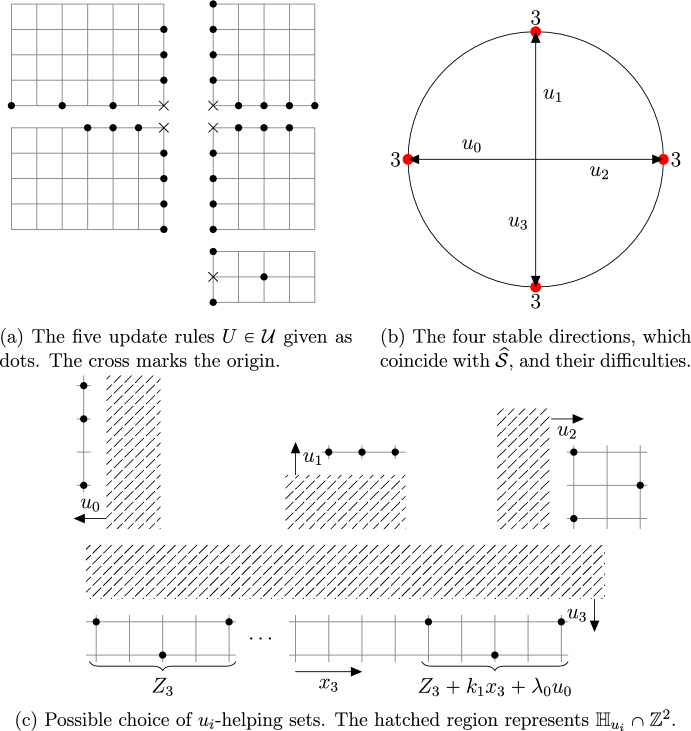
An intricate isotropic example

See Fig. 1 for an example update family with ${\alpha }=3$ and its difficulties. It can be shown that ${\alpha }(u)\in [1,\infty )$ for isolated stable directions [7, Lemma 2.8]. Consequently, a model is critical iff $0<{\alpha }<\infty$ and supercritical iff ${\alpha }=0$, so difficulty is tailored for critical models and refines Definition 1.2. Furthermore, for supercritical models the notions of stable and hard direction coincide. Finally, observe that the definition implies that for any critical or supercritical update family there exists an open semicircle with no hard direction.

#### Definition 1.4

(*Refined types*). A critical or supercritical update family is

- *rooted* if there exist two non-opposite hard directions;
- *unrooted* if it is not rooted;
- *unbalanced* if there exist two opposite hard directions;
- *balanced* if it is not unbalanced, that is, there exists a *closed* semicircle containing no hard direction.

We further partition balanced unrooted update families into

- *semi-directed* if there is exactly one hard direction;
- *isotropic* if there are no hard directions.

We further consider the distinction between models with finite and infinite number of stable directions. The latter being necessarily rooted, but possibly balanced or unbalanced, we end up with a partition of all (two-dimensional non-subcritical) families into the seven classes studied in detail below in the critical case. We invite the interested reader to consult [20, Fig. 1] for simple representatives of each class with rules contained in the the lattice axes and reaching distance at most 2 from the origin. Naturally, many more examples have been considered in the literature (also see Fig. 1).

Let us remark that models in each class may have one axial symmetry, but non-subcritical models invariant under rotation by $\pi$ are necessarily either isotropic or unbalanced unrooted (thus with finite number of stable directions), while invariance by rotation by $\pi /2$ implies isotropy.

### Results

Our result, summarised in Table 1, together with the companion paper by Marêché and the author [20], is the following complete refined classification of two-dimensional critical KCM (for the classification of supercritical ones, which only features the rooted/unrooted distinction, see [29–31]).

**Table 1:**
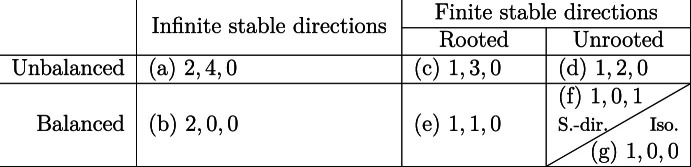
Classification of critical $\mathcal U$-KCM with difficulty $\alpha$. For each class ${\mathbb E} _{{\mu }}[{\tau }_0]=\exp \left( \Theta (1)\left( \frac{1}{q^\alpha }\right) ^{\beta }\left( \log \frac{1}{q}\right) ^\gamma \left( \log \log \frac{1}{q}\right) ^\delta \right)$ as $q \rightarrow 0$. The label of the class and the exponents $\beta ,\gamma ,\delta$ are indicated in that order

#### Theorem 1

(Universality classification of two-dimensional critical KCM). Let $\mathcal U$ be a two-dimensional critical update family with difficulty $\alpha$. We have the following exhaustive alternatives as $q\rightarrow 0$ for the expected infection time of the origin under the stationary $\mathcal U$-KCM.^1^ If $\mathcal U$ is

- unbalanced with infinite number of stable directions (so rooted), then
  $$\begin{aligned} {\mathbb E} _{{\mu }}[{\tau }_0]=\exp \left( \frac{\Theta \left( \left( \log (1/q)\right) ^4\right) }{q^{2\alpha }}\right) ; \end{aligned}$$
- balanced with infinite number of stable directions (so rooted), then
  $$\begin{aligned} {\mathbb E} _{{\mu }}[{\tau }_0]=\exp \left( \frac{\Theta (1)}{q^{2\alpha }}\right) ; \end{aligned}$$
- unbalanced rooted with finite number of stable directions, then
  $$\begin{aligned} {\mathbb E} _{{\mu }}[{\tau }_0]=\exp \left( \frac{\Theta \left( \left( \log (1/q)\right) ^3\right) }{q^{\alpha }}\right) ; \end{aligned}$$
- unbalanced unrooted (so with finite number of stable directions), then
  $$\begin{aligned} {\mathbb E} _{{\mu }}[{\tau }_0]=\exp \left( \frac{\Theta \left( \left( \log (1/q)\right) ^2\right) }{q^{\alpha }}\right) ; \end{aligned}$$
- balanced rooted with finite number of stable directions, then^2^
  $$\begin{aligned} {\mathbb E} _{{\mu }}[{\tau }_0]=\exp \left( \frac{\Theta \left( \log (1/q)\right) }{q^{\alpha }}\right) ; \end{aligned}$$
- semi-directed (so balanced unrooted with finite number of stable directions), then
  $$\begin{aligned} {\mathbb E} _{{\mu }}[{\tau }_0]=\exp \left( \frac{\Theta \left( \log \log (1/q)\right) }{q^{\alpha }}\right) ; \end{aligned}$$
- isotropic (so balanced unrooted with finite number of stable directions), then
  $$\begin{aligned} {\mathbb E} _{{\mu }}[{\tau }_0]=\exp \left( \frac{\Theta (1)}{q^{\alpha }}\right) . \end{aligned}$$

This theorem is the result of a tremendous amount of effort by a panel of authors. It would be utterly unfair to claim that it is due to the present paper and its companion [20] alone. Indeed, parts of the result (sharp upper or lower bounds for certain classes) were established by (subsets of) Marêché, Martinelli, Morris, Toninelli and the author [21, 22, 31, 32]. Moreover, particularly for the lower bounds, the classification of two-dimensional critical $\mathcal U$-bootstrap percolation models by Bollobás, Duminil-Copin, Morris and Smith [7] (featuring only the balanced/unbalanced distinction) is heavily used, while upper bounds additionally use prerequisites from [23, 24]. Thus, a fully self-contained proof of Theorem 1 from common probabilistic background is currently contained only in all the above references combined and spans hundreds of pages. Our contribution is but the conclusive step.

More precisely, the lower bound for classes (d) and (g) was deduced from [7] in [32]; the lower bound for class (b) was established in [21], while the remaining four were proved in [20]. Turning to upper bounds, the one for class (a) was given in [31] and the one for class (c) is due to [22]. The remaining five upper bounds are new and those are the subject of our work. The most novel and difficult ones concern classes (e) and (f), the latter remaining quite mysterious prior to our work. Indeed, [22, Conjecture 6.2] predicted the above result with the exception of this class, whose behaviour was unclear. We should note that this conjecture itself rectified previous ones from [31, 34], which were disproved by the unexpected result of [22], and was new to physicists, as well as mathematicians.

#### Remark 1.5

It should be noted that universality results including Theorem 1 apply to KCM more general than the ones defined in Sect. 1.1. Namely, we may replace $c_x$ in Eq. (2) by a fixed linear combination of the constraints $c_x$ associated to any finite set of update families. For instance, we may update vertices at rate proportional to their number of infected neighbours. This and other models along these lines have been considered e.g. in [2, 5, 12]. For such mixtures of families, the universality class is determined by the family obtained as their union. Indeed, upper bounds follow by direct comparison of the corresponding Dirichlet forms, while lower bounds (e.g. [20]) generally rely on deterministic bottlenecks, which remain valid.

#### Remark 1.6

Let us note that for reasons of extremely technical nature, we do not provide a full proof of (the upper bound of) Theorem 1(e). More precisely, we prove it as stated for models with rules contained in the axes of the lattice. We also prove a fully general upper bound of

4 $$\begin{aligned} \exp \left( \frac{O(\log (1/q))\log \log \log (1/q)}{q^{\alpha }}\right) . \end{aligned}$$

Furthermore, with very minor modifications (see Remark 7.1), the error factor can be reduced from $\log \log \log$ to $\log _*$, where $\log _*$ denotes the number of iterations of the logarithm before the result becomes negative (the inverse of the tower function). Unfortunately, removing this minuscule error term requires further work, which we omit for the sake of concision. Instead, we provide a sketch of how to achieve this in “Appendix C”.

### Organisation

The paper is organised as follows. In Sect. 2 we begin by outlining all the relevant relaxation mechanisms used by critical KCM, providing detailed intuition for the proofs to come. This section is particularly intended for readers unfamiliar with the subject, as well as physicists, for whom it may be sufficiently convincing on its own. In Sect. 3 we gather various notation and simple preliminaries.

In Sect. 4 we formally state the two fundamental techniques we use to move from one scale to the next, namely East-extensions and CBSEP-extensions, which import and generalise ideas of [24]. They are used in various combinations throughout the rest of the paper. The proofs of the results about those extensions, including the microscopic dynamics treated by [18] are deferred to “Appendix A”, since they are quite technical and do not require new ideas. The bounds arising from extensions feature certain conditional expectations. We provide technical tools for estimating them in Sect. 4.4. We leave the entirely new proofs of these general analogues of [24, Appendix A] to “Appendix B”.

Sections 5–9 are the core of our work and use the extensions mentioned above to prove the upper bounds of Theorem 1 for classes (g), (d), (f), (e), (b) respectively. As we will discuss in further detail (see Sect. 2 and Table 2b), some parts of the proofs are common to several of these classes, making the sections interdependent. Thus, they are intended for linear reading.

We conclude in “Appendix C” by explaining how to remove the corrective $\log \log \log (1/q)$ factor discussed in Remark 1.6 to recover the result of Theorem 1(e) as stated in full generality. Due to their technical nature, we delegate Appendices A to C to the arXiv version of the present work.

Familiarity with the companion paper [20] or bootstrap percolation [7] is not needed. Inversely, familiarity with [22, 24] is strongly recommended for going beyond Sect. 2 and achieving a complete view of the proof of the upper bounds of Theorem 1. Nevertheless, we systematically state the implications of intermediate results of those works for our setting in a self-contained fashion, without re-proving them.

## Mechanisms

In this section we attempt a heuristic explanation of Theorem 1 from the viewpoint of mechanisms, which is mostly related to upper bound proofs. Yet, let us say a few words about the lower bounds. The proof of the lower bounds in the companion paper [20] has the advantage and disadvantage of being unified for all seven classes. This is undeniably practical and spotlights the fact that all scaling behaviours can be viewed through the lens of the same bottleneck (few energetically costly configurations through which the dynamics has to go to infect the origin) on a class-dependent length scale. However, the downside is that it provides little insight on the particularities of each class, which turn out to be quite significant. To prove upper bounds we need a clear vision of an efficient mechanism for infecting the origin in each class. Since we work with the stationary process, efficient means that it should avoid configurations which are too unlikely w.r.t. ${\mu }$. However, while lower bounds only identify what cannot be avoided, they do not tell us how to avoid everything else, nor indeed how to reach the unavoidable bottleneck.

Instead of outlining the mechanism used by each class, we focus on techniques which are somewhat generic and then apply combinations thereof to each class. In figurative terms, we develop several computer hardware components (three processors, four RAMs, etc.), give a general scheme of how to compose a generic computer out of generic components and, finally, assemble seven concrete computer configurations, using the appropriate components for each, sometimes changing a single component from a machine to the other. Moreover, within each component type different instances are strictly comparable, so, at the assembly stage, we might simply choose the best possible component fitting with the requirements of model at hand. This enables us to highlight the robust tools developed and refined recently, which correspond to the components and how they are manufactured, as well as give a clean universal proof scheme into which they are plugged.

Our different components are called the *microscopic, internal, mesoscopic and global dynamics* and correspond to progressively increasing length scales on which we are able to relax, given a suitable infection configuration. As the notion of “suitable,” which we call *super good* (SG), depends on the class and lower scale mechanisms used, we mostly use it as a black box input extended progressively over scales in a recursive fashion.

**Table 2:** Summary of the mechanisms and their costs. The microscopic mechanism common to all classes and with negligible cost is not shown (see Sect. 2.2)

(a)The relaxation time cost associated to each choice of dynamics mechanism on each scale in terms of the probability of a droplet ${\rho }_{\textrm{D}}$.							
Global		Mesoscopic		Internal			
CBSEP	East	CBSEP	East, Stair	CBSEP	East	Unbal.	
${\rho }_{\textrm{D}}^{-1+o(1)}$	${\rho }_{\textrm{D}}^{-O(\log (1/{\rho }_{\textrm{D}}))}$	$e^{q^{-o(1)}}$	${\rho }_{\textrm{D}}^{-O(\log (1/q))}$	$e^{q^{-o(1)}}$	${\rho }_{\textrm{D}}^{-O(\log \log (1/q))}$	${\rho }_{\textrm{D}}^{-O(1)}$	
(b)The fastest mechanism available to each class of Theorem 1 on each scale. The * indicates a leading contribution for the class (column).							
	(a)	(b)	(c)	(d)	(e)	(f)	(g)
Global	East*	East*	CBSEP	CBSEP*	CBSEP	CBSEP	CBSEP*
Mesoscopic	Stair	East	East*	CBSEP	East*	CBSEP	CBSEP
Internal	–	East	Unbal.	Unbal.*	East	East*	CBSEP

In order to guide the reader through Sect. 2 and beyond, in Table 2, we summarise the optimal mechanisms for each universality class on each scale and its cost. While its full meaning will only become clear in Sect. 2.7, the reader may want to consult it regularly, as they progress through Sect. 2.

**Fig. 2:**
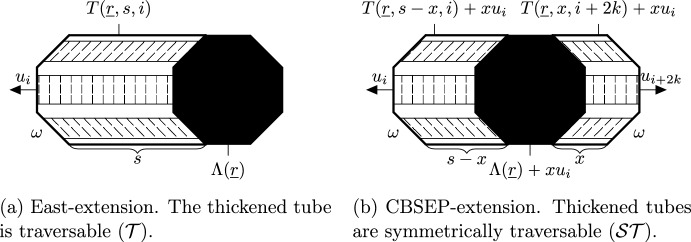
One-directional extensions. The black droplet is SG. Helping sets appear on each line of the hatched parallelograms as indicated by the hatching direction. The white strips have width $\Theta (C^2)$

The SG events concern certain convex polygonal geometric regions called *droplets*. These events are designed so as to satisfy several conditions ensuring that the configuration of infections inside the droplet is sufficient to infect the entire droplet. The SG events defined by extensions from smaller to larger scales require the presence of a lower scale droplet inside the large one (see Fig. 2) in addition to well-chosen more sparse infections called *helping sets* in the remainder of the larger scale droplet (see Fig. 1). Helping sets allow the smaller one to move inside the bigger one.

We say that a droplet *relaxes* in a certain *relaxation time* if the dynamics restricted to the SG event and to this region “mixes” in this much time. Formally, this translates to a constrained Poincaré inequality for the conditional measure, but this is unimportant for our discussion.

One should think of droplets as extremely unlikely objects, which are able to move within a slightly favourable environment. Indeed, at all stages of our treatment, we need to control the inverse probability of droplets being SG and their relaxation times, keeping them as small as feasible. Furthermore, due to their inductive definition, the favourable environment required for their movement should not be too costly. Indeed, that would result in the deterioration of the probability of larger scale droplets, as those incorporate the lower scale environment in their internal structure. Hence, we seek a balance between asking for many infections to make the movement efficient and asking for few in order to keep the probability of droplets high enough.

### Scales

**Microscopic dynamics** refers to modifying infections at the level of the lattice *along the boundary of a droplet*, while respecting the KCM constraint.

**Internal dynamics** refers to relaxation on scales from the lattice level to the *internal scale* $\ell ^{\textrm{int}}= C^2\log (1/q)/q^{\alpha }$, where *C* is a large constant depending on $\mathcal U$. This is the most delicate and novel step. Up to $\ell ^{\textrm{int}}$ we account for the main contribution to the probability of droplets. That is, at all larger scales the probability of a droplet essentially saturates at a certain value ${\rho }_{\textrm{D}}$, because finding helping sets becomes likely. Thus, on smaller scales, it is important to only very occasionally ask for more than ${\alpha }$ infections to appear close to each other in order to get the right probability ${\rho }_{\textrm{D}}$. This means that up to the internal scale hard directions are practically impenetrable, since they require helping sets of more that ${\alpha }$ infections.

**Mesoscopic dynamics** refers to relaxation on scales from $\ell ^{\textrm{int}}$ to the *mesoscopic scale* $\ell ^{\textrm{mes}}= 1/q^C$. As our droplets grow to the mesoscopic scale and past it, it becomes possible to require larger helping sets, which we call *W*-*helping sets*. These allow droplets to move also in hard directions of finite difficulty, while nonisolated stable directions are still blocking.

**Global dynamics** refers to relaxation on scales from $\ell ^{\textrm{mes}}$ to infinity. The extension to infinity being fairly standard (and not hard), one should rather focus on scales up to the *global scale* given by $\ell ^{\textrm{gl}}=\exp (1/q^{3{\alpha }})$, which is notably much larger than all time scales we are aiming for, but otherwise rather arbitrary.

Roughly speaking, on each of the last three scales, one should decide how to move a droplet of the lower scale in a domain on the larger scale.

For simplicity, in the remainder of Sect. 2, we assume that the only four relevant directions are the axis ones so that droplets have rectangular shape (see Sect. 3.3). We further assume that all directions in the left semicircle have difficulties at most ${\alpha }$, while the down direction is hard, unless there are no hard directions (isotropic class).

### Microscopic dynamics

The microscopic dynamics (see “Appendix A.2”) is the only place where we actually deal with the KCM directly and is the same, regardless of the size of the droplet and the universality class. Roughly speaking, from the outside of the droplet, we may think of it as fully infected, since it is able to relax and, therefore, bring infections where they are needed. Thus, the outer boundary of the droplet behaves like a 1-dimensional KCM with update family reflecting that we view the droplet as infected. Hence, provided there are enough helping sets at the boundary to infect it, we can apply results on 1-dimensional KCM supplied for this purpose by the author [18].

This way we establish that one additional column can relax in time $\exp (O(\log (1/q))^2)$, similarly to the East model described in Sect. 2.3.2 below. Assuming we know how to relax on the droplet itself, this allows us to relax on a droplet with one column appended. However, applying this procedure recursively line by line is not efficient enough to be useful for extending droplets more significantly.

### One-directional extensions

We next explain two fundamental techniques beyond the microscopic dynamics which we use to extend droplets on any scale in a single direction (see Sect. 4).

As mentioned above, our droplets are polygonal regions with a SG event (presence of a suitable arrangement of infections in the droplet). An extension takes as input a droplet and produces another one. In terms of geometry, it contains the original one and is obtained by extending it, say, horizontally, either to the left or both left and right (see Fig. 2). The extended droplet’s SG event requires that the smaller one is SG and, additionally, certain helping sets appear in the remaining volume. The choice of where we position the smaller droplet (at the right end of the bigger one, or anywhere inside it) depends on the type of extension. The additional helping sets are required in such a way that, with their help, the smaller droplet can, in principle, completely infect the larger one and, therefore, make it relax (resample its configuration within its SG event).

Thus, an *extension* is a procedure for iteratively defining SG events on larger and larger scales. For each of our two types of extensions we need to provide corresponding iterative bounds on the probability of the SG event and on the relaxation time of droplets on this event. The former is a matter of careful computation. For the latter task we intuitively use a large-scale version of an underlying one-dimensional spin model, which we describe first.

#### CBSEP-extension

In the one-dimensional spin version of CBSEP [23, 24] we work on $\{\uparrow ,\downarrow \}^{\mathbb Z}$. At rate 1 we resample each pair of neighbouring spins, provided that at least one of them is $\uparrow$. Their state is resampled w.r.t. the reference product measure, which is reversible, conditioned to still have a $\uparrow$ in at least one of the two sites. In other words, $\uparrow$ can perform coalescence, branching and symmetric simple exclusion moves, hence the name. The relaxation time of this model on volume *V* is roughly $\min (V,1/q)^{2}$ in one dimension and $\min (V,1/q)$ in two and more dimensions [23, 24], where *q* is the equilibrium density of $\uparrow$, which we think of as being small.

For us $\uparrow$ represent SG droplets, which we would like to move within a larger volume. However, as we would like them to be able to move possibly by an amount smaller than the size of the droplet, we need to generalise the model a bit. We equip each site of a finite interval of ${\mathbb Z}$ with a state space corresponding to the state of a column of the height of our droplet of interest in the original lattice ${\mathbb Z} ^2$. Then the event “there is a SG droplet” may occur on a group of $\ell$ consecutive sites (columns). The long range generalised CBSEP, which, abusing notation, we call CBSEP, is defined as follows. We fix some *range* $R>\ell$ and resample at rate 1 each group of *R* consecutive sites, if they contain a SG droplet. The resampling is performed conditionally on preserving the presence of a SG droplet in those *R* sites. Thus, one move of this process essentially delocalises the droplet within the range.

It is important to note (and this was crucial in [24]) that CBSEP does not *have to* create an additional droplet in order to evolve. Since SG droplets are unlikely, it suffices to move an initially available SG droplet through our domain in order to relax. Since infection needs to be able to propagate both left and right from the SG droplets, we will define (see Sect. 4.3 and particularly Definition 4.7, Fig. 2b) *CBSEP-extension* by extending our domain horizontally and asking for the SG droplet anywhere inside with suitable “rightwards-pointing” helping sets on its right and “leftwards-pointing” on its left.

**Fig. 3:**
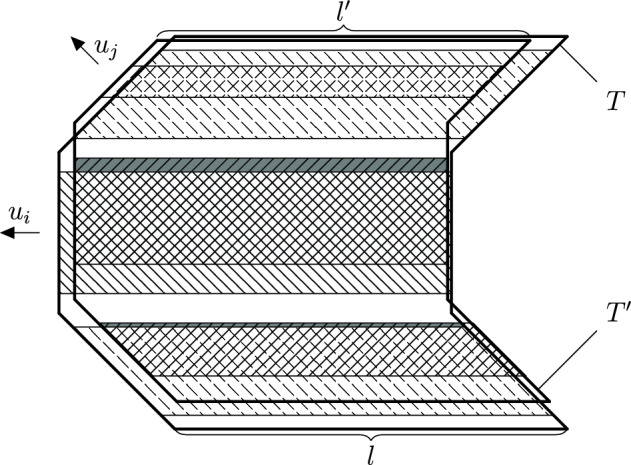
Illustration of the perturbation of Lemma 4.11. The two thickened tubes are *T* and $T'$. The regions concerned by their traversability are hatched in different directions

**Fig. 4:**
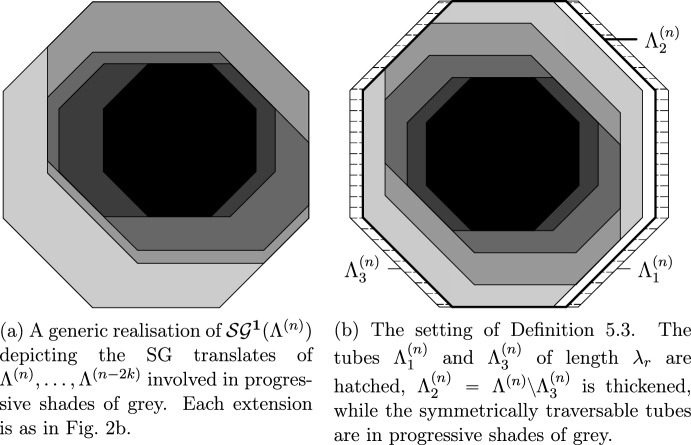
Geometry of isotropic $\mathcal S\mathcal G$ and $\overline{\mathcal S\mathcal G}$ events

While we now know that droplets evolve according to CBSEP, it remains to see how one can reproduce one CBSEP move via the original dynamics. This is done inductively on *R* by a bisection procedure, the trickiest part being the case $R=\ell +1$. We then dispose with a droplet plus one column—exactly the setting for microscopic dynamics. However, we not only want to resample the state of the additional column, but also allow the droplet to move by one lattice step. To achieve this, we have to look inside the structure of the SG droplet and require for its infections (which have no rigid structure and may therefore move around like the organelles of an amoeba) to be somewhat more on the side we want to move towards (see e.g. Fig. 4 and also Definitions 5.3, 6.5, 7.7 and 7.8). Then, together with a suitable configuration on the additional column provided by the microscopic dynamics, we easily recover our SG event shifted by one step, since most of the structure was already provided by the version of the SG event “contracted” towards the new column.

This amoeba-like motion (moving a droplet, by slightly rearranging its internal structure) leads to a very small relaxation time of the dynamics. Indeed, the time needed to move the droplet is the product of three contributions: the relaxation time of the 1-dimensional spin model; the relaxation time of the microscopic dynamics; the time needed to see a droplet contracting as explained above (see Proposition 4.9). The first of these is a power of the volume (number of sites); the second is $\exp (O(\log (1/q)))^2)$; the third is also small, as we discuss in Sect. 2.3.2.

However, CBSEP-extensions can only be used for sufficiently symmetric update families. That is, the droplet needs to be able to move indifferently both left and right and its position should not be biased in one direction or the other. Specifically, if we are working on a scale that requires the use of helping sets of size $\alpha$, these have to exist both for the left and right directions, so the model needs to be unrooted (if instead we use larger helping sets, having a finite number of stable directions suffices). The reason is that otherwise the position of the SG droplet is biased in one direction instead of being approximately uniform. This would break the analogy with the original one-dimensional spin model, which is totally symmetric. When symmetry is not available, we recourse to the East-extension presented next, which may also be viewed as a totally asymmetric version of the CBSEP-extension.

#### East-extension

The East model [27] is the one-dimensional KCM with $\mathcal U=\{\{1\}\}$. That is, we are only allowed to resample the left neighbour of an infection. An efficient recursive mechanism for its relaxation is the following [33]. Assume we start with an infection at 0. In order to bring an infection to $-2^n+1$, using at most *n* infections at a time (excluding 0), we first bring one to $-2^{n-1}+1$, using $n-1$ infections. We then place an infection at $-2^{n-1}$ and reverse the procedure to remove all infections except 0 and $-2^{n-1}$. Finally, start over with $n-1$ infections, viewing $-2^{n-1}$ as the new origin, thus reaching $-2^{n}+1$. It is not hard to check that this is as far as one can get with *n* infections [11]. Thus, a number of infections logarithmic in the desired distance is needed. This is to be contrasted with CBSEP, for which only one infection is ever needed, as it can be moved indefinitely by SEP moves. The relaxation time of East on a segment of length *L* is $q^{-O(\log \min (L,1/q))}$ [1, 9, 10], where *q* is the equilibrium density of infections. This corresponds to the cost of *n* infections when $2^n\sim \min (L,1/q)$ is the typical distance to the nearest infection.

The long-range generalised version of the East model is defined similarly to that of CBSEP. The only difference is that now $R>\ell$ consecutive columns are resampled together if there is a SG droplet on their extreme right. It is clear that this does not allow *moving* the droplet, but rather forces us to recreate a new droplet at a shifted position before we can progress. The associated *East-extension* of a droplet corresponds to extending its geometry to the left (see Sect. 4.2 and particularly Definition 4.4 and Fig. 2a). The extended SG event requires that the original SG droplet is present in the rightmost position and “leftwards-pointing” helping sets are available in the rest of the extended droplet.

The generalised East process goes back to [31], while the long range version is implicitly used in [22]. However, both works used a brutal strategy consisting of creating the new droplet from scratch. Instead, in this work we have to be much more careful, particularly for semi-directed models. Indeed, take $\ell$ large and $R=\ell +5$. Then it is intuitively clear that the presence of the original rightmost droplet overlaps greatly with the occurrence of the shifted SG one we would like to craft. Hence, the idea is to take advantage of this and only pay the *conditional* probability of the droplet we are creating, given the presence of the original one.

This is not as easy as it sounds for several reasons. Firstly, we should make the SG structure soft enough (in contrast with e.g. [22, 31]) so that small shifts do not change it much. Secondly, we need to actually have a quantitative estimate of the conditional probability of a complicated multi-scale event, given its translated version, which necessarily does not quite respect the same multi-scale geometry. To make matters worse, we do not have at our disposal a very sharp estimate of the probability of SG events (unlike in [24]), so directly computing the ratio of two rough estimates would yield a very poor bound on the conditional probability. In fact, this problem is also present when contracting a droplet in the CBSEP-extension—we need to evaluate the probability of a contracted version of the droplet, conditionally on the original droplet being present.

We deal with these issues in Sect. 4.4 (see also “Appendix B”). We establish that, as intuition may suggest, to create a droplet shifted by $R-\ell$, given the original one, we roughly only need to pay the probability of a droplet on scale $R-\ell$ rather than $\ell$, which provides a substantial gain. Hence, the time necessary for an East-extension of a droplet to relax is essentially the product of the inverse probabilities of a droplet on scales of the form $2^m$ up to the extension length (see Proposition 4.6).

### Internal dynamics

The internal dynamics (see Sects. 5.1, 6.1, 7.1, and 8.1) is where most of our work goes. This is not surprising, as the probability of SG events saturates at its final value ${\rho }_{\textrm{D}}$ at the internal scale. The value of ${\rho }_{\textrm{D}}$ is given by $\exp (-O(1)/q^{\alpha })$ for balanced models and $\exp (-O(\log (1/q))^2/q^{\alpha })$ for unbalanced ones, as in bootstrap percolation [7]. However, relaxation times for some classes keep growing past the internal scale, so the internal dynamics does not necessarily give the final answer in Theorem 1 (see Table 2b).

#### Unbalanced internal dynamics

Let us begin with the simplest case of unbalanced models. If $\mathcal U$ is unbalanced with infinite number of stable directions (class (a)), droplets in [31] on the internal scale consist of several infected consecutive columns, so that no relaxation is needed (the SG event is a singleton). The columns have size $\ell ^{\textrm{int}}$, which justifies the value of ${\rho }_{\textrm{D}}=q^{-O(\ell ^{\textrm{int}})}=\exp (-O(\log (1/q))^2/q^{\alpha })$.

**Fig. 5:**
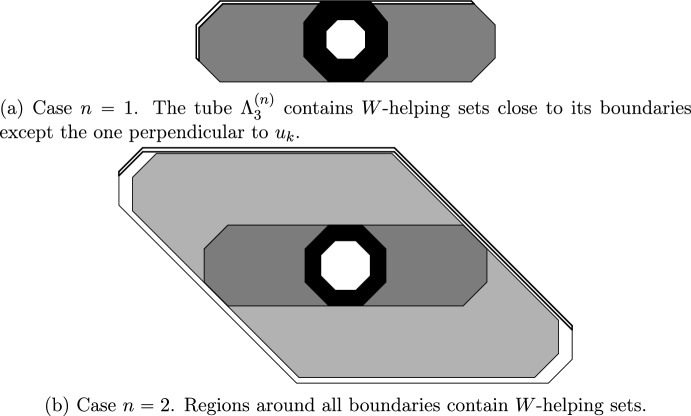
The events $\overline{\mathcal S\mathcal G}({\Lambda }^{(n)}_2)$ and $\overline{\mathcal S\mathcal T}({\Lambda }_3^{(n)})$ of Definition 6.5. ${\Lambda }_3^{(n)}$ is thickened. Black regions are entirely infected. Shaded tubes are $(\textbf{1},W)$-symmetrically traversable

Assume $\mathcal U$ is unbalanced with finite number of stable directions (classes (c) and (d), see Sect. 6.1). Then droplets on the internal scale are fully infected square frames of thickness *O*(1) and size $\ell ^{\textrm{int}}$. That is, the $\ell ^\infty$ ball of radius $\ell ^{\textrm{int}}$ minus the one of radius $\ell ^{\textrm{int}}-O(1)$ (see [22, Figs. 2–4] or Fig. 5 for more general geometry). This frame is infected with probability ${\rho }_{\textrm{D}}=q^{-O(\ell ^{\textrm{int}})}$. In order to relax inside the frame, one can divide its interior into groups of *O*(1) consecutive columns (see [22, Fig. 8]). We can then view them as performing a CBSEP dynamics with $\uparrow$ corresponding to a fully infected group of columns. This is possible, because with the help of the frame each completely infected group is able to completely infect the neighbouring ones. Here we are using that there are finitely many stable directions to ensure both the left and right directions have finite difficulty, so finite-sized helping sets, as provided by the frame, are sufficient to propagate our group of columns. This was already done in [22] and the time necessary for this relaxation is easily seen to be ${\rho }_{\textrm{D}}^{-O(1)}$ (the cost for creating a group of infected columns)—see Proposition 6.2.

#### CBSEP internal dynamics

If $\mathcal U$ is isotropic (class (g), see Sect. 5.1), up to the conditioning problems of Sect. 4.4 described above, we need only minor adaptations of the strategy of [24] for the paradigmatic isotropic model called FA-2f. Droplets on the internal scale have an internal structure as obtained by iterating Fig. 4a (see also [24, Fig. 2]). Our droplets are extended little by little alternating between the horizontal and vertical directions, so that their size is multiplied essentially by a constant at each extension. Thus, roughly $\log (1/q)$ extensions are required to reach $\ell ^{\textrm{int}}$. As isotropic models do not have any hard directions, we can move in all directions and thus the symmetry required for CBSEP-extensions is granted. Hence, this mechanism leads to a very fast relaxation of droplets in time $\exp (q^{-o(1)})$—see Theorem 5.2.^3^

##### Remark 2.1

Note that for CBSEP-extensions to be used, we need a very strong symmetry. Namely, leftwards and rightwards pointing helping sets should be the same up to rotation by $\pi$. Yet, for a general isotropic model we only know that there are no hard directions, so helping sets have the same size (equal to the difficulty $\alpha$ of the model), but not necessarily the same shape. We circumvent this issue by artificially symmetrising our droplets and events. Namely, whenever we require helping sets in one direction, we also require the helping sets for the opposite direction rotated by $\pi$ (see Definitions 3.8,4.1 and 4.7). Although these are totally useless for the dynamics, they are important to ensure that the positions of droplets are indeed uniform rather than suffering from a drift towards an “easier” non-hard direction (see Lemma 4.10).

#### East internal dynamics

The most challenging case is the balanced non-isotropic one (classes (b), (e) and (f)). It is treated in Sects. 7.1 and 8.1, but for the purposes of the present section only Sect. 7.1 is relevant. This is because we assume that only the four axis directions are relevant and our droplets are rectangular. The treatment of the general case for balanced rooted families is left to Sect. 8.1 and “Appendix C” (recall Remark 1.6).

For the internal dynamics the downwards hard direction prevents us from using CBSEP-extensions. To be precise, for semi-directed models (class (f)) it is possible to perform CBSEP-extensions horizontally (and not vertically), but the gain is insignificant, so we treat all balanced non-isotropic models identically up to the internal scale as follows.

**Fig. 6:**
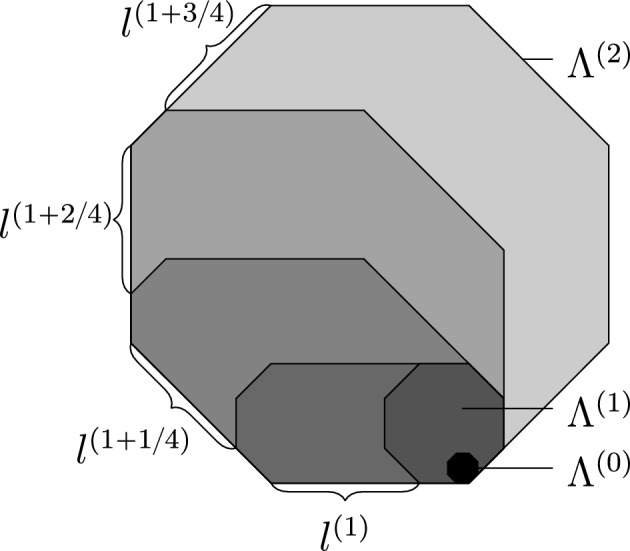
Geometry of the nested droplets ${\Lambda }^{(n)}$ for $k=2$ in the setting of Sect. 7.1. For $n\in {\mathbb N}$ droplets are symmetric and homothetic to the black ${\Lambda }^{(0)}$. Intermediate ones ${\Lambda }^{(1+1/4)}$, ${\Lambda }^{(1+2/4)}$ and ${\Lambda }^{(1+3/4)}$ obtained by East-extensions (see Fig. 2a) in directions $u_0$, $u_1$ and $u_2$ respectively are drawn in progressive shades of grey

We still extend droplets, starting from a microscopic one, by a constant factor alternating between the horizontal and vertical directions (see Fig 6a). However, in contrast with the isotropic case (see Fig. 4a), extensions are done in an oriented fashion, so that the original microscopic droplet remains anchored at the corner of larger ones. Thus, we may apply East-extensions on each step and obtain that the cost is given by the product of conditional probabilities from Sect. 2.3.2 over all scales and shifts of the form $2^n$:

5 $$\begin{aligned} \prod _{n=1}^{\log _2(\ell ^{\textrm{int}})}\prod _{m=0}^{n}a_m^{(n)}, \end{aligned}$$

where $a_m^{(n)}$ is the conditional probability of a SG droplet of size $2^n$ being present at position $2^m$, given that a SG droplet of size $2^n$ is present at position 0. It is crucial that Eq. (5) is *not* the straightforward bound $\prod _{n}({\rho }_{\textrm{D}}^{(n)})^{-n}$, with ${\rho }_{\textrm{D}}^{(n)}$ the probability of a droplet of scale *n*, that one would get by direct analogy with the East model (recall from Sect. 2.3.2 that the relaxation time of East on a small volume *L* is $q^{-O(\log L)}$), as that would completely devastate all our results. Indeed, as mentioned in Sect. 2.3.2, the term $a_m^{(n)}$ in Eq. (5) is approximately equal to ${\rho }_{\textrm{D}}^{(m)}$, rather than ${\rho }_{\textrm{D}}^{(n)}$. This is perhaps one of the most important points to our treatment.

In total, a droplet of size $2^n$ needs to be paid once per scale larger than $2^n$ (see Eq. (42)). A careful computation shows that only droplets larger than $q^{-{\alpha }}$ provide the dominant contribution and those all have probability essentially ${\rho }_{\textrm{D}}=\exp (-O(1)/q^{\alpha })$ (see Eq. (43)). Thus, the total cost would be

6 $$\begin{aligned} \prod _{n=\log (1/q^\alpha )}^{\log (\ell ^{\textrm{int}})}\prod _{m=\log (1/q^\alpha )}^{n}{\rho }_{\textrm{D}}={\rho }_{\textrm{D}}^{O(\log \log (1/q))^2}=\exp \left( -O(\log \log (1/q))^2/q^\alpha \right) , \end{aligned}$$

since there are $\log \log (1/q)$ scales from $q^{-{\alpha }}$ to $\ell ^{\textrm{int}}$, as they increase exponentially.

Equation (6) is unfortunately a bit too rough for the semi-directed class, overshooting Theorem 1(f). However, the solution is simple. It suffices to introduce scales growing double-exponentially above $q^{-{\alpha }}$ instead of exponentially (see Eq. (37)), so that the product over scales *n* in Eq. (6) becomes dominated by its last term, corresponding to droplet size $\ell ^{\textrm{int}}$. This gives the optimal final cost

$$\begin{aligned} {\rho }_{\textrm{D}}^{-\log (q^\alpha \ell ^{\textrm{int}})}={\rho }_{\textrm{D}}^{-O(\log \log (1/q))}=\exp \left( O(\log \log (1/q))/q^{\alpha }\right) \end{aligned}$$

(see Theorem 7.3).

### Mesoscopic dynamics

For the mesoscopic dynamics (see Sects. 5.1, 6.2, 7.2, and 9.1) we are given as input a SG event for droplets on scale $\ell ^{\textrm{int}}=C^2\log (1/q)/q^\alpha$ and a bound on their relaxation time and occurrence probability ${\rho }_{\textrm{D}}$. We seek to output the same on scale $\ell ^{\textrm{mes}}=q^{-C}$. Taking $C\gg W$, once our droplets have size $\ell ^{\textrm{mes}}$, we are able to find *W*-helping sets (sets of *W* consecutive infections, where *W* is large enough).

#### CBSEP mesoscopic dynamics

If $\mathcal U$ is unrooted (classes (d), (f) and (g), see Sects. 6.2 and 7.2), recall that the hard directions (if any) are vertical. Then we can perform a horizontal CBSEP-extension directly from $\ell ^{\textrm{int}}$ to $\ell ^{\textrm{mes}}$, since $\ell ^{\textrm{int}}=C^2\log (1/q)/q^{\alpha }$ makes it likely for helping sets (of size $\alpha$) to appear along all segments of length $\ell ^{\textrm{int}}$ until we reach scale $\ell ^{\textrm{mes}}= q^{-C}$. The resulting droplet is very wide, but short (see Fig. 5a). However, this is enough for us to be able to perform a vertical CBSEP-extension (see Fig. 5b), requiring *W*-helping sets, since they are now likely to be found. Again, CBSEP dynamics being very efficient, its cost is negligible. Note that, in order to perform the vertical extension, we are using that there are no nonisolated stable directions, so that *W* is larger than the difficulty of the up and down directions, making *W*-helping sets sufficient to induce growth in those directions. Thus, morally, there are no hard directions beyond scale $\ell ^{\textrm{mes}}$ for unrooted models.

#### East mesoscopic dynamics

If $\mathcal U$ is rooted (classes (a)–(c) and (e), see Sect. 9.1), CBSEP-extensions are still inaccessible. We may instead East-extend horizontally from $\ell ^{\textrm{int}}$ to $\ell ^{\textrm{mes}}$ in a single step. If the model is balanced or has a finite number of stable directions (classes (b), (c) and (e)), we may proceed similarly in the vertical direction, reaching a droplet of size $\ell ^{\textrm{mes}}$ in time ${\rho }_{\textrm{D}}^{-O(\log (1/q))}$ (here we use the basic bound $q^{-O(\log L)}$ for East dynamics recalled in Sect. 2.3.2, which is fairly tight in this case, since droplets are small compared to the volume: $\log \ell ^{\textrm{mes}}\approx \log (\ell ^{\textrm{mes}}/\ell ^{\textrm{int}})$). For the unbalanced case (class (c)) here we require *W*-helping sets along the long side of the droplet like in Sect. 2.5.1. Another way of viewing this is simply as extending the procedure used for the East internal dynamics all the way up to the mesoscopic scale $\ell ^{\textrm{mes}}$ (see Sect. 9.1).

It should be noted that a version of this mechanism, which coincides with the above for models with rectangular droplets, but differs in general, was introduced in [22]. Though their *snail mesoscopic dynamics* can be replaced by our East one, for the sake of concision in Sect. 8.2 we directly import the results of [22] based on the snail mechanism.

#### Stair mesoscopic dynamics

For unbalanced families with infinite number of stable directions (class (a)) the following *stair mesoscopic dynamics* was introduced in [31]. Recall from Sect. 2.4.1 that for unbalanced models the internal droplet is simply a fully infected frame or group of consecutive columns. While moving the droplet left via an East motion, we pick up *W*-helping sets above or below the droplet. These sets allow us to make all droplets to their left shifted up or down by one row. Hence, we manage to create a copy of the droplet far to its left but also slightly shifted up or down (see [31, Fig. 6]. Repeating this (with many steps in our staircase) in a two-dimensional East-like motion, we can now relax on a mesoscopic droplet with horizontal dimension much larger than $\ell ^{\textrm{mes}}$ but still polynomial in 1/*q* and vertical dimension $\ell ^{\textrm{mes}}$ in time ${\rho }_{\textrm{D}}^{-O(\log (1/q))}$. Here, one should again intuitively imagine we are using the bound $q^{-O(\log L)}$ but this time for the relaxation time of the 2-dimensional East model.

### Global dynamics

The global dynamics (see Sects. 5.2, 6.3, 7.3, 8.2 and 9.2) receives as input a SG event for a droplet on scale $\ell ^{\textrm{mes}}$ with probability roughly ${\rho }_{\textrm{D}}$ and a bound on its relaxation time, as provided by the mesoscopic dynamics. Its goal is to move such a droplet efficiently to the origin from its typical initial position at distance roughly ${\rho }_{\textrm{D}}^{-1/2}$.

#### CBSEP global dynamics

If $\mathcal U$ has a finite number of stable directions (classes (c)–(g)) the mesoscopic droplet can perform a CBSEP motion in a typical environment. Indeed, the droplet is large enough for CBSEP-extensions with *W*-helping sets to be possible in all directions. Therefore, the cost of this mechanism is given by the relaxation time of CBSEP on a box of size $\ell ^{\textrm{gl}}=\exp (1/q^{3\alpha })$ with density of $\uparrow$ given by ${\rho }_{\textrm{D}}$. Performing this strategy carefully and using the 2-dimensional CBSEP, this yields a relaxation time $\min ((\ell ^{\textrm{gl}})^2,1/{\rho }_{\textrm{D}})=1/{\rho }_{\textrm{D}}$ (recall Sect. 2.3.1 and see Sect. 5.2).

#### East global dynamics

If $\mathcal U$ has infinite number of stable directions (classes (a) and (b)), the strategy is identical to the CBSEP global dynamics, but employs an East dynamics. Now the cost becomes the relaxation time of an East model with density of infections ${\rho }_{\textrm{D}}$, which yields a relaxation time of ${\rho }_{\textrm{D}}^{-O(\log \min (\ell ^{\textrm{gl}},1/{\rho }_{\textrm{D}}))}={\rho }_{\textrm{D}}^{-O(\log (1/{\rho }_{\textrm{D}}))}$ (recall Sect. 2.3.2 and see Sect. 9.2).

### Assembling the components

To conclude, let us return to the summary provided in Table 2. In Table 2a we collect the mechanisms for each scale and their cost to the relaxation time. The results are expressed in terms of the probability of a droplet ${\rho }_{\textrm{D}}$, which equals $\exp (-O(\log (1/q))^2/q^{\alpha })$ for unbalanced models and $\exp (-O(1)/q^{\alpha })$ for balanced ones. The final bound on ${\mathbb E} _{{\mu }}[{\tau }_0]$ for each class then corresponds to the product of the costs of the mechanism employed at each scale. To complement this, in Table 2b we indicate the fastest mechanism available for each class on each scale. We further indicate which one gives the dominant contribution to the final result appearing in Theorem 1, once the bill is footed.

Finally, let us alert the reader that, for the sake of concision, the proof below does not systematically implement the optimal strategy for each class as indicated in Table 2b if that does not deteriorate the final result. Similarly, when that is unimportant, we may give weaker bounds than the ones in Table 2a. In Sect. 8.2 we tacitly import a weaker precursor of the CBSEP global mechanism from [22] not listed above.

## Preliminaries

### Harris inequality

Let us recall a well-known correlation inequality due to Harris [17]. It is used throughout and we state some particular formulations that are useful to us.

For Sect. 3.1 we fix a finite ${\Lambda }\subset {\mathbb Z} ^2$. We say that an event $\mathcal A\subset {\Omega }_{\Lambda }$ is *decreasing* if adding infections does not destroy its occurrence.

#### Proposition 3.1

(Harris inequality). Let $\mathcal A,\mathcal B\subset {\Omega }_{\Lambda }$ be decreasing. Then

7 $$\begin{aligned} {\mu }(\mathcal A\cap \mathcal B)\geqslant {\mu }(\mathcal A){\mu }(\mathcal B). \end{aligned}$$

#### Corollary 3.2

Let $\mathcal A,\mathcal B,\mathcal C,\mathcal D\subset {\Omega }_{\Lambda }$ be nonempty and decreasing events such that $\mathcal B$ and $\mathcal D$ are independent, then

8 $$\begin{aligned} {\mu }(\mathcal A|\mathcal B\cap \mathcal D)&{}\geqslant {\mu }(\mathcal A|\mathcal B)\geqslant {\mu }(\mathcal A), \end{aligned}$$

9 $$\begin{aligned} {\mu }(\mathcal A\cap \mathcal C|\mathcal B\cap \mathcal D)&{}\geqslant {\mu }(\mathcal A|\mathcal B){\mu }(\mathcal C|\mathcal D). \end{aligned}$$

#### Proof

The first inequality in Eq. (8) is Eq. (9) for $\mathcal C=\Omega _\Lambda$, the second follows from Eq. (7) and ${\mu }(\mathcal A|\mathcal B)={\mu }(\mathcal A\cap \mathcal B)/{\mu }(\mathcal B)$, while Eq. (9) is

$$\begin{aligned} {\mu }(\mathcal A\cap \mathcal C|\mathcal B\cap \mathcal D)=\frac{{\mu }(\mathcal A\cap \mathcal C\cap \mathcal B\cap \mathcal D)}{{\mu }(\mathcal B\cap \mathcal D)}\geqslant \frac{{\mu }(\mathcal A\cap \mathcal B){\mu }(\mathcal C\cap \mathcal D)}{{\mu }(\mathcal B){\mu }(\mathcal D)}={\mu }(\mathcal A|\mathcal B){\mu }(\mathcal C|\mathcal D), \end{aligned}$$

using Eq. (7) in the numerator and independence in the denominator. $\square$

We collectively refer to Eqs. (7)–(9) as *Harris inequality*.

### Directions

Throughout this work we fix a critical update family $\mathcal U$ with difficulty ${\alpha }$. We call a direction $u\in S^1$ *rational* if $u{\mathbb R} \cap {\mathbb Z} ^2\ne \{0\}$. It follows from Definition 1.1 that isolated and semi-isolated stable directions are rational [8, Theorem 1.10]. Therefore, by Definition 1.3 there exists an open semicircle with rational midpoint $u_0$ such that all directions in the semicircle have difficulty at most ${\alpha }$. We may assume without loss of generality that the direction $u_0+\pi /2$ is hard unless $\mathcal U$ is isotropic. It is not difficult to show (see e.g. [8, Lemma 5.3]) that one can find a nonempty set $\mathcal S'$ of rational directions such that:

- all isolated and semi-isolated stable directions are in $\mathcal S'$;
- $$u_0\in \mathcal S'$$;
- for every two consecutive directions *u*, *v* in $\mathcal S'$ either there exists a rule $X\in \mathcal U$ such that $X\subset {\overline{{\mathbb H} }}_{u}\cap {\overline{{\mathbb H} }}_v$ or all directions between *u* and *v* are stable.

We further consider the set ${\widehat{\mathcal S}}=\mathcal S'+\{0,\pi /2,\pi ,3\pi /2\}$ obtained by making $\mathcal S'$ invariant by rotation by $\pi /2$. It is not hard to verify that the three conditions above remain valid when we add directions, so they are still valid for ${\hat{\mathcal S}}$ instead of $\mathcal S'$. We refer to the elements of ${\widehat{\mathcal S}}$ as *quasi-stable directions* or simply *directions*, as they are the only ones of interest to us. We label the elements of ${\widehat{\mathcal S}}=(u_i)_{i\in [4k]}$ clockwise and consider their indices modulo 4*k* (we write [*n*] for $\{0,\dots ,n-1\}$), so that $u_{i+2k}=-u_{i}$ (the inverse being taken in ${\mathbb R} ^2$ and not w.r.t. the angle) is perpendicular to $u_{i+k}$. In figures we take ${\widehat{\mathcal S}}=\frac{\pi }{4}({\mathbb Z} /8{\mathbb Z} )$ and $u_0=(-1,0)$. Further observe that if all $U\in \mathcal U$ are contained in the axes of ${\mathbb Z} ^2$, then we may set ${\widehat{\mathcal S}}=\frac{\pi }{2}({\mathbb Z} /4{\mathbb Z} )$.

For $i\in [4k]$ we introduce ${\rho }_i=\min \{{\rho }>0:\exists x\in {\mathbb Z} ^2,\langle x,u_i\rangle ={\rho }\}$ and ${\lambda }_i=\min \{{\lambda }>0:{\lambda }u_i\in {\mathbb Z} ^2\}$, which are both well-defined, as the directions are rational (in fact ${\rho }_i{\lambda }_i=1$, but we use both notations for transparency).

### Droplets

We next define the geometry of the droplets we use. Recall half planes from Eq. (3).

#### Definition 3.3

(*Droplet*). A *droplet* is a nonempty closed convex polygon of the form

$$\begin{aligned} {\Lambda }(\underline{r})=\bigcap _{i\in [4k]}{\overline{{\mathbb H} }}_{u_i}(r_i) \end{aligned}$$

for some *radii* $\underline{r}\in {\mathbb R} ^{[4k]}$ (see the black regions in Fig. 2). For a sequence of radii $\underline{r}$ we define the *side lengths* $\underline{s}=(s_i)_{i\in [4k]}$ with $s_i$ the length of the side of ${\Lambda }(\underline{r})$ with outer normal $u_i$.

We say that a droplet is *symmetric* if it is of the form $x+{\Lambda }(\underline{r})$ with $2x\in {\mathbb Z} ^2$ and $r_i=r_{i+2k}$ for all $i\in [2k]$. If this is the case, we call *x* the *center* of the droplet.

Note that if all $U\in \mathcal U$ are contained in the axes of ${\mathbb Z} ^2$, then droplets are simply rectangles with sides parallel to the axes.

We write $(\underline{e}_i)_{i\in [4k]}$ for the canonical basis of ${\mathbb R} ^{[4k]}$ and we write $\underline{1}=\sum _{i\in [4k]}\underline{e}_i$, so that ${\Lambda }(r\underline{1})$ is a polygon with inscribed circle of radius *r* and sides perpendicular to ${\widehat{\mathcal S}}$. It is often more convenient to parametrise dimensions of droplets differently. For $i\in [4k]$ we set

10 $$\begin{aligned} \underline{v}_i=\sum _{j=i-k+1}^{i+k-1}\langle u_i,u_j\rangle \underline{e}_j. \end{aligned}$$

This way ${\Lambda }(\underline{r}+\underline{v}_i)$ is obtained from ${\Lambda }(\underline{r})$ by extending the two sides parallel to $u_i$ by 1 in direction $u_i$ and leaving all other side lengths unchanged (see Fig. 2a). Note that if ${\Lambda }(\underline{r})$ is symmetric, then so is ${\Lambda }(\underline{r}+{\lambda }_i\underline{v}_i)$ for $i\in [4k]$.

#### Definition 3.4

(*Tube*). Given $i\in [4k]$, $\underline{r}$ and $l>0$, we define the *tube of length l*, *direction i* *and radii *$\underline{r}$ (see the thickened regions in Fig. 2)

$$\begin{aligned} T(\underline{r},l,i)={\Lambda }(\underline{r}+l\underline{v}_i)\setminus {\Lambda }(\underline{r}). \end{aligned}$$

We often need to consider boundary conditions for our events on droplets and tubes. Given two disjoint finite regions $A,B\subset {\mathbb Z} ^2$ and two configurations ${\eta }\in {\Omega }_A$ and ${\omega }\in {\Omega }_{B}$, we define ${\eta }\cdot {\omega }\in {\Omega }_{A\cup B}$ as

11 $$\begin{aligned} ({\eta }\cdot {\omega })_x={\left\{ \begin{array}{ll} {\eta }_x&{}x\in A,\\ {\omega }_x&{}x\in B. \end{array}\right. } \end{aligned}$$

### Scales

Throughout the work we consider the positive integer constants

$$\begin{aligned} 1/{\varepsilon }\gg 1/{\delta }\gg C\gg W. \end{aligned}$$

Each one is assumed to be large enough depending on $\mathcal U$ and, therefore, ${\widehat{\mathcal S}}$ and ${\alpha }$ (e.g. $W>{\alpha }$), and much larger than any explicit function of the next (e.g. $e^W<C$). These constants are not allowed to depend on *q*. Whenever asymptotic notation is used, its implicit constants are not allowed to depend on the above ones, but only on $\mathcal U$. Also recall Footnote ^1^.

The following are our main scales corresponding to the mesoscopic and internal dynamics:

$$\begin{aligned} \ell ^{\textrm{mes}+}&{}=q^{-C}/\sqrt{{\delta }},&\ell ^{\textrm{mes}}&{}=q^{-C},\\ \ell ^{\textrm{mes}-}&{}=q^{-C}\cdot \sqrt{{\delta }},&\ell ^{\textrm{int}}&{}=C^2\log (1/q)/q^{\alpha }. \end{aligned}$$

### Helping sets

We next introduce various constant-sized sets of infections sufficient to induce growth. As the definitions are quite technical in general, in Fig. 1 we introduce a deliberately complicated example, on which to illustrate them.

#### Helping sets for a line

Recall $(u_i)_{i\in [4k]}$ and $(\lambda _i)_{i\in [4k]}$ from Sect. 3.2 and that for $i\in [4k]$, the direction $u_{i+k}$ is obtained by rotating $u_i$ clockwise by $\pi /2$.

##### Definition 3.5

(*W*-*helping set in direction* $u_i$). Let $i\in [4k]$. A *W*-helping set in direction $u_i$ is any set of *W* consecutive infected sites in ${\overline{{\mathbb H} }}_{u_i}\setminus {\mathbb H} _{u_i}$, that is, a set of the form $x+[W]{\lambda }_{i+k}u_{i+k}$ for some $x\in {\overline{{\mathbb H} }}_{u_i}\setminus {\mathbb H} _{u_i}$.

The relevance of *W*-helping sets in direction $u_i$ is that, since *W* is large enough, $[Z\cup {\mathbb H} _{u_i}]_\mathcal U={\overline{{\mathbb H} }}_{u_i}$ for any direction $u_i$ such that $\alpha (u_i)<\infty$ and *Z* a *W*-helping set in direction $u_i$ (see [8, Lemma 5.2]).

We next define some smaller sets which are sufficient to induce such growth but have the annoying feature that they are not necessarily contained in ${\overline{{\mathbb H} }}_{u_i}$ and do not necessarily induce growth in a simple sequential way like *W*-helping sets in direction $u_i$. Let us note that except in “Appendix A.2” the reader will not lose anything conceptual by thinking that the sets $Z_i$, $u_i$-helping sets and ${\alpha }$-helping sets in direction $u_i$ defined below are simply single infected sites in ${\overline{{\mathbb H} }}_{u_i}\setminus {\mathbb H} _{u_i}$ and the period *Q* is 1.

In words, the set $Z_i$ provided by the following lemma together with ${\mathbb H} _{u_i}$ can infect a semi-sublattice of the first line outside ${\mathbb H} _{u_i}$ and only a finite number of other sites.

##### Lemma 3.6

Let $i\in [4k]$ be such that $0<{\alpha }(u_i)\leqslant {\alpha }$. Then there exists a set $Z_i\subset {\mathbb Z} ^2\setminus {\mathbb H} _{u_i}$ and $x_i\in {\mathbb Z} ^2\setminus \{0\}$ such that

$$\begin{aligned} \langle x_i,u_i\rangle&{}=0,&|Z_i|&{}={\alpha },&\left| \left[ Z_i\cup {\mathbb H} _{u_i}\right] _\mathcal U\setminus {\overline{{\mathbb H} }}_{u_i}\right|&{}<\infty ,&\left[ Z_i\cup {\mathbb H} _{u_i}\right] _\mathcal U&{}\supset x_i{\mathbb N} , \end{aligned}$$

where $${\mathbb N} =\{0,1,\dots \}$$.

##### Proof

Definition 1.3 supplies a set $Z\subset {\mathbb Z} ^2{\setminus }{\mathbb H} _{u_i}$ such that ${\overline{Z}}=[{\mathbb H} _{u_i}\cup Z]_\mathcal U{\setminus }{\mathbb H} _{u_i}$ is infinite and $|Z|=\alpha (u_i)$. Among all possible such *Z*, choose *Z* to minimise $l=\max \{\langle z,u_i\rangle :z\in Z\}$. Yet, $u_i$ is stable, since $\alpha (u_i)\ne 0$ (recall Definition 1.3). Therefore, ${\overline{Z}}\subset {\overline{{\mathbb H} }}_{u_i}(l)\setminus {\mathbb H} _{u_i}$, because $Z\cup {\mathbb H} _{u_i}\subset {\overline{{\mathbb H} }}_{u_i}(l)$ (recall Definition 1.1 and observe that it implies that $[{\overline{{\mathbb H} }}_{u_i}(l)]_\mathcal U={\overline{{\mathbb H} }}_{u_i}(l)$).

Then [7, Lemma 3.3] asserts that $\overline{Z}\cap {\overline{{\mathbb H} }}_{u_i}$ is either finite or contains $x_i{\mathbb N}$ for some $x_i\in {\overline{{\mathbb H} }}_{u_i}\setminus ({\mathbb H} _{u_i}\cup \{0\})$. Assume that $|{\overline{Z}}\setminus {\overline{{\mathbb H} }}_{u_i}|<\infty$, so that $|\overline{Z}\cap {\overline{{\mathbb H} }}_{u_i}|=\infty$, since $|{\overline{Z}}|=\infty$. Then we conclude by setting $Z_i$ equal to the union of *Z* with $\alpha -\alpha (u_i)$ arbitrarily chosen elements of $\overline{Z}{\setminus } Z$, so that $\overline{Z_i}={\overline{Z}}$.

Assume for a contradiction that, on the contrary, $|\overline{Z}{\setminus }{\overline{{\mathbb H} }}_{u_i}|=\infty$. Set $Z'=(Z-\rho _iu_i){\setminus }{\mathbb H} _u$ (i.e. shift *Z* one line closer to ${\mathbb H} _{u_i}$) and observe that $\overline{Z'}\supset ({\overline{Z}}\setminus {\overline{{\mathbb H} }}_{u_i}-\rho _iu_i)$ is still infinite. Therefore, by Definition 1.3$\alpha (u_i)\leqslant |Z'|\leqslant |Z|=\alpha (u_i)$. This contradicts our choice of *Z* minimising *l*. $\square$

In the example of Fig. 1 the $u_3$ direction admits a set $Z_3$ of cardinality 3 such that $[Z_3\cup {\mathbb H} _{u_3}]_\mathcal U$ only contains every second site of the line ${\overline{{\mathbb H} }}_{u_i}\setminus {\mathbb H} _{u_i}$, while at least 4 sites are needed to infect the entire line. Thus, in order to efficiently infect ${\overline{{\mathbb H} }}_{u_3}\setminus {\mathbb H} _{u_3}$, assuming ${\mathbb H} _{u_3}$ is infected, we may use two translates of $Z_3$ with different parity. This technicality is reflected in the next definition.

##### Definition 3.7

($u_i$-*helping set*). For all $i\in [4k]$ such that $0<{\alpha }(u_i)\leqslant {\alpha }$ fix a choice of $Z_i$ and $x_i$ as in Lemma 3.6 in such a way that the *period*

$$\begin{aligned} Q=\frac{\Vert x_i\Vert }{{\lambda }_{i+k}} \end{aligned}$$

is independent of *i* and sufficiently large so that the diameter of $\{0\}\cup Z_i$ is much smaller than *Q*. A $u_i$-*helping set* is a set of the form

12 $$\begin{aligned} \bigcup _{j\in \left[ Q\right] }\left( Z_i+j{\lambda }_{i+k}u_{i+k}+k_jx_i\right) , \end{aligned}$$

for some integers $k_j$. For $i\in [4k]$ with ${\alpha }(u_i)=0$, we define $u_i$-helping sets to be empty. For $i\in [4k]$ with $\alpha (u_i)>\alpha$ there are no $u_i$-helping sets.

Note that by Lemma 3.6 a $u_i$-helping set *Z* is sufficient to infect a half-line, but since that contains a *W*-helping set in direction $u_i$, we have $[Z\cup {\mathbb H} _{u_i}]_\mathcal U\supset {\overline{{\mathbb H} }}_{u_i}$.

We further incorporate the artificial symmetrisation alluded to in Remark 2.1 in the next definition.

##### Definition 3.8

(${\alpha }$-*helping set in direction* $u_i$). Let $i\in [4k]$.

- If ${\alpha }(u_i)\leqslant {\alpha }$ and ${\alpha }(u_{i+2k})\leqslant {\alpha }$, then a ${\alpha }$-*helping set in direction *$u_i$ is a set of the form $H\cup H'$ with *H* a $u_i$-helping set and $-H'=\{-h:h\in H'\}$ a $u_{i+2k}$-helping set.
- If ${\alpha }(u_i)\leqslant {\alpha }$ and ${\alpha }(u_{i+2k})>{\alpha }$, then a ${\alpha }$-*helping set in direction *$u_i$ is a $u_i$-helping set.
- If ${\alpha }<{\alpha }(u_i)\leqslant \infty$, there are no ${\alpha }$-*helping sets in direction *$u_i$.

If ${\alpha }(u_i)<\infty$, any set which is either a *W*-helping set in direction $u_i$ or a ${\alpha }$-helping set in direction $u_i$ is called *helping set in direction *$u_i$. If ${\alpha }(u_i)=\infty$, there are no *helping sets in direction *$u_i$.

In the example of Fig. 1$u_0$ and $u_2$ are both of difficulty ${\alpha }=3$, so ${\alpha }$-helping sets in direction $u_0$ correspond to $(z_1+\{(0,0),(2,0),(3,0)\})\cup (z_2+\{(0,0),(-2,1),(0,2)\})$ for some $(z_1,z_2)\in (\{0\}\times {\mathbb Z} )^2$. The set $z_2+\{(0,0),(-2,1),(0,2)\}$ is not a $u_0$-helping set, but we include it in ${\alpha }$-helping sets in direction $u_0$. We do so, in order for ${\alpha }$-helping sets in direction $u_0$ and $u_2$ to be symmetric. Namely, they satisfy that *Z* is a $\alpha$-helping set in direction $u_0$ if and only if $-Z$ is a $\alpha$-helping set in direction $u_2$.

#### Helping sets for a segment

For this section we fix a direction $u_{i}\in {\widehat{\mathcal S}}$ with ${\alpha }(u_i)<\infty$ and a discrete segment *S* perpendicular to $u_i$ of the form

13 $$\begin{aligned} \left\{ x\in {\mathbb Z} ^2: \langle x,u_i\rangle =0,\langle x,u_{i+k}\rangle /{\lambda }_{i+k}\in [0,a]\right\} \end{aligned}$$

for some integer $a\geqslant W$. The direction $u_i$ is kept implicit in the notation, so it may be useful to view *S* as having an orientation.

##### Definition 3.9

For $d\geqslant 0$, we denote by $\mathcal H^W_d(S)$ the event that there is an infected *W*-helping set in direction $u_i$ in *S* at distance at least *d* from its endpoints:

$$\begin{aligned}{} & {} \mathcal H^W_d(S)=\big \{\eta \in \Omega :\exists x\in {\mathbb Z} \cap [d/\lambda _{i+k},a-(W-1)-d/\lambda _{i+k}],\\{} & {} \quad \eta _{(x+[W])\lambda _{i+k}u_{i+k}}=\textbf{0}\big \}. \end{aligned}$$

We write $$\mathcal H^W(S)=\mathcal H^W_0(S)$$.

For helping sets the definition is more technical, since they are not included in *S*. We therefore require that they are close to *S* and at some distance from its endpoints.

##### Definition 3.10

For $d\geqslant 0$, we denote by $\mathcal H_{d}(S)\subset \Omega$ the event such that $\eta \in \mathcal H_d(S)$ if there exists *Z* a helping set in direction $u_i$ such that for all $z\in Z$, we have $\eta _z=0$,

14 $$\begin{aligned} \langle z,u_i\rangle&{}\in [0,Q],&\langle z,u_{i+k}\rangle&{}\in \left[ d,a{\lambda }_{i+k}-d\right] . \end{aligned}$$

Given a domain ${\Lambda }\supset S$ and a boundary condition ${\omega }\in {\Omega }_{{\mathbb Z} ^2\setminus {\Lambda }}$ we define $\mathcal H^{{\omega }}_d(S)=\{{\eta }\in {\Omega }_{{\Lambda }}:{\omega }\cdot {\eta }\in \mathcal H_d(S)\}$. We write $\mathcal H^{\omega }(S)=\mathcal H^{\omega }_0(S)$ and $\mathcal H(S)=\mathcal H_0(S)$.

Note that in view of Definition 3.8, if $\alpha (u_i)<\infty$, then $\mathcal H^{\omega }(S)\supset \mathcal H^W(S)$ for any ${\omega }$ with equality if $\alpha (u_i)>\alpha$. The next observation bounds the probability of the above events.

##### Observation 3.11

(Helping set probability) For any $\Lambda \supset S$ and ${\omega }\in \Omega _{{\mathbb Z} ^2\setminus \Lambda }$ we have: if ${\alpha }(u_i)<\infty$, then

$$\begin{aligned} {\mu }\left( \mathcal H^{\omega }(S)\right) \geqslant {\mu }\left( \mathcal H^W(S)\right) \geqslant 1-\left( 1-q^W\right) ^{\lfloor |S|/W\rfloor }\geqslant \max \left( q^W,1-e^{-q^{2W}|S|}\right) ; \end{aligned}$$

if $${\alpha }(u_i)\leqslant {\alpha }$$, then

$$\begin{aligned} {\mu }(\mathcal H(S))\geqslant \left( 1-(1-q^{\alpha })^{{\Omega }(|S|)}\right) ^{O(1)}\geqslant \left( 1-e^{-q^{\alpha }|S|/O(1)}\right) ^{O(1)}. \end{aligned}$$

##### Proof

Assume $\alpha (u_i)<\infty$. As already observed, by Definitions 3.8–3.10, $\mathcal H^{\omega }(S)\supset \mathcal H^W(S)$, as *W*-helping sets in direction $u_i$ are helping sets in direction $u_i$. For the second inequality follows by dividing *S* into disjoint groups of *W* consecutive sites (each of which is a *W*-helping set in direction $u_i$). The final inequality follows since $|S|\geqslant W$ and $(1-q^W)^{1/W}\leqslant e^{-q^W/(2W)}\leqslant e^{-q^{2W}}$.

The case $\alpha (u_i)\leqslant \alpha$ is treated similarly. Indeed, in order for $\mathcal H(S)$ to occur, we need to find each of the $Q=O(1)$ pieces of a $u_i$-helping set in Eq. (12), each of which has cardinality $\alpha$. We direct the reader to [7, Lemma 4.2] for more details. $\square$

### Constrained Poincaré inequalities

We next define the (constrained) Poincaré constants of various regions. For ${\Lambda }\subset {\mathbb Z} ^2$, $\eta ,\omega \in {\Omega }$ (or possibly $\eta$ defined on a set including $\Lambda$ and $\omega$ on a set including ${\mathbb Z} ^2\setminus \Lambda$) and $x\in {\mathbb Z} ^2$, we denote by $c_x^{{\Lambda },{\omega }}({\eta })=c_x({\eta }_{\Lambda }\cdot {\omega }_{{\mathbb Z} ^2\setminus {\Lambda }})$ (recall Eqs. (1) and (11)) the constraint at *x* in $\Lambda$ with boundary condition $\omega$. Given a finite ${\Lambda }\subset {\mathbb Z} ^2$ and a nonempty event $\mathcal S\mathcal G^\textbf{1}({\Lambda })\subset {\Omega }_{\Lambda }$, let ${\gamma }({\Lambda })$ be the smallest constant ${\gamma }\in [1,\infty ]$ such that the inequality

15 $$\begin{aligned} {\text {Var}}_{{\Lambda }}\left( f|\mathcal S\mathcal G^\textbf{1}({\Lambda })\right) \leqslant {\gamma }\sum _{x\in {\Lambda }}{\mu }_{\Lambda }\left( c_x^{\Lambda ,\textbf{1}}{\text {Var}}_x(f)\right) \end{aligned}$$

holds for all $f:{\Omega }\rightarrow {\mathbb R}$. Here we recall from Sect. 1.1 that ${\mu }$ denotes both the product Bernoulli probability distribution with parameter *q* and the expectation with respect to it. Moreover, for any function $\phi :{\Omega }\rightarrow {\mathbb R}$, ${\mu }_{\Lambda }(\phi )={\mu }(\phi (\eta )|\eta _{{\mathbb Z} ^2\setminus {\Lambda }})$ is the average on the configuration $\eta$ of law ${\mu }$ in ${\Lambda }$, conditionally on its state in ${\mathbb Z} ^2\setminus {\Lambda }$. Thus, ${\mu }_{\Lambda }(\phi )$ is a function on ${\Omega }_{{\mathbb Z} ^2\setminus {\Lambda }}$. Similarly, ${\text {Var}}_x(f)={\mu }(f^2(\eta )|\eta _{{\mathbb Z} ^2{\setminus } \{x\}})-{\mu }^2(f(\eta )|\eta _{{\mathbb Z} ^2{\setminus } \{x\}})$ and

$$\begin{aligned}{} & {} {\text {Var}}_{\Lambda }\left( f|\mathcal S\mathcal G^\textbf{1}({\Lambda })\right) ={\mu }\left( \left. f^2(\eta )\right| {\eta }_{\Lambda }\in \mathcal S\mathcal G^{\textbf{1}}(\Lambda ),\eta _{{\mathbb Z} ^2\setminus {\Lambda }}\right) \\{} & {} \quad -{\mu }^2\left( f(\eta )|{\eta }_{\Lambda }\in \mathcal S\mathcal G^{\textbf{1}}(\Lambda ),\eta _{{\mathbb Z} ^2\setminus {\Lambda }}\right) . \end{aligned}$$

#### Remark 3.12

It is important to note that in the r.h.s. of Eq. (15) we average w.r.t. ${\mu }_{\Lambda }$ and not ${\mu }_{\Lambda }(\cdot |\mathcal S\mathcal G^\textbf{1}({\Lambda }))$ (the latter would correspond to the usual definition of Poincaré constant, from which we deviate). In this respect Eq. (15) follows [22, Eq. (12)] and differs from [24, Eq. (4.5)]. Although this nuance is not important most of the time, this choice is crucial for the proof of Theorem 8.5 below.

### Boundary conditions, translation invariance, monotonicity

Let us make a few conventions in order to lighten notation throughout the paper. As we already witnessed in Sect. 3.5, it is often the case that much of the boundary condition is actually irrelevant for the occurrence of the event. For instance, in Definition 3.10, $\mathcal H^{\omega }(S)$ only depends on the restriction of ${\omega }$ to a finite-range neighbourhood of the segment *S*. Moreover, even the state in ${\omega }$ of sites close to *S*, but in ${\mathbb H} _{u_i}$ is of no importance. Such occasions arise frequently, so, by abuse, we allow ourselves to specify a boundary condition on any region containing the sites whose state actually matters for the occurrence of the event.

We also need the following natural notion of translation invariance.

#### Definition 3.13

(*Translation invariance*). Let $A\subset {\mathbb R} ^2$. Consider a collection of events $\mathcal E^{\omega }(A+x)$ for $x\in {\mathbb Z} ^2$ and ${\omega }\in {\Omega }_{{\mathbb Z} ^2{\setminus }(A+x)}$. We say that $\mathcal E(A)$ is *translation invariant*, if for all $\eta \in {\Omega }_A$, ${\omega }\in {\Omega }_{{\mathbb Z} ^2{\setminus } A}$ and $x\in {\mathbb Z} ^2$ we have

$$\begin{aligned} \eta \in \mathcal E^{\omega }(A)\Leftrightarrow \eta _{\cdot -x}\in \mathcal E^{{\omega }_{\cdot -x}}(A+x). \end{aligned}$$

Similarly, we say that $\mathcal E^{\omega }(A)$ is *translation invariant*, if the above holds for a fixed ${\omega }\in {\Omega }_{{\mathbb Z} ^2\setminus A}$.

We extend the events $\mathcal H_d(S)$, $\mathcal H_d^{\omega }(S)$, $\mathcal H_d^W(S)$ from Definitions 3.9 and 3.10 in a translation invariant way. Similarly, $\mathcal T$ and $\mathcal S\mathcal T$ events for tubes defined in Sect. 4.1 below and $\mathcal S\mathcal G$ events for droplets defined throughout the paper are translation invariant. Therefore, we sometimes only define them for a fixed region, as we did in Sect. 3.5.2, but systematically extended them in a translation invariant way to all translates of this region.

We also use the occasion to point out that, just like the event $\mathcal H_d^{\omega }(S)$, all our $\mathcal T$, $\mathcal S\mathcal T$ and $\mathcal S\mathcal G$ events are decreasing in both the configuration and the boundary condition, so that we are able to apply Sect. 3.1 as needed.

## One-Directional Extensions

In this section we define our crucial one-directional CBSEP-extension and East-extension techniques (recall Sect. 2.3).

### Traversability

We first need the following traversability $\mathcal T$ and symmetric traversability $\mathcal S\mathcal T$ events for tubes (recall Definition 3.4) requiring infected helping sets (recall Sect. 3.5.2) to appear for each of the segments composing the tube. The definition is illustrated in Fig. 2. Recall the constant *C* from Sect. 3.4

#### Definition 4.1

(*Traversability*). Fix a tube $T=T(\underline{r},l,i)$. Assume that $i\in [4k]$ is such that ${\alpha }(u_j)<\infty$ for all $j\in (i-k,i+k)$. For $m\geqslant 0$ and $j\in (i-k,i+k)$ write $S_{j,m}={\mathbb Z} ^2\cap {\Lambda }(\underline{r}+m\underline{v}_i+{\rho }_j\underline{e}_j)\setminus {\Lambda }(\underline{r}+m\underline{v}_i)$. Note that $S_{j,m}$ is a discrete line segment perpendicular to $u_j$ of length $s_j-O(1)$ (recall from Definition 3.3 that $\underline{s}$ is the sequence of side lengths of $\Lambda (\underline{r})$). For ${\omega }\in {\Omega }_{{\mathbb Z} ^2\setminus {\Lambda }(\underline{r}+l\underline{v}_i)}$ we denote by

$$\begin{aligned} \mathcal T^{\omega }_d(T)=\bigcap _{j,m:\varnothing \ne S_{j,m}\subset T}\mathcal H^{\omega }_{C^2+d}\left( S_{j,m}\right) \end{aligned}$$

the event that *T* is $({\omega },d)$-*traversable*. We set $\mathcal T^{\omega }(T)=\mathcal T^{\omega }_0(T)$.

If moreover $\alpha (u_i)<\infty$ for all $i\in [4k]$, that is, $\mathcal U$ has a finite number of stable directions, we denote by

$$\begin{aligned} \mathcal S\mathcal T^{\omega }_d(T)=\mathcal T_d^{\omega }(T)\cap \bigcap _{j:{\alpha }(u_j)\leqslant \alpha <{\alpha }(u_{j+2k}))}\bigcap _{m:\varnothing \ne S_{j,m}\subset T}\mathcal H^W_{C^2+d}\left( S_{j,m}\right) \end{aligned}$$

the event that *T* is $({\omega },d)$-*symmetrically traversable*.

Thus, if all side lengths of ${\Lambda }(\underline{r})$ are larger than $C^2+d$ by a large enough constant, the event $\mathcal T^{\omega }_d(T(\underline{r},s,i))$ decomposes each of the hatched parallelograms in Fig. 2a into line segments parallel to its side that is not parallel to $u_i$. A helping set is required for each of these segments in the direction perpendicular to them which has positive scalar product with $u_i$. The last boundedly many segments may also use the boundary condition ${\omega }$, but it is irrelevant for the remaining ones, since it is far enough from them.

For symmetric traversability, we rather require *W*-helping sets for opposites of hard directions (recall from Definition 3.8 that if the direction itself is hard, helping sets are simply *W*-helping sets). In particular, if none of the directions $u_j$ for $j\in [4k]\setminus \{i+k,i-k\}$ is hard (implying that $\mathcal U$ is unrooted), we have $\mathcal S\mathcal T^{\omega }_d(T(\underline{r},l,i))=\mathcal T^{\omega }_d(T(\underline{r},l,i))$. The reason for the name “symmetric traversability” is that if $\mathcal U$ has a finite number of stable directions and ${\Lambda }(\underline{r})$ is a symmetric droplet (recall Sect. 3.3), then, for any $l>0$, $i\in [4k]$, ${\omega }\in {\Omega }_{{\mathbb Z} ^2\setminus T(\underline{r},l,i)}$ and ${\eta }\in {\Omega }_{T(\underline{r},l,i)}$, we have

16 $$\begin{aligned} \eta \in \mathcal S\mathcal T^{\omega }_d(T(\underline{r},l,i))\Leftrightarrow \eta '\in \mathcal S\mathcal T^{{\omega }'}_d(T(\underline{r},l,i+2k)), \end{aligned}$$

denoting by ${\omega }'\in {\Omega }_{{\mathbb Z} ^2\setminus T(\underline{r},l,i+2k)}$ the boundary condition obtained by rotating ${\omega }$ by $\pi$ around the center of ${\Lambda }(\underline{r})$ and similarly for $\eta '$. To see this, recall from Sect. 3.5.2 that $\mathcal H^{\omega }(S)\supset \mathcal H^W(S)$ with equality when $\alpha (u_i)>\alpha$ and note that the same symmetry as in Eq. (16) holds at the level of the segment $S_{j,m}$ and its symmetric one, $S'_{j+2k,m}={\mathbb Z} ^2\cap {\Lambda }(\underline{r}+m\underline{v}_{i+2k}+{\rho }_{j+2k}\underline{e}_{j+2k})\setminus {\Lambda }(\underline{r}+m\underline{v}_{i+2k})$:

$$\begin{aligned} \eta \in {\left\{ \begin{array}{ll}\mathcal H^{\omega }_{C^2+d}(S_{j,m})&{}\alpha (u_{j+2k})\leqslant \alpha \\ \mathcal H^W_{C^2+d}(S_{j,m})&{}\alpha (u_{j+2k})>\alpha \end{array}\right. }\Leftrightarrow \eta '\in {\left\{ \begin{array}{ll}\mathcal H^{{\omega }'}_{C^2+d}(S'_{j+2k,m})&{} \alpha (u_j)\leqslant \alpha \\ \mathcal H^W_{C^2+d}(S'_{j+2k,m})&{}\alpha (u_j)>\alpha ,\end{array}\right. } \end{aligned}$$

all four cases following directly from Definitions 3.8–3.10.

We next state a simple observation which is used frequently to modify boundary conditions as we like at little cost.

#### Lemma 4.2

(Changing boundary conditions). Let ${\Lambda }(\underline{r})$ be a droplet, $l>0$ be a multiple of ${\lambda }_i$ and $i\in [4k]$. Assume that for any $j\in [4k]\setminus \{i-k,i+k\}$ the side length $s_j$ of ${\Lambda }(\underline{r})$ satisfies $s_j\geqslant C^3$. Set $T=T(\underline{r},l,i)$. Then there exists a decreasing event $\mathcal W(T)\subset {\Omega }_T$ such that ${\mu }(\mathcal W(T))\geqslant q^{O(W)}$ for any ${\omega }\in {\Omega }_{{\mathbb Z} ^2{\setminus } T}$ and $\eta \in \mathcal W(T)$ we have

$$\begin{aligned} {\eta }\in \mathcal T^{\omega }(T)\Leftrightarrow {\eta }\in \mathcal T^\textbf{1}(T). \end{aligned}$$

Moreover, ${\mu }(\mathcal T^{\omega }(T))=q^{-O(W)}{\mu }(\mathcal T^\textbf{1}(T))$ for all ${\omega }\in {\Omega }_{{\mathbb Z} ^2\setminus T}$. The same holds with $\mathcal S\mathcal T$ instead of $\mathcal T$.

#### Proof

Recall the segments $S_{j,m}$ from Definition 4.1. Let $\mathcal W(T)$ be the intersection of $\mathcal H^W_{C^2}(S_{j,m})$ for the largest sufficiently large but fixed number of values of *m* for each $j\in (i-k,i+k)$, such that $\varnothing \ne S_{j,m}\subset T$. By Observation 3.11${\mu }(\mathcal W(T))\geqslant q^{O(W)}$. Moreover, the boundary condition is irrelevant for the remaining segments, so $\mathcal W(T)$ is indeed as desired. Finally, by Eq. (7) we have

$$\begin{aligned} {\mu }\left( \mathcal T^\textbf{1}(T)\right)&{}\leqslant {\mu }\left( \mathcal T^{\omega }(T)\right) \leqslant \frac{{\mu }(\mathcal W(T)\cap \mathcal T^{\omega }(T))}{{\mu }(\mathcal W(T))}\\&{}\leqslant q^{-O(W)}{\mu }\left( \mathcal W(T)\cap \mathcal T^\textbf{1}(T)\right) \leqslant q^{-O(W)}{\mu }\left( \mathcal T^\textbf{1}(T)\right) . \end{aligned}$$

$$\square $$

Another convenient property allowing us to decompose a long tube into smaller ones is the following.

#### Lemma 4.3

(Decomposing tubes). Let $T=T(\underline{r},l,i)$ be a tube, ${\omega }\in {\Omega }_{{\mathbb Z} ^2\setminus T}$ be a boundary condition and $s\in [0,l]$ be a multiple of ${\lambda }_i$. Set $T_1=T(\underline{r},s,i)$ and $T_2=su_i+T(\underline{r},l-s,i)$. Then

$$\begin{aligned} {\eta }\in \mathcal T^{\omega }(T(\underline{r},l,i))\Leftrightarrow \left( {\eta }_{T_2}\in \mathcal T^{\omega }(T_2)\text { and }{\eta }_{T_1}\in \mathcal T^{{\eta }_{T_2}\cdot {\omega }}(T_1)\right) \end{aligned}$$

and the same holds for $\mathcal S\mathcal T$ instead of $\mathcal T$.

#### Proof

This follows immediately from Definition 4.1, since for each of the segments $S_{j,m}$ in Definition 4.1 either $S_{j,m}\subset T_1$ or $S_{j,m}\cap T_1=\varnothing$ and similarly for $T_2$ (see Fig. 2a). $\square$

### East-extension

We start with the East-extension (see Fig. 2a), which is simpler to state.

#### Definition 4.4

(*East-extension*). Fix $i\in [4k]$, a droplet ${\Lambda }(\underline{r})$, a multiple $l>0$ of ${\lambda }_i$ and an event $\mathcal S\mathcal G^\textbf{1}({\Lambda }(\underline{r}))\subset {\Omega }_{{\Lambda }(\underline{r})}$. Assume that ${\alpha }(u_j)<\infty$ for all $j\in (i-k,i+k)$. We use the expression “*we East-extend *${\Lambda }(\underline{r})$ *by l* *in direction* $u_i$” to state that, for all $s\in (0,l]$ multiple of ${\lambda }_i$ and ${\omega }\in {\Omega }_{{\mathbb Z} ^2\setminus {\Lambda }(\underline{r}+s\underline{v}_i)}$, we define the event $\mathcal S\mathcal G^{\omega }({\Lambda }(\underline{r}+s\underline{v}_i))\subset {\Omega }_{{\Lambda }(\underline{r}+s\underline{v}_i)}$ to occur for ${\eta }\in {\Omega }_{{\Lambda }(\underline{r}+s\underline{v}_i)}$ if

$$\begin{aligned} {\eta }_{{\Lambda }(\underline{r})}\in \mathcal S\mathcal G^\textbf{1}({\Lambda }(\underline{r}))\quad \text {and}\quad {\eta }_{T(\underline{r},s,i)}\in \mathcal T^{{\omega }}(T(\underline{r},s,i)). \end{aligned}$$

In other words, given the event $\mathcal S\mathcal G^\textbf{1}$ for the droplet ${\Lambda }(\underline{r})$, we define the event $\mathcal S\mathcal G^{\omega }$ (in particular for ${\omega }=\textbf{1}$, but not only) for the larger droplet ${\Lambda }(\underline{r}+l\underline{v}_i)={\Lambda }(\underline{r})\sqcup T(\underline{r},l,i)$. The event obtained on the larger droplet requires for the smaller one to be $\textbf{1}$-super good (SG) and for the remaining tube to be ${\omega }$-traversable (recall Definition 4.1). Note that these two events are independent. Further observe that if $\mathcal S\mathcal G^\textbf{1}({\Lambda }(\underline{r}))$ is translation invariant (recall Definition 3.13), then so is $\mathcal S\mathcal G({\Lambda }(\underline{r}+s\underline{v}_i))$ for any $s\in (0,l]$ multiple of ${\lambda }_i$, defined by East-extending ${\Lambda }(\underline{r})$ by *l* in direction $u_i$. To get a grasp on Definition 4.4, let us note the following fact, even though it is not used directly in the proof of Theorem 1.

#### Lemma 4.5

(East-extension ergodicity). Let $i\in [4k]$, ${\Lambda }(\underline{r})$ be a droplet, *l* be a multiple of ${\lambda }_i$ and $\mathcal S\mathcal G^\textbf{1}({\Lambda }(\underline{r}))\subset {\Omega }_{{\Lambda }(\underline{r})}$ be an event. Assume that ${\alpha }(u_j)<\infty$ for all $j\in (i-k,i+k)$. Further assume that $\eta \in \mathcal S\mathcal G^\textbf{1}({\Lambda }(\underline{r}))$ implies that the $\mathcal U$-KCM with initial condition ${\eta }\cdot \textbf{1}_{{\mathbb Z} ^2\setminus {\Lambda }(\underline{r})}$ can entirely infect ${\Lambda }(\underline{r})$. If we East-extend ${\Lambda }(\underline{r})$ by *l* in direction $u_i$, then for any ${\omega }\in {\Omega }_{{\mathbb Z} ^2\setminus {\Lambda }(\underline{r}+l\underline{v}_i)}$ and $\eta \in \mathcal S\mathcal G^{\omega }({\Lambda }(\underline{r}+l\underline{v}_i))$ the $\mathcal U$-KCM with initial condition ${\omega }\cdot \eta$ can entirely infect ${\Lambda }(\underline{r}+l\underline{v}_i)$.

#### Proof

The proof is rather standard, so we only sketch the reasoning. Let $\eta \in \mathcal S\mathcal G^{\omega }({\Lambda }(\underline{r}+l\underline{v}_i))$. Since $\eta _{{\Lambda }(\underline{r})}\in \mathcal S\mathcal G^\textbf{1}({\Lambda }(\underline{r}))$ by Definition 4.4, by hypothesis we can completely infect ${\Lambda }(\underline{r})$, starting from ${\omega }\cdot {\eta }$. We next proceed by induction on $s\in [0,l]$ to show that we can infect ${\Lambda }(\underline{r}+s\underline{v}_i)$. When a new site in ${\mathbb Z} ^2$ is added to this set, as we increase *s*, we actually add to it an entire segment $S_{j,m}$ as in Definition 4.1 (at most one *m* for each $j\in (i-k,i+k)$). Since $T(\underline{r},l,i)$ is $({\omega },0)$-traversable, by Definition 3.10 and 4.1, there is a helping set (in direction $u_j$) for this segment. As noted in Sect. 3.5.1, helping sets in direction $u_j$ together with the half-plane ${\mathbb H} _{u_j}$ infect the entire line ${\overline{{\mathbb H} }}_{u_j}\setminus {\mathbb H} _{u_j}$ on the boundary of the half-plane. Since the helping set in our setting is only next to a finite fully infected droplet ${\Lambda }(\underline{r}+s\underline{v}_i)$, infection spreads along its edge until it reaches a bounded distance from the corners (see [7, Lemma 3.4]). However, by our choice of ${\hat{\mathcal S}}$ (recall Sect. 3.2), for each $j\in [4k]$ there is a rule $X\in \mathcal U$ such that $X\subset {\overline{{\mathbb H} }}_{u_j}\cap {\overline{{\mathbb H} }}_{u_{j+1}}$. Using this rule, we can infect even the remaining sites to fill up the corner between directions $u_j$ and $u_{j+1}$ of the droplet ${\Lambda }(\underline{r}+s'\underline{v}_i)$ with $s'>s$ minimal such that ${\Lambda }(\underline{r}+s'\underline{v}_i)\setminus {\Lambda }(\underline{r}+s\underline{v}_i)\ne \varnothing$ (see [8, Lemma 5.5 and Fig. 6]). $\square$

We next state a recursive bound on the Poincaré constant ${\gamma }$ introduced in Sect. 3.6 reflecting the recursive definition of SG events in an East-extension. In rough terms, it states that in order to relax on the larger volume, we need to be able to relax on the smaller one and additionally pay the cost of creating logarithmically many copies of it shifted by exponentially growing offsets, conditionally on the presence of the original droplet. We further need to account for the cost of microscopic dynamics (see the $e^{\log ^2(1/q)}$ term below), but its contribution is unimportant. Recall $\ell ^{\textrm{mes}+}$ from Sect. 3.4.

#### Proposition 4.6

(East-extension relaxation). Let $i\in [4k]$ be such that for all $j\in (i-k,i+k)$ we have ${\alpha }(u_j)<\infty$. Let ${\Lambda }(\underline{r})$ be a droplet with $\underline{r}=q^{-O(C)}$ and side lengths at least $C^3$. Let $l\in (0,\ell ^{\textrm{mes}+}]$ be a multiple of ${\lambda }_i$. Define $d_m={\lambda }_i\lfloor (3/2)^{m}\rfloor$ for $m\in [1,M)$ and $M=\min \{m:{\lambda }_i(3/2)^m\geqslant l\}$. Let $d_M=l$, ${\Lambda }^m={\Lambda }(\underline{r}+d_m\underline{v}_i)$ and $s_{m-1}=d_{m}-d_{m-1}$ for $m\in [2,M]$.

Let $\mathcal S\mathcal G^\textbf{1}({\Lambda }(\underline{r}))$ be a nonempty translation invariant decreasing event. Assume that we East-extend ${\Lambda }(\underline{r})$ by *l* in direction $u_i$. Then $\mathcal S\mathcal G^\textbf{1}({\Lambda }(\underline{r}+l\underline{v}_i))$ is also nonempty, translation invariant, decreasing and satisfies

$$\begin{aligned} {\gamma }({\Lambda }(\underline{r}+l\underline{v}_i))\leqslant \max \left( {\gamma }({\Lambda }(\underline{r})),{\mu }^{-1}\left( \mathcal S\mathcal G^\textbf{1}({\Lambda }(\underline{r}))\right) \right) e^{O(C^2)\log ^2(1/q)}\prod _{m=1}^{M-1}a_m, \end{aligned}$$

with

17 $$\begin{aligned} a_{m}={\mu }^{-1}\left( \left. \mathcal S\mathcal G^\textbf{1}\left( {\Lambda }^m+s_mu_i\right) \right| \mathcal S\mathcal G^\textbf{1}({\Lambda }^m)\right) . \end{aligned}$$

The proof is left to “Appendix A.3”.

### CBSEP-extension

We next turn our attention to CBSEP-extensions (see Fig. 2b). The definition differs from Definition 4.4 (cf. Fig. 2a) in three ways. Firstly, we allow the smaller SG droplet to be anywhere inside the larger one (the exact position is specified by the offset below). Secondly, we ask for traversability on both sides of the smaller droplet in the direction away from it (so that infection can spread, starting from it), rather than just on one side. Thirdly, we require our tubes to be symmetrically traversable, instead of traversable. This makes the position of the small SG droplet roughly uniform.

#### Definition 4.7

(*CBSEP-extension*). Assume that $\mathcal U$ has a finite number of stable directions (equivalently, $\alpha (u_j)<\infty$ for all $j\in [4k]$). Fix $i\in [4k]$, a droplet ${\Lambda }(\underline{r})$ and a multiple *l* of ${\lambda }_i$. Let $\mathcal S\mathcal G^\textbf{1}({\Lambda }(\underline{r}))$ be a translation invariant event. We use the expression “*we CBSEP-extend *${\Lambda }(\underline{r})$ *by* *l* *in direction* $u_i$” to state that, for all $s\in (0,l]$ multiple of ${\lambda }_i$ and ${\omega }\in {\Omega }_{{\mathbb Z} ^2\setminus {\Lambda }(\underline{r}+s\underline{v}_i)}$, we define the event $\mathcal S\mathcal G^{\omega }({\Lambda }(\underline{r}+s\underline{v}_i))\subset {\Omega }_{{\Lambda }(\underline{r}+s\underline{v}_i)}$ as follows.

For *offsets* $x\in [0,s]$ divisible by ${\lambda }_i$ we define ${\eta }\in \mathcal S\mathcal G_x^{\omega }({\Lambda }(\underline{r}+s\underline{v}_i))$ if the following all hold:

$$\begin{aligned} \eta _{T(\underline{r},s-x,i)+xu_i}&{}\in \mathcal S\mathcal T^{{\omega }}(T(\underline{r},s-x,i)+xu_i);\\ {\eta }_{{\Lambda }(\underline{r})+xu_i}&{}\in \mathcal S\mathcal G^\textbf{1}({\Lambda }(\underline{r})+xu_i);\\ \eta _{T(\underline{r},x,i+2k)+xu_i}&{}\in \mathcal S\mathcal T^{{\omega }}(T(\underline{r},x,i+2k)+xu_i). \end{aligned}$$

We then set $\mathcal S\mathcal G^{\omega }({\Lambda }(\underline{r}+s\underline{v}_i))=\bigcup _x\mathcal S\mathcal G^{\omega }_x({\Lambda }(\underline{r}+s\underline{v}_i))$.

Note that CBSEP-extending in direction $u_i$ gives the same result as CBSEP-extending in direction $u_{i+2k}$. We further reassure the reader that, in applications Definitions 4.4 and 4.7, are not used simultaneously for the same droplet ${\Lambda }(\underline{r})$, so no ambiguity arises as to whether $\mathcal S\mathcal G^{\omega }({\Lambda }(\underline{r}+l\underline{v}_i))$ is obtained by CBSEP-extension or East-extension. However, as it is clear from Table Table 2b, it is sometimes necessary to CBSEP-extend a droplet itself obtained by East-extending an even smaller one. But for the time being, let us focus on a single CBSEP-extension.

The following analogue of Lemma 4.5 holds for CBSEP-extension, which is also not used directly in the proof of Theorem 1.

#### Lemma 4.8

(CBSEP-extension ergodicity). Assume that $\mathcal U$ has a finite number of stable directions. Let $i\in [4k]$, ${\Lambda }(\underline{r})$ be a droplet and *l* be a multiple of ${\lambda }_i$. Let $\mathcal S\mathcal G^\textbf{1}({\Lambda }(\underline{r}))\subset {\Omega }_{{\Lambda }(\underline{r})}$ be translation invariant. Further assume that $\eta \in \mathcal S\mathcal G^\textbf{1}({\Lambda }(\underline{r}))$ implies that the $\mathcal U$-KCM with initial condition ${\eta }\cdot \textbf{1}_{{\mathbb Z} ^2\setminus {\Lambda }(\underline{r})}$ can entirely infect ${\Lambda }(\underline{r})$. If we CBSEP-extend ${\Lambda }(\underline{r})$ by *l* in direction $u_i$, then for any ${\omega }\in {\Omega }_{{\mathbb Z} ^2\setminus {\Lambda }(\underline{r}+l\underline{v}_i)}$ and $\eta \in \mathcal S\mathcal G^{\omega }({\Lambda }(\underline{r}+l\underline{v}_i))$ the $\mathcal U$-KCM with initial condition ${\omega }\cdot \eta$ can entirely infect ${\Lambda }(\underline{r}+l\underline{v}_i)$.

#### Proof

By Definition 4.7, it suffices to prove that for each offset $x\in [0,s]$ the conclusion holds for $\eta \in \mathcal S\mathcal G^{\omega }_x({\Lambda }(\underline{r}+l\underline{v}_i))$. By Definition 4.7, this implies that the events $\mathcal S\mathcal G^\textbf{1}(xu_i+{\Lambda }(\underline{r}))\cap \mathcal S\mathcal T^{\omega }(xu_i+T(\underline{r},s-x,i))$ and $\mathcal S\mathcal G^\textbf{1}(xu_i+{\Lambda }(\underline{r}))\cap \mathcal S\mathcal T^{\omega }(xu_i+T(\underline{r},x,i+2k))$ hold. Moreover, by Definition 4.1, $\mathcal S\mathcal T^{{\omega }'}(T)\subset \mathcal T^{{\omega }'}(T)$ for any tube *T* and boundary condition ${\omega }'$. Therefore, we may apply Lemma 4.5 to each of the droplets ${\Lambda }(\underline{r}+x\underline{v}_i)$ and $xu_i+{\Lambda }(\underline{r}+(s-x)\underline{v}_i)$ (in directions $u_i$ and $u_{i+2k}$ respectively) to obtain the desired conclusion. $\square$

We next state the CBSEP analogue of Proposition 4.6, which is more involved, but also more efficient. Roughly speaking, we show that the time needed in order to relax on a CBSEP-extended droplet, is the product of four contributions: the Poincaré constant of the smaller droplet; the inverse probability of the symmetric traversability events in Definition 4.7; the cost of microscopic dynamics; the conditional probability of suitable contracted versions of the super good and symmetric traversability events, given the original ones (recall Sect. 2.3.1). The last two contributions turn out to be negligible, but the last one requires some care and make the statement somewhat technical.

#### Proposition 4.9

(CBSEP-extension relaxation). Assume that $\mathcal U$ has a finite number of stable directions. Let $i\in [4k]$. Let ${\Lambda }(\underline{r})$ be a droplet with $\underline{r}=q^{-O(C)}$ and side lengths at least $C^3$. Let $l\in (0,\ell ^{\textrm{mes}+}]$ be a multiple of ${\lambda }_i$. Let $\mathcal S\mathcal G^\textbf{1}({\Lambda }(\underline{r}))$ be a nonempty translation invariant decreasing event.

Denote ${\Lambda }_1=T(\underline{r},{\lambda }_i,i+2k)$, ${\Lambda }_2={\Lambda }(\underline{r}-{\lambda }_i\underline{v}_i)$ and ${\Lambda }_3=T(\underline{r}-{\lambda }_i\underline{v}_i,{\lambda }_i,i)$, so that ${\Lambda }(\underline{r}+{\lambda }_i\underline{v}_i)-{\lambda }_iu_i={\Lambda }_1\sqcup {\Lambda }_2\sqcup {\Lambda }_3$ and ${\Lambda }_2\cup {\Lambda }_3={\Lambda }(\underline{r})=({\Lambda }_1\cup {\Lambda }_2)+{\lambda }_iu_i$. Consider some nonempty decreasing events^4^$\overline{\mathcal S\mathcal G}({\Lambda }_2)\subset {\Omega }_{{\Lambda }_2}$, $\overline{\mathcal S\mathcal T}_{{\eta }_2}({\Lambda }_1)\subset {\Omega }_{{\Lambda }_1}$ and $\overline{\mathcal S\mathcal T}_{{\eta }_2}({\Lambda }_3)\subset {\Omega }_{{\Lambda }_3}$ for all ${\eta }_2\in \overline{\mathcal S\mathcal G}({\Lambda }_2)$. Assume that

18 $$\begin{aligned}{} & {} \left\{ {\eta }:{\eta }_{{\Lambda }_1}\in \overline{\mathcal S\mathcal T}_{{\eta }_{{\Lambda }_2}}({\Lambda }_1),{\eta }_{{\Lambda }_2}\in \overline{\mathcal S\mathcal G}({\Lambda }_2),{\eta }_{{\Lambda }_3}\in \overline{\mathcal S\mathcal T}_{{\eta }_{{\Lambda }_2}}({\Lambda }_3)\right\} \nonumber \\{} & {} \quad \subset \mathcal S\mathcal G^\textbf{1}({\Lambda }_1\cup {\Lambda }_2)\cap \mathcal S\mathcal G^\textbf{1}({\Lambda }_2\cup {\Lambda }_3). \end{aligned}$$

Set $$\overline{\mathcal S\mathcal G}({\Lambda }_1\cup {\Lambda }_2)=\{{\eta }:{\eta }_{{\Lambda }_2}\in \overline{\mathcal S\mathcal G}({\Lambda }_2),{\eta }_{{\Lambda }_1}\in \overline{\mathcal S\mathcal T}_{{\eta }_{{\Lambda }_2}}({\Lambda }_1)\}$$.

If we CBSEP-extend ${\Lambda }(\underline{r})$ by *l* in direction $u_i$, then $\mathcal S\mathcal G({\Lambda }(\underline{r}+l\underline{v}_i))$ is nonempty, translation invariant, decreasing and satisfies

$$\begin{aligned}{} & {} {\gamma }({\Lambda }(\underline{r}+l\underline{v}_i)) \leqslant \frac{{\mu }(\mathcal S\mathcal G^\textbf{1}({\Lambda }(\underline{r})))}{{\mu }(\mathcal S\mathcal G^\textbf{1}({\Lambda }(\underline{r}+l\underline{v}_i)))}\times \max \left( {\mu }^{-1}\left( \mathcal S\mathcal G^\textbf{1}({\Lambda }(\underline{r}))\right) ,{\gamma }({\Lambda }(\underline{r}))\right) \\{} & {} \quad \times \frac{e^{O(C^2)\log ^2(1/q)}}{{\mu }(\overline{\mathcal S\mathcal G}({\Lambda }_1\cup {\Lambda }_2)|\mathcal S\mathcal G^\textbf{1}({\Lambda }_1\cup {\Lambda }_2))\min _{{\eta }_2\in \overline{\mathcal S\mathcal G}({\Lambda }_2)}{\mu }(\overline{\mathcal S\mathcal T}_{{\eta }_2}({\Lambda }_3)|\mathcal S\mathcal T^{\textbf{0}}({\Lambda }_3))}. \end{aligned}$$

Proposition 4.9 is proved in “Appendix A.3” based on [24]. We referring the reader to [24, Sect. 4.3] for the principles behind Proposition 4.9 in a less technical framework, but let us briefly discuss the contracted events.

Equation (18) should be understood as follows. In the middle droplet ${\Lambda }_2$, which has the shape of ${\Lambda }(\underline{r})$, but contracted in direction $u_i$ by *O*(1), we require an event $\overline{\mathcal S\mathcal G}({\Lambda }_2)$. This event provides simultaneously as much of the structure of $\mathcal S\mathcal G^\textbf{1}({\Lambda }_1\cup {\Lambda }_2)$ and $\mathcal S\mathcal G^\textbf{1}({\Lambda }_2\cup {\Lambda }_3)$ (these regions both have the shape of ${\Lambda }(\underline{r})$), as one can hope for, given that we are missing a tube of length *O*(1) of these regions. Once such a favourable configuration ${\eta }_2\in \overline{\mathcal S\mathcal G}({\Lambda }_2)$ is fixed, the events $\overline{\mathcal S\mathcal T}_{{\eta }_{{\Lambda }_2}}({\Lambda }_1)$ and $\overline{\mathcal S\mathcal T}_{{\eta }_{{\Lambda }_2}}({\Lambda }_3)$ provide exactly the missing part of $\mathcal S\mathcal G^\textbf{1}({\Lambda }_1\cup {\Lambda }_2)$ and $\mathcal S\mathcal G^\textbf{1}({\Lambda }_2\cup {\Lambda }_3)$ respectively. In applications, these events necessarily need to be defined, taking into account the structure of $\mathcal S\mathcal G^\textbf{1}({\Lambda }(\underline{r}))$, on which we have made no assumptions at this point.

### Conditional probability tools

In both Propositions 4.6 and 4.9 our bounds feature certain conditional probabilities of SG events. We now provide two tools for bounding them.

The next result generalises [24, Corollary A.3], which relied on explicit computations unavailable in our setting. It shows that the offset of the core of a CBSEP-extended droplet (see Fig. 2b and recall the notation $\mathcal S\mathcal G_x^{\omega }$ from Definition 4.7) is roughly uniform. This result is the reason for the somewhat artificial Definition 3.8 of helping sets and Definition 4.1 of $\mathcal S\mathcal T$ (also see Remark 2.1).

#### Lemma 4.10

(Uniform core position). Assume that $\mathcal U$ has a finite number of stable directions. Fix $i\in [4k]$ and a symmetric droplet ${\Lambda }={\Lambda }(\underline{r}+l\underline{v}_i)$ obtained by CBSEP-extension by *l* in direction $u_i$. Assume that $l\leqslant \ell ^{\textrm{mes}+}$ is divisible by ${\lambda }_i$ and that the side lengths of ${\Lambda }(\underline{r})$ are at least $C^3$. Then for all $s\in [0,l]$ divisible by ${\lambda }_i$ and ${\omega },{\omega }'\in {\Omega }_{{\mathbb Z} ^2{\setminus }{\Lambda }}$

$$\begin{aligned} {\mu }\left( \left. \mathcal S\mathcal G^{\omega }_s({\Lambda })\right| \mathcal S\mathcal G^{{\omega }'}({\Lambda })\right) \geqslant q^{O(C)}. \end{aligned}$$

The proofs of Lemmas 4.10 and 4.11 are left to “Appendix B”. The latter vastly generalises [24, Lemma A.4] and is proved by different means. It is illustrated in Fig. 3. In words, Lemma 4.11 states in a quantitative way that the conditional probability of a tube of “critical” size ($q^{-{\alpha }+o(1)}$) being traversable, given that a slightly perturbed version of it (shifted spatially, with different boundary condition, width of the white strips in Fig. 2a, radii and length) is traversable, is not very low. We note that sizes other than the critical one are not important, so cruder bounds suffice.

#### Lemma 4.11

(Perturbing a tube). Let $i\in [4k]$ such that ${\alpha }(u_j)\leqslant {\alpha }$ for all $j\in (i-k,i+k)$. Let ${\Lambda }(\underline{r})$ be a droplet with side lengths $\underline{s}$ and let $T=T(\underline{r},l,i)$ be a tube. Assume that $l\in [{\Omega }(1),e^{q^{-o(1)}}]$, $s=\min _{i-k<j<i+k}s_j=q^{-{\alpha }+o(1)}$ and $\max _{i-k<j<i+k}s_j=q^{-{\alpha }+o(1)}$. For some ${\Delta }\in [C^2,s/W^2]$, let $\underline{r}'$ and $l'$ be such that $0\leqslant s_j-s_j'\leqslant O({\Delta })$ for all $j\in (i-k,i+k)$ and $0\leqslant l-l'\leqslant O({\Delta })$, where $\underline{s}'$ are the side lengths of the droplet ${\Lambda }(\underline{r}')$. Further let $x\in {\mathbb R} ^2$ be such that $\Vert x\Vert =O({\Delta })$ and $d,d'\in [0,O({\Delta })]$ with $d\leqslant d'$. Denoting $T'=T(\underline{r}',l',i)+x$, for any boundary conditions ${\omega }\in {\Omega }_{{\mathbb Z} ^2{\setminus } T}$ and ${\omega }'\in {\Omega }_{{\mathbb Z} ^2{\setminus } T'}$, we have

$$\begin{aligned}{} & {} {\mu }\left( \left. \mathcal T^{{\omega }'}_{d'}(T')\right| \mathcal T^{\omega }_{d}(T)\right) \geqslant q^{O(W)}\left( 1-(1-q^{\alpha })^{{\Omega }(s)}\right) ^{O({\Delta })}\\{} & {} \quad \times \left( 1-W{\Delta }/s-q^{1-o(1)}\right) ^{O(l)}. \end{aligned}$$

## Isotropic Models

For this section we assume $\mathcal U$ to be isotropic (class (g)). In this case the reasoning closely follows and generalises [24]. We treat internal and mesoscopic dynamics simultaneously, since for this class there is no difference between the two.

### Isotropic internal and mesoscopic dynamics

We start by defining the geometry of our droplets and the corresponding length scales. They are all symmetric and every 2*k*-th droplet is twice larger. Each such dilation is decomposed into 2*k* steps, so that their geometry fits the setting of our CBSEP-extensions from Sect. 4.3 (see Fig. 4a and recall Fig. 2b).

Recall Sect. 3.3 and the constant ${\varepsilon }$ from Sect. 3.4. Let $\underline{r}^{(0)}$ be a sequence of radii with $r_i^{(0)}=r_{i+2k}^{(0)}$ for all $i\in [2k]$, such that for all $i\in [4k]$, $r_i^{(0)}=\Theta (1/{\varepsilon })$ and the corresponding side length $s_i^{(0)}=\Theta (1/{\varepsilon })$ is a multiple of $2{\lambda }_{i+k}$. For any integer $m\geqslant 0$, $i\in [2k]$ and $n=2\,km+r$ with $r\in [2k]$ we define

19 $$\begin{aligned} s_{i}^{(n)}=s_{i+2k}^{(n)}=s^{(0)}_i2^{m}\times {\left\{ \begin{array}{ll}2&{}k\leqslant i< k+r\\ 1&{}\text {otherwise}\end{array}\right. }\end{aligned}$$

and ${\Lambda }^{(n)}={\Lambda }(\underline{r}^{(n)})$ with $\underline{r}^{(n)}$ the sequence of radii associated to $\underline{s}^{(n)}$ satisfying $r_{i}^{(n)}=r_{i+2k}^{(n)}$ for all $i\in [2k]$. Further set  (recall $\ell ^{\textrm{mes}+}$ from Sect. 3.4).

Note that, as claimed, ${\Lambda }^{(n)}$ are nested symmetric droplets extended in one direction at each step satisfying ${\Lambda }^{(2km)}=2^m{\Lambda }^{(0)}$. Moreover, they are nested so that we can define their SG events by extension (recall Definition 4.7 and Fig. 2b for CBSEP-extensions).

#### Definition 5.1

(*Isotropic SG*). Let $\mathcal U$ be isotropic. We say that ${\Lambda }^{(0)}$ is *SG* ($\mathcal S\mathcal G^\textbf{1}({\Lambda }^{(0)})$ occurs), if all sites in ${\Lambda }^{(0)}$ are infected. We then recursively define $\mathcal S\mathcal G^\textbf{1}({\Lambda }^{(n+1)})$ for $n\in [N^{\textrm{mes}+}]$ by CBSEP-extending ${\Lambda }^{(n)}$ in direction $u_n$ by $l^{(n)}=s_{n+k}^{(n)}=\Theta (2^{n/2k}/{\varepsilon })$ (recall from Sect. 3.2 that indices of directions and sequences are considered modulo 4*k* as needed and see Fig. 4a).

Recall from Sect. 3.6 that once $\mathcal S\mathcal G^\textbf{1}({\Lambda }^{(n)})$ is defined, so is ${\gamma }({\Lambda }^{(n)})$. We next prove a bound on ${\gamma }({\Lambda }^{(n)})$.

#### Theorem 5.2

Let $\mathcal U$ be isotropic (class (g)). Then for all $n\leqslant N^{\textrm{mes}+}$

$$\begin{aligned} {\gamma }\left( {\Lambda }^{(N^{\textrm{mes}+})}\right)&{}\leqslant \frac{\exp (1/(\log ^{C/2}(1/q)q^{\alpha }))}{{\mu }(\mathcal S\mathcal G^\textbf{1}({\Lambda }^{(N^{\textrm{mes}+})}))},&{\mu }\left( \mathcal S\mathcal G^\textbf{1}\left( {\Lambda }^{(n)}\right) \right)&{}\geqslant \exp \left( \frac{-1}{q^{\alpha }{\varepsilon }^2}\right) . \end{aligned}$$

The rest of Sect. 5.1 is devoted to the proof of Theorem 5.2. The bound on ${\mu }(\mathcal S\mathcal G^\textbf{1}({\Lambda }^{(n)}))$ is fairly standard in bootstrap percolation and could essentially be attributed to [7], but we prove it in Lemma 5.6, since we also need some better bounds on intermediate scales. Bounding ${\gamma }({\Lambda }^{(N^{\textrm{mes}+})})$ is more demanding and is done by iteratively applying Proposition 4.9, as suggested by Definition 5.1.

Note that ${\gamma }({\Lambda }^{(0)})=1$, since Eq. (15) is trivial, because $\mathcal S\mathcal G^\textbf{1}({\Lambda }^{(0)})$ is a singleton. We seek to apply Proposition 4.9, in order to recursively upper bound ${\gamma }({\Lambda }^{(n)})$ for all $n\leqslant N^{\textrm{mes}+}$. To that end, we need the following definition of contracted events. Since, in the language of Proposition 4.9, the events $\overline{\mathcal S\mathcal T}_{{\eta }_2}$ we define do not depend on ${\eta }_2$, we directly omit it from the notation.

#### Definition 5.3

(*Contracted isotropic events*). For $n=2km+r\in [N^{\textrm{mes}+}+1]$ with $r\in [2k]$, as in Proposition 4.9 with $\underline{r}=\underline{r}^{(n)}$, $l=l^{(n)}$ and $i=r$, let

20 $$\begin{aligned} {\Lambda }^{(n)}_1&{}=T\left( \underline{r}^{(n)},{\lambda }_r,n+2k\right) \nonumber \\ {\Lambda }^{(n)}_2&{}={\Lambda }\left( \underline{r}^{(n)}-{\lambda }_r\underline{v}_r\right) \nonumber \\ {\Lambda }^{(n)}_3&{}=T\left( \underline{r}^{(n)}-{\lambda }_r\underline{v}_r,{\lambda }_r,r\right) . \end{aligned}$$

If $n<2k$, we define $\overline{\mathcal S\mathcal T}({\Lambda }_1^{(n)})$, $\overline{\mathcal S\mathcal G}({\Lambda }_2^{(n)})$ and $\overline{\mathcal S\mathcal T}({\Lambda }_3^{(n)})$ to occur if ${\Lambda }_1^{(n)}$, ${\Lambda }_2^{(n)}$ and ${\Lambda }_3^{(n)}$ is fully infected respectively.

For $n\geqslant 2k$, we define $\overline{\mathcal S\mathcal T}({\Lambda }_1^{(n)})\subset {\Omega }_{{\Lambda }_1^{(n)}}$ (resp. $\overline{\mathcal S\mathcal T}({\Lambda }_3^{(n)})\subset {\Omega }_{{\Lambda }_3^{(n)}}$) to be the event that for every segment $S\subset {\Lambda }_1^{(n)}$ (resp. ${\Lambda }_3^{(n)}$) perpendicular to some $u_j$ with $j\ne r\pm k$ of length $2^m/(W{\varepsilon })$ the event $\mathcal H^W(S)$ occurs (recall Definition 3.9). Finally, for $n\geqslant 2k$, we define $\overline{\mathcal S\mathcal G}({\Lambda }_2^{(n)})$ as the intersection of the following events (see Fig. 4b)^5^

- $$\mathcal S\mathcal G^\textbf{1}({\Lambda }^{(n-2k)})$$;
- $$\mathcal S\mathcal T^\textbf{1}(T(\underline{r}^{(n-2k)},l^{(n-2k)}/2-{\lambda }_r,r))\cap \mathcal S\mathcal T^\textbf{1}(T(\underline{r}^{(n-2k)},l^{(n-2k)}/2-{\lambda }_r,r+2k))$$;
- for all $$i\in (0,2k)$$
  $$\begin{aligned}{} & {} \mathcal S\mathcal T^\textbf{1}_W\left( T\left( \underline{r}^{(n-2k+i)}-{\lambda }_r(\underline{v}_{r}+\underline{v}_{r+2k}),l^{(n-2k+i)}/2,r+i\right) \right) \\{} & {} \quad \cap \mathcal S\mathcal T^\textbf{1}_W\left( T\left( \underline{r}^{(n-2k+i)}-{\lambda }_r(\underline{v}_{r}+\underline{v}_{r+2k}),l^{(n-2k+i)}/2,r+i+2k\right) \right) . \end{aligned}$$
- for every $i\in [2k]$, $j\in [4k]$ and segment $S\subset {\Lambda }_2^{(n)}$, perpendicular to $u_j$ of length $2^m/(W{\varepsilon })$ at distance at most *W* from the $u_j$-side (parallel to *S*) of ${\Lambda }^{(n-2k+i)}$, the event $\mathcal H^W(S)$ holds.

In words, $\overline{\mathcal S\mathcal G}({\Lambda }_2^{(n)})$ is close to being the event that the central copy of ${\Lambda }^{(n-2k)}$ in ${\Lambda }_2^{(n)}$ is SG and several tubes are symmetrically traversable. Namely, for each $i\in [2k]$, the two tubes of equal length around ${\Lambda }^{(n-2k+i)}$ corresponding to a CBSEP-extension by $l^{(n-2k+i)}$ in direction $u_{r}$, finally reaching ${\Lambda }^{(n)}$ after 2*k* extensions. However, we have modified this event in the following ways. Firstly, the first extension is shortened by $2{\lambda }_r$, so that the final result after the 2*k* extensions fits inside ${\Lambda }_2^{(n)}$ and actually only its $u_{r+k}$ and $u_{r-k}$-sides are shorter than those of ${\Lambda }_2^{(n)}$ by ${\lambda }_r$ (see Fig. 4b). Secondly, the symmetric traversability events for tubes are required to occur with segments shortened by *W* (recall Definition 4.1) on each side. Finally, we roughly require *W* helping sets for the last *O*(*W*) lines of each tube, as well as the first *O*(*W*) outside the tube (without going out of ${\Lambda }^{(n)}_2$).

#### Lemma 5.4

(CBSEP-extension relaxation condition). For all $n\in [N^{\textrm{mes}+}]$ we have $\overline{\mathcal S\mathcal G}({\Lambda }_2^{(n)})\times \overline{\mathcal S\mathcal T}({\Lambda }_3^{(n)})\subset \mathcal S\mathcal G^\textbf{1}({\Lambda }_2^{(n)}\cup {\Lambda }_3^{(n)})$ and similarly for ${\Lambda }_1^{(n)}$ instead of ${\Lambda }_3^{(n)}$.

#### Proof

If $n<2k$, this follows directly from Definition 5.3, since $\overline{\mathcal S\mathcal G}({\Lambda }_2^{(n)})\times \overline{\mathcal S\mathcal T}({\Lambda }_3^{(n)})$ is only the fully infected configuration and similarly for ${\Lambda }_1^{(n)}$. We therefore assume that $n\geqslant 2k$ and set $n=2km+r$ with $r\in [2k]$.

We start with the first claim. Note that ${\Lambda }_2^{(n)}\cup {\Lambda }_3^{(n)}={\Lambda }^{(n)}$. Let ${\eta }\in \overline{\mathcal S\mathcal G}({\Lambda }_2^{(n)})\times \overline{\mathcal S\mathcal T}({\Lambda }_3^{(n)})$. We proceed by induction on *i* to show that ${\eta }_{{\Lambda }^{(i)}}\in \mathcal S\mathcal G^\textbf{1}({\Lambda }^{(i)})$ for $i\in [n-2k,n]$.

The base is part of Definition 5.3. Assume ${\eta }\in \mathcal S\mathcal G^\textbf{1}({\Lambda }^{(i)})$ for some $i\in [n-2k,n)$. Then by Definition 4.7, it suffices to check that

21 $$\begin{aligned} {\eta }\in \mathcal S\mathcal T^\textbf{1}\left( T\left( \underline{r}^{(i)},l^{(i)}/2,i\right) \right) \cap \mathcal S\mathcal T^\textbf{1}\left( T\left( \underline{r}^{(i)},l^{(i)}/2,i+2k\right) \right) , \end{aligned}$$

since then $${\eta }\in \mathcal S\mathcal G^\textbf{1}_{l^{(i)}/2}({\Lambda }^{(i+1)})\subset \mathcal S\mathcal G^\textbf{1}({\Lambda }^{(i+1)})$$.

Let us first consider the case $i=n-2k$ and assume for concreteness that *m* is even (so that $u_i=u_r$). Then

$$\begin{aligned} \eta \in \overline{\mathcal S\mathcal G}\left( {\Lambda }_2^{(n)}\right) \subset \mathcal S\mathcal T^\textbf{1}\left( T\left( \underline{r}^{(i)},l^{(i)}/2-{\lambda }_r,r\right) \right) , \end{aligned}$$

so by Lemma 4.3 it suffices to check that ${\eta }\in \mathcal S\mathcal T^\textbf{1}(u_i(l^{(i)}/2-{\lambda }_r)+T(\underline{r}^{(i)},{\lambda }_r,i))$, in order for the first symmetric traversability event in Eq. (21) to occur. We claim that this follows from ${\eta }\in \overline{\mathcal S\mathcal T}({\Lambda }_3^{(n)})$ and the fourth condition in Definition 5.3. To see this, notice that for each $j\in [4k]$ the $u_j$-side length of ${\Lambda }(\underline{r}^{(i)})$ satisfies $s_j^{(i)}=\Theta (s_j^{(0)}2^m)\gg 2^m/(W{\varepsilon })$ by Eq. (19). Further recall from Sect. 3.5 that $\mathcal H^W(S)\subset \mathcal H^{\omega }(S)$ for any segment *S* of length at least *C* and boundary condition ${\omega }$. Thus, for each of the segments in Definition 4.1 for the tube $u_i(l^{(i)}/2-{\lambda }_r)+T(\underline{r}^{(i)},{\lambda }_r,i)\subset {\Lambda }^{(n)}$, we have supplied not only a helping set, but in fact several *W*-helping sets. For directions $u_j$ with $j\in (r-k,r+k)\setminus \{r\}$, they are in ${\Lambda }^{(n)}_2$, while for $j=r$ they are found in ${\Lambda }^{(n)}_3$, if $k=1$ and *m* is even, and in ${\Lambda }^{(n)}_2$ otherwise (see Fig. 4b). Hence, the claim is established. For the second event in Eq. (21) the reasoning is the same except that when $k>1$ or *m* is even, the tube $T(\underline{r}^{(i)},l^{(i)}/2,i+2k)$ is entirely contained in ${\Lambda }_2^{(n)}$, so only $\overline{\mathcal S\mathcal G}({\Lambda }_2^{(n)})$ is needed.

We next turn to the case $i\in (n-2k,n)$, which is treated similarly. Indeed,

$$\begin{aligned} {\eta }\in \overline{\mathcal S\mathcal G}\left( {\Lambda }_2^{(n)}\right) \subset \mathcal S\mathcal T_W^\textbf{1}\left( T\left( \underline{r}^{(i)}-{\lambda }_r(\underline{v}_r+\underline{v}_{r+2k}),l^{(i)}/2,i\right) \right) . \end{aligned}$$

Comparing this tube to the desired one in Eq. (21), $T(\underline{r}^{(i)},l^{(i)}/2,i)$, we notice that the lengths and positions of their sides differ by *O*(1) (see Fig. 3). However, recalling Definition 4.1 and Fig. 2a, decreasing the width of each parallelogram there by ${\Omega }(W)\gg O(1)$ (using the event $\mathcal S\mathcal T_W^\textbf{1}$ rather than $\mathcal S\mathcal T^\textbf{1}$) is enough to compensate for this discrepancy (the shaded zones in Fig. 3 are empty in this case). It remains to ensure that the first and last *O*(1) segments in Definition 4.1 also have helping sets. But this is guaranteed by the fourth condition in Definition 5.3 and (depending on the values of *k*, *i* and *m*) $\overline{\mathcal S\mathcal T}({\Lambda }_3^{(n)})$ exactly as in the case $i=n-2k$.

Finally, the statement for ${\Lambda }_1^{(n)}$ is also proved analogously (with the offset for $i=n-2k$ modified by ${\lambda }_r$ in Eq. (21)), so the proof is complete. $\square$

By Lemma 5.4, Eq. (18) holds, so we may apply Proposition 4.9. This gives

22 $$\begin{aligned} {\gamma }\left( {\Lambda }^{(n+1)}\right) \leqslant {}&\max \left( {\mu }^{-1}\left( \mathcal S\mathcal G^\textbf{1}\left( {\Lambda }^{(n)}\right) \right) ,{\gamma }\left( {\Lambda }^{(n)}\right) \right) e^{O(C^2)\log ^2(1/q)}\nonumber \\&\times \frac{{\mu }(\mathcal S\mathcal G^\textbf{1}({\Lambda }^{(n)}))}{{\mu }(\mathcal S\mathcal G^\textbf{1}({\Lambda }^{(n+1)}))}{\mu }^{-1}\left( \left. \overline{\mathcal S\mathcal T}\left( {\Lambda }_3^{(n)}\right) \right| \mathcal S\mathcal T^\textbf{0}\left( {\Lambda }_3^{(n)}\right) \right) \nonumber \\&\times {\mu }^{-1}\left( \left. \overline{\mathcal S\mathcal T}\left( {\Lambda }_1^{(n)}\right) \cap \overline{\mathcal S\mathcal G}\left( {\Lambda }_2^{(n)}\right) \right| \mathcal S\mathcal G^\textbf{1}\left( {\Lambda }_1^{(n)}\cup {\Lambda }_2^{(n)}\right) \right) \end{aligned}$$

for $n\geqslant 2k$ and ${\gamma }({\Lambda }^{(n)})\leqslant e^{O(C^2)\log ^2(1/q)}$ for $n<2k$. We therefore assume that $n\geqslant 2k$. Recalling Definition 5.3, note that both $\overline{\mathcal S\mathcal T}({\Lambda }_1^{(n)})$ and $\overline{\mathcal S\mathcal T}({\Lambda }_3^{(n)})$ can be guaranteed by the presence of $O(W^2)$ well chosen infected *W*-helping sets, since only *O*(*W*) disjoint segments of length $2^m/(W{\varepsilon })$ perpendicular to $u_j$ for a given $j\in (r-k,r+k)$ can be fit in ${\Lambda }_1^{(n)}$ or ${\Lambda }^{(n)}_3$ (see Fig. 4b), so it suffices to have a *W*-helping set at each end of those. This and the Harris inequality, Eqs. (8) and (9), give

23 $$\begin{aligned}{} & {} {\mu }\left( \left. \overline{\mathcal S\mathcal T}\left( {\Lambda }_3^{(n)}\right) \right| \mathcal S\mathcal T^\textbf{0}\left( {\Lambda }_3^{(n)}\right) \right) \geqslant {\mu }\left( \overline{\mathcal S\mathcal T}\left( {\Lambda }_3^{(n)}\right) \right) \geqslant q^{W^{O(1)}}, \end{aligned}$$

24 $$\begin{aligned}{} & {} {\mu }\left( \left. \overline{\mathcal S\mathcal T}\left( {\Lambda }_1^{(n)}\right) \cap \overline{\mathcal S\mathcal G}\left( {\Lambda }_2^{(n)}\right) \right| \mathcal S\mathcal G^\textbf{1}\left( {\Lambda }_1^{(n)}\cup {\Lambda }_2^{(n)}\right) \right) \nonumber \\{} & {} \quad \geqslant q^{W^{O(1)}}{\mu }\left( \left. \overline{\mathcal S\mathcal G}\left( {\Lambda }_2^{(n)}\right) \right| \mathcal S\mathcal G^\textbf{1}\left( {\Lambda }_1^{(n)}\cup {\Lambda }_2^{(n)}\right) \right) . \end{aligned}$$

To deal with the last term we prove the following.

#### Lemma 5.5

(Contraction rate). Setting $m=\lfloor n/(2k)\rfloor \geqslant 1$, we have

25 $$\begin{aligned}{} & {} {\mu }\left( \left. \overline{\mathcal S\mathcal G}\left( {\Lambda }_2^{(n)}\right) \right| \mathcal S\mathcal G^\textbf{1}\left( {\Lambda }_1^{(n)}\cup {\Lambda }_2^{(n)}\right) \right) \nonumber \\{} & {} \quad \geqslant {\left\{ \begin{array}{ll} {\mu }\left( \overline{\mathcal S\mathcal G}\left( {\Lambda }_2^{(n)}\right) \right) &{}2^m\leqslant 1/\left( \log ^C(1/q)q^{\alpha }\right) ,\\ q^{O(C)}\frac{{\mu }(\overline{\mathcal S\mathcal G}({\Lambda }_2^{(n)}))}{{\mu }\left( \mathcal S\mathcal G^\textbf{1}\left( {\Lambda }^{(n-2k)}\right) \right) }&{}2^m\geqslant \log ^C(1/q)/q^{\alpha },\\ \exp \left( -2^m q^{1-o(1)}\right) &{}\text {otherwise}. \end{array}\right. } \end{aligned}$$

#### Proof

The first case follows from the Harris inequality Eq. (8).

For the other two cases we start by noting that ${\Lambda }^{(n)}_1\cup {\Lambda }^{(n)}_2={\Lambda }^{(n)}-{\lambda }_ru_r$ may be viewed as a 2*k*-fold CBSEP-extension of ${\Lambda }^{(n-2k)}$. Recalling the offset in Definition 4.7, set

$$\begin{aligned} \mathcal S\mathcal G^\bullet _0&{}=\mathcal S\mathcal G^\textbf{1}\left( {\Lambda }^{(n)}-{\lambda }_ru_r\right) ,\\ \mathcal S\mathcal G^\bullet _{i}&{}=\bigcap _{j=1}^{i}\mathcal S\mathcal G^\textbf{1}_{l^{(n-j)}/2}\left( {\Lambda }^{(n-j+1)}-{\lambda }_ru_r\right) \qquad i{}\in [1,2k-1],\\ \mathcal S\mathcal G^\bullet _{2k}&{}=\mathcal S\mathcal G^\bullet _{2k-1}\cap \mathcal S\mathcal G^\textbf{1}_{l^{(n-2k)}/2-{\lambda }_r}\left( {\Lambda }^{(n-2k+1)}-{\lambda }_ru_r\right) , \end{aligned}$$

so that $\mathcal S\mathcal G^\bullet _i$ corresponds to fixing the position of the core, which is a translate of ${\Lambda }^{(n-i)}$, inside ${\Lambda }^{(n)}-{\lambda }_ru_r$, but leaving its internal offsets unconstraint (see Fig. 4b). Thus, Lemma 4.10 applied 2*k* times gives

$$\begin{aligned} {\mu }\left( \left. \mathcal S\mathcal G^\bullet _{2k}\right| \mathcal S\mathcal G^\textbf{1}\left( {\Lambda }^{(n)}_1\cup {\Lambda }^{(n)}_2\right) \right) =\prod _{i=1}^{2k}{\mu }(\mathcal S\mathcal G^\bullet _{i}|\mathcal S\mathcal G^\bullet _{i-1})\geqslant q^{O(C)}. \end{aligned}$$

Expanding the definition of $\mathcal S\mathcal G^\bullet _{2k}$ via Definition 4.7, we see that this event is the intersection of $\mathcal S\mathcal G^\textbf{1}({\Lambda }^{(n-2k)})$ with some increasing events (symmetrically traversable tubes) independent of the latter. Thus, the Harris inequality Eq. (8) gives

26 $$\begin{aligned} {\mu }\left( \left. \overline{\mathcal S\mathcal G}\left( {\Lambda }_2^{(n)}\right) \right| \mathcal S\mathcal G^\textbf{1}\left( {\Lambda }_1^{(n)}\cup {\Lambda }_2^{(n)}\right) \right)&{}\geqslant q^{O(C)}{\mu }\left( \left. \overline{\mathcal S\mathcal G}\left( {\Lambda }_2^{(n)}\right) \right| \mathcal S\mathcal G^\bullet _{2k}\right) \nonumber \\&{}\geqslant q^{O(C)}{\mu }\left( \left. \overline{\mathcal S\mathcal G}\left( {\Lambda }_2^{(n)}\right) \right| \mathcal S\mathcal G^\textbf{1}\left( {\Lambda }^{(n-2k)}\right) \right) . \end{aligned}$$

Taking into account that $\overline{\mathcal S\mathcal G}({\Lambda }^{(n)}_2)\subset \mathcal S\mathcal G^\textbf{1}({\Lambda }^{(n-2k)})$ by Definition 5.3, this concludes the proof of the second case of Eq. (25).

For the third case, our starting point is again Eq. (26). This time we observe that $\mathcal S\mathcal G^\bullet _{2k}$ can be written as the intersection of $\mathcal S\mathcal G^\textbf{1}({\Lambda }^{(n-2k)})$ with 4*k* symmetric traversability events, each of which is a perturbed version (in the sense of Lemma 4.11 and Fig. 3) of the ones appearing in Definition 5.3 of $\overline{\mathcal S\mathcal G}({\Lambda }_2^{(n)})$. Thus, the Harris inequality Eq. (9) allows us to lower bound ${\mu }(\overline{\mathcal S\mathcal G}({\Lambda }_2^{(n)})|\mathcal S\mathcal G^\bullet _{2k})$ by

$$\begin{aligned} {\mu }(\mathcal W)\times {\mu }&\left( \mathcal S\mathcal T^\textbf{1}\left( T\left( \underline{r}^{(n-2k)},l^{(n-2k)}/2-{\lambda }_r,r\right) \right) \right. \\&\,\left| \mathcal S\mathcal T^\textbf{1}\left( T\left( \underline{r}^{(n-2k)},l^{(n-2k)}/2+{\lambda }_r,r\right) -{\lambda }_ru_r\right) \right) \\ \times {\mu }&\left( \mathcal S\mathcal T^\textbf{1}\left( T\left( \underline{r}^{(n-2k)},l^{(n-2k)}/2-{\lambda }_r,r+2k\right) \right) \right. \\&\,\left| \mathcal S\mathcal T^\textbf{1}\left( T\left( \underline{r}^{(n-2k)},l^{(n-2k)}/2-{\lambda }_r,r+2k\right) -{\lambda }_ru_r\right) \right) \\ \times \prod _{i=1}^{2k-1}\prod _{\xi =0}^1{\mu }&\left( \mathcal S\mathcal T_W^\textbf{1}\left( T\left( \underline{r}^{(n-2k+i)}-{\lambda }_r\left( \underline{v}_r+\underline{v}_{r+2k}\right) ,l^{(n-2k+i)}/2,r+i+2k\xi \right) \right) \right. \\&\,\left| \mathcal S\mathcal T^\textbf{1}\left( T\left( \underline{r}^{(n-2k+i)},l^{(n-2k+i)}/2,r+i+2k\xi \right) -{\lambda }_ru_r\right) \right) , \end{aligned}$$

where $\mathcal W$ is the event appearing in the last item of Definition 5.3.

Firstly, each of the above conditional probabilities is bounded by

$$\begin{aligned} q^{O(W)}\log ^{-C^{O(1)}}(1/q)\left( 1-q^{1-o(1)}\right) ^{O(2^m/{\varepsilon })}\geqslant \exp \left( -2^mq^{1-o(1)}\right) , \end{aligned}$$

using Lemma 4.11 with ${\Delta }=C^2$ and recalling that $2^m=q^{-{\alpha }}\log ^{O(C)}(1/q)$ and ${\alpha }\geqslant 1$. Secondly, ${\mu }(\mathcal W)\geqslant q^{W^{O(1)}}$ as in Eq. (23), concluding the proof of Eq. (25). We direct the reader to [24, Appendix A] for the details of an analogous argument in a simpler setting. $\square$

Iterating Eq. (22) and plugging Eq. (23) and (24) gives that ${\gamma }({\Lambda }^{(N^{\textrm{mes}+})})$ is at most

$$\begin{aligned} \frac{e^{O(C^2)N^{\textrm{mes}+}\log ^2(1/q)}q^{2N^{\textrm{mes}+}W^{O(1)}}}{{\mu }(\mathcal S\mathcal G^\textbf{1}({\Lambda }^{(N^{\textrm{mes}+})}))}\prod _{n=2k}^{N^{\textrm{mes}+}-1}{\mu }^{-1}\left( \overline{\mathcal S\mathcal G}\left( \left. {\Lambda }_2^{(n)}\right| \mathcal S\mathcal G^\textbf{1}\left( {\Lambda }^{(n)}_1\cup {\Lambda }_2^{(n)}\right) \right) \right) . \end{aligned}$$

Further recalling that $N^{\textrm{mes}+}=O(\log (\ell ^{\textrm{mes}+}))=O(C\log (1/q))$ and inserting Eq. (25), we obtain

$$\begin{aligned} {\gamma }\left( {\Lambda }^{(N^{\textrm{mes}+})}\right) \leqslant \frac{e^{q^{-{\alpha }+1-o(1)}}}{{\mu }(\mathcal S\mathcal G^\textbf{1}({\Lambda }^{(N^{\textrm{mes}+})}))}\prod _{n=2k}^{2^m\leqslant 1/(\log ^C(1/q)q^{\alpha }) }{\mu }^{-1}\left( \overline{\mathcal S\mathcal G}\left( {\Lambda }_2^{(n)}\right) \right) \\ \times \prod _{n:2^m\geqslant \log ^C(1/q)/q^{\alpha }}^{N^{\textrm{mes}+}-1}\frac{{\mu }(\mathcal S\mathcal G^\textbf{1}({\Lambda }^{(n-2k)}))}{{\mu }(\overline{\mathcal S\mathcal G}({\Lambda }_2^{(n)}))}. \end{aligned}$$

with $m=\lfloor n/(2k)\rfloor$. Hence, Theorem 5.2 follows immediately, once we prove Lemma 5.6 below.

#### Lemma 5.6

(Probability of super good droplets). For $n\in [2k,N^{\textrm{mes}+}]$ and $m=\lfloor n/(2k)\rfloor$, the following bounds hold:

27 $$\begin{aligned} {\mu }\left( \overline{\mathcal S\mathcal G}\left( {\Lambda }_2^{(n)}\right) \right)&{}\geqslant \exp \left( \frac{-1}{\log ^{C-3}(1/q)q^{\alpha }}\right)&\text {if }&2^m\leqslant \frac{1}{\log ^C(1/q)q^{\alpha }}, \end{aligned}$$

28 $$\begin{aligned} \frac{{\mu }(\overline{\mathcal S\mathcal G}({\Lambda }_2^{(n)}))}{{\mu }(\mathcal S\mathcal G^\textbf{1}({\Lambda }^{(n-2k)}))}&{}\geqslant q^{W^{O(1)}}&\text {if }&2^m\geqslant \frac{\log ^C(1/q)}{q^{\alpha }}, \end{aligned}$$

29 $$\begin{aligned} {\mu }\left( \mathcal S\mathcal G^\textbf{1}\left( {\Lambda }^{(n)}\right) \right)&{}\geqslant \exp \left( \frac{-1}{q^{\alpha }{\varepsilon }^2}\right) . \end{aligned}$$

#### Proof

Let us first bound ${\mu }(\mathcal S\mathcal G^\textbf{1}({\Lambda }^{(n)}))$ for $n\leqslant N^{\textrm{mes}+}$ by induction, starting with the trivial bound

$$\begin{aligned} {\mu }\left( \mathcal S\mathcal G^\textbf{1}\left( {\Lambda }^{(2k)}\right) \right) \geqslant q^{|{\Lambda }^{(2k)}|}\geqslant q^{O(1/{\varepsilon })}. \end{aligned}$$

From Definition 4.7, translation invariance and Eq. (16), for $n\in [2k,N^{\textrm{mes}+}-1]$ we have

30 $$\begin{aligned} {\mu }\left( \mathcal S\mathcal G^\textbf{1}\left( {\Lambda }^{(n+1)}\right) \right)&{}\geqslant {\mu }\left( \mathcal S\mathcal G_0^\textbf{1}\left( {\Lambda }^{(n+1)}\right) \right) \nonumber \\&{}={\mu }\left( \mathcal S\mathcal G^\textbf{1}\left( {\Lambda }^{(n)}\right) \right) {\mu }\left( \mathcal S\mathcal T^\textbf{1}\left( T\left( \underline{r}^{(n)},l^{(n)},n\right) \right) \right) \nonumber \\&{}\geqslant q^{O(1/{\varepsilon })}\prod _{i=2k}^n{\mu }\left( \mathcal S\mathcal T^\textbf{1}\left( T\left( \underline{r}^{(i)},l^{(i)},i\right) \right) \right) , \end{aligned}$$

so we need to bound the last term. Applying Definition 4.1, Lemma 4.2 and the Harris inequality Eq. (7) and then Observation 3.11, we get

31 $$\begin{aligned} {\mu }\left( \mathcal S\mathcal T^\textbf{1}\left( T\left( \underline{r}^{(n)},l^{(n)},n\right) \right) \right) \geqslant {}&q^{O(W)}\prod _{j,m'}\mathcal H_{C^2}\left( S_{j,m'}\right) \nonumber \\ \geqslant {}&q^{O(W)}\left( 1-e^{-q^{\alpha }2^m/O({\varepsilon })}\right) ^{O(2^m/{\varepsilon })}\nonumber \\ \geqslant {}&q^{O(W)}{\left\{ \begin{array}{ll} \left( q^{\alpha }2^{m-1}\right) ^{C2^m/{\varepsilon }}&{}2^m\leqslant 1/q^{{\alpha }}\\ \exp \left( -2^m\exp \left( -q^{\alpha }2^m\right) \right) &{}2^m> 1/q^{{\alpha }}, \end{array}\right. } \end{aligned}$$

where the product runs over the segments $S_{j,m'}$ appearing in Definition 4.1 for the event $\mathcal S\mathcal T^\textbf{1}(T(\underline{r}^{(n)},l^{(n)},n))=\mathcal T^\textbf{1}(T(\underline{r}^{(n)},l^{(n)},n))$ (the last equality holds, since $\mathcal U$ is isotropic). Plugging Eq. (31) into Eq. (30) and iterating, we get

32 $$\begin{aligned} {\mu }\left( \mathcal S\mathcal G^\textbf{1}\left( {\Lambda }^{(n)}\right) \right) \geqslant {\left\{ \begin{array}{ll} \exp \left( -1/\left( \log ^{C-2}(1/q)q^{\alpha }\right) \right) &{}2^m\leqslant 1/\left( \log ^C(1/q)q^{\alpha }\right) \\ \exp \left( -1/\left( q^{\alpha }{\varepsilon }^2\right) \right) &{}2^m> 1/\left( \log ^C(1/q)q^{\alpha }\right) \end{array}\right. } \end{aligned}$$

since $N^{\textrm{mes}+}\leqslant O(C)\log (1/q)$. This proves Eq. (29).

Recalling Definition 5.3, as in the proof of Lemma 5.5, we have that for any $n\in [2k,N^{\textrm{mes}+}]$

$$\begin{aligned}{} & {} {\mu }\left( \overline{\mathcal S\mathcal G}\left( {\Lambda }_2^{(n)}\right) \right) ={\mu }(\mathcal W){\mu }\left( \mathcal S\mathcal G^\textbf{1}\left( {\Lambda }^{(n-2k)}\right) \right) \\{} & {} \quad \times {}\prod _{\xi =0}^1{\mu }\left( \mathcal S\mathcal T^\textbf{1}\left( T\left( \underline{r}^{(n-2k)},l^{(n-2k)}/2-{\lambda }_r,r+2k\xi \right) \right) \right) \\{} & {} \quad \times {} \prod _{\xi =0}^1\prod _{i=1}^{2k-1}{\mu }\left( \mathcal S\mathcal T_W^\textbf{1}\left( T\left( \underline{r}^{(n-2k+i)}-{\lambda }_r\left( \underline{v}_r+\underline{v}_{r+2k}\right) ,l^{(n-2k)}/2,r+2k\xi \right) \right) \right) , \end{aligned}$$

where $\mathcal W$ is the event from the last item of Definition 5.3 and $r=n-2km$. As in the proof of Lemma 5.5, we have ${\mu }(\mathcal W)\geqslant q^{W^{O(1)}}$, while the factors in the products can be bounded exactly as in Eq. (31), entailing Eqs. (27) and (28), since we already have Eq. (32). $\square$

### CBSEP global dynamics

For the global dynamics we need to recall the global CBSEP mechanism introduced in [24]. It is useful not only for class (g), but also other unrooted models—classes (d) and (f).

Let ${\Lambda }^{\textrm{mes}-}$ and ${\Lambda }^{\textrm{mes}+}$ be droplets with side lengths $\Theta (\ell ^{\textrm{mes}-})$ and $\Theta (\ell ^{\textrm{mes}+})$ respectively (recall Sect. 3.4). Consider a tiling of ${\mathbb R} ^2$ with square boxes $Q_{i,j}=[0,\ell ^{\textrm{mes}})\times [0,\ell ^{\textrm{mes}})+\ell ^{\textrm{mes}}(i,j)$ for $(i,j)\in {\mathbb Z} ^2$.

#### Definition 5.7

(*Good and super good boxes*). We say that the box $Q_{i,j}$ is *good* if for every segment $S\subset Q_{i,j}$, perpendicular to some $u\in {\widehat{\mathcal S}}$ of length at least ${\varepsilon }\ell ^{\textrm{mes}-}$, $\mathcal H^W(S)$ occurs (recall Definition 3.9). We denote the corresponding event by $\mathcal G_{i,j}$. We further say that $\mathcal G({\Lambda }^{\textrm{mes}+})$ occurs if for every segment $S\subset {\Lambda }^{\textrm{mes}+}$, perpendicular to some $u\in {\widehat{\mathcal S}}$ of length at least $3{\varepsilon }\ell ^{\textrm{mes}-}$, the event $\mathcal H^W(S)$ occurs.

Let $\mathcal S\mathcal G^\textbf{1}({\Lambda }^{\textrm{mes}-})\subset {\Omega }_{{\Lambda }^{\textrm{mes}+}}$ be a nonempty translation invariant event. We say that $Q_{i,j}$ is *super good* if it is good and $\mathcal S\mathcal G^\textbf{1}(x+{\Lambda }^{\textrm{mes}-})$ occurs for some $x\in {\mathbb Z} ^2$ such that $x+{\Lambda }^{\textrm{mes}-}\subset Q_{i,j}$. We denote the corresponding event by $\mathcal S\mathcal G_{i,j}$.

In words, good boxes $Q_{i,j}$ and droplets ${\Lambda }^{\textrm{mes}+}$ contain *W*-helping sets in sufficient supply for a SG translate of ${\Lambda }^{\textrm{mes}-}$ to be able to move inside the box or droplet containing it. Our choice of $\ell ^{\textrm{mes}-}$ makes being good so likely that we are able to assume that all boxes and droplets are good at all times. Finally, a box is SG, if it also contains a SG translate of ${\Lambda }^{\textrm{mes}-}$ that we wish to move around. Thus, when looking at SG boxes, we essentially see a two-dimensional CBSEP dynamics, which leads to the following bound.

#### Proposition 5.8

(Global CBSEP relaxation). Let $\mathcal U$ be unrooted (classes (d), (f) and (g)). Let $T=\exp (\log ^4(1/q)/q^{\alpha })$. Assume that $\mathcal S\mathcal G^\textbf{1}({\Lambda }^{\textrm{mes}+})$ and $\mathcal S\mathcal G^\textbf{1}({\Lambda }^{\textrm{mes}-})$ are nonempty translation invariant decreasing events such that the following conditions hold:

1. $$(1-{\mu }(\mathcal S\mathcal G^\textbf{1}({\Lambda }^{\textrm{mes}-})))^TT^4=o(1)$$;
2. for all $x\in {\mathbb Z} ^2$ such that $x+{\Lambda }^{\textrm{mes}-}\subset {\Lambda }^{\textrm{mes}+}$ we have
  $$\begin{aligned} \mathcal S\mathcal G^\textbf{1}(x+{\Lambda }^{\textrm{mes}-})\cap \mathcal G({\Lambda }^{\textrm{mes}+})\subset \mathcal S\mathcal G^\textbf{1}({\Lambda }^{\textrm{mes}+}). \end{aligned}$$

Then

$$\begin{aligned} {\mathbb E} _{{\mu }}[{\tau }_0]\leqslant {\gamma }\left( {\Lambda }^{\textrm{mes}+}\right) \frac{\log (1/{\mu }(\mathcal S\mathcal G^\textbf{1}({\Lambda }^{\textrm{mes}-})))}{q^{O(C)}}. \end{aligned}$$

We omit the proof, which is identical to [24, Sect. 5], given Definition 5.7,^6^ and turn to the proof of Theorem 1 for the isotropic class (g).

#### Proof of Theorem 1(g)

Let $\mathcal U$ be isotropic. Recall the droplets ${\Lambda }^{(n)}$ from Sect. 5.1. Set  and ${\Lambda }^{\textrm{mes}-}={\Lambda }^{(N^{\textrm{mes}-})}$. Thus, the side lengths of ${\Lambda }^{\textrm{mes}-}$ and ${\Lambda }^{\textrm{mes}+}$ are indeed $\Theta (\ell ^{\textrm{mes}-})$ and $\Theta (\ell ^{\textrm{mes}+})$ respectively by Eq. (19). By Theorem 5.2, condition (1) of Proposition 5.8 is satisfied:

$$\begin{aligned} (1-{\mu }(\mathcal S\mathcal G^\textbf{1}({\Lambda }^{\textrm{mes}-})))^TT^4&{}\leqslant (1-e^{-1/(q^{\alpha }{\varepsilon }^2)})^TT^4\leqslant T^4e^{-e^{\log ^4(1/q)/q^{\alpha }-1/(q^{\alpha }{\varepsilon }^2)}}\\&{}\leqslant e^{4\log ^4(1/q)/q^{\alpha }-e^{\log ^4(1/q)/(2q^{\alpha })}}=o(1). \end{aligned}$$

We next seek to verify condition (2). Proceeding by induction on $n\in [N^{\textrm{mes}-},N^{\textrm{mes}+}]$, it suffices to show that for any $n\in [N^{\textrm{mes}-},N^{\textrm{mes}+})$ and $x,y\in {\mathbb Z} ^2$ such that $x+{\Lambda }^{(n)}\subset y+{\Lambda }^{(n+1)}\subset {\Lambda }^{\textrm{mes}+}$, we have

33 $$\begin{aligned} \mathcal G({\Lambda }^{\textrm{mes}+})\cap \mathcal S\mathcal G^\textbf{1}(x+{\Lambda }^{(n)})\subset \mathcal S\mathcal G^\textbf{1}(y+{\Lambda }^{(n+1)}). \end{aligned}$$

Recalling Definitions 4.7 and 5.1, we see that it suffices to show that for any tube *T* of the form $z+T(\underline{r}^{(n)},l,j)$ for some $l>0$, $j\in [4k]$ and $z\in {\mathbb Z} ^2$ satisfying $T\subset y+{\Lambda }^{(n+1)}$ also verifies $\mathcal G({\Lambda }^{\textrm{mes}+})\subset \mathcal S\mathcal T^\textbf{1}(T)$. Further recalling Definition 4.1, we see that it suffices to show that on $\mathcal G({\Lambda }^{\textrm{mes}+})$, each segment of length $\min _{j\in [4k]}s_j^{(n)}-C^2-O(1)$ perpendicular to $u_j$ for some $j\in [4k]$ contains an infected *W*-helping set (recall from Sect. 3.5.2 that $\mathcal H^W_d(S)\subset \mathcal H^{\omega }_d(S)$). Hence, Eq. (33) follows from Definition 5.7, since

$$\begin{aligned} \min _{j\in [4k]}s_j^{(n)}-C^2-O(1)=\Theta (\ell ^{\textrm{mes}-})\geqslant 3{\varepsilon }\ell ^{\textrm{mes}-}. \end{aligned}$$

Thus, we may apply Proposition 5.8. Further plugging the bounds from Theorem 5.2, we recover

$$\begin{aligned} {\mathbb E} _{{\mu }}[{\tau }_0]&{}\leqslant \frac{\exp (1/(\log ^{C/2}(1/q)q^{\alpha }))}{{\mu }(\mathcal S\mathcal G^\textbf{1}({\Lambda }^{(N^{\textrm{mes}+})}))}\frac{1}{q^{\alpha }{\varepsilon }^2q^{O(C)}}\\&{}\leqslant \frac{\exp (1/(\log ^{C/3}(1/q)q^{\alpha }))}{{\mu }(\mathcal S\mathcal G^\textbf{1}({\Lambda }^{(N^{\textrm{mes}+})}))}\leqslant \exp \left( \frac{1+o(1)}{{\varepsilon }^2q^{\alpha }}\right) , \end{aligned}$$

concluding the proof. $\square$

## Unbalanced Unrooted Models

In this section we assume $\mathcal U$ is unbalanced unrooted (class (d)). We deal with the internal, mesoscopic and global dynamics separately. The internal dynamics is very simple and already known since [22]. The mesoscopic and global ones are similar to the ones of Sect. 5 with some adaptations needed for the mesoscopic one.

### Unbalanced internal dynamics

For unbalanced unrooted $\mathcal U$ (class (d)) the SG event on to the internal scale consists simply in having an infected ring of thickness *W* (see Fig. 5). Recall $\ell ^{\textrm{int}}$ from Sect. 3.4.

#### Definition 6.1

(*Unbalanced unrooted internal SG*). Assume $\mathcal U$ is unbalanced unrooted. Let ${\Lambda }^{(0)}={\Lambda }(\underline{r}^{(0)})$ be a droplet with side lengths  for $j\in [4k]$. We say that ${\Lambda }^{(0)}$, is *super good* ($\mathcal S\mathcal G^\textbf{1}({\Lambda }^{(0)})$ occurs) if all sites in ${\Lambda }^{(0)}\setminus {\Lambda }(\underline{r}^{(0)}-W\underline{1})$ are infected.

The following result was proved in [22, Lemma 4.10] and provides the main contribution to the scaling for this class (see Table 2b).

#### Proposition 6.2

For unbalanced unrooted $\mathcal U$ (class (d)) we have

$$\begin{aligned} \max \left( {\gamma }\left( {\Lambda }^{(0)}\right) ,{\mu }^{-1}\left( \mathcal S\mathcal G^\textbf{1}\left( {\Lambda }^{(0)}\right) \right) \right) \leqslant q^{-O(W\ell ^{\textrm{int}})}\leqslant \exp \left( C^3\log ^2(1/q)/q^{\alpha }\right) . \end{aligned}$$

### CBSEP mesoscopic dynamics

Since $\mathcal U$ is unbalanced unrooted, we may assume w.l.o.g. that ${\alpha }(u_j)\leqslant {\alpha }$ for all $j\in [4k]\setminus \{k,-k\}$. We only use 4*k* scales for the mesoscopic dynamics. Recall Sects. 3.3 and 3.4. For $i\in [0,2k]$ let ${\Lambda }^{(i)}={\Lambda }(\underline{r}^{(i)})$ be the symmetric droplet centered at 0 with $\underline{r}^{(i)}$ such that its associated side lengths areFor $i\in (2k,4k]$, we define ${\Lambda }^{(i)}$ similarly by34These droplets are exactly as in Fig. 4a, except that the extensions are much longer. More precisely, we have ${\Lambda }^{(i+1)}={\Lambda }(\underline{r}^{(i)}+l^{(i)}(\underline{v}_i+\underline{v}_{i+2k})/2)$ with $l^{(i)}=s_{i+k}^{(i+1)}-s_{i+k}^{(i)}$, so that $l^{(i)}=(1-q^{C-{\alpha }+o(1)})\ell ^{\textrm{mes}-}$ if $i\in [2k]$ and $l^{(i)}=(1-O({\delta }))\ell ^{\textrm{mes}+}$ if $i\in [2k,4k)$. In particular, the droplets ${\Lambda }^{(n)}$ for $n\in [4k+1]$ are nested in such a way that allows us to define their SG events by extension, as in Definition 5.1 (also recall Definition 6.1 for $\mathcal S\mathcal G^\textbf{1}({\Lambda }^{(0)})$ and Definition 4.7 and Fig. 2b for CBSEP-extensions).

#### Definition 6.3

(*Unbalanced unrooted mesoscopic SG*). Let $\mathcal U$ be unbalanced unrooted. For $n\in [4k]$ we define $\mathcal S\mathcal G^\textbf{1}({\Lambda }^{(n+1)})$ by CBSEP-extending ${\Lambda }^{(n)}$ by $l^{(n)}$ in direction $u_n$.

With this definition we aim to prove the following (recall ${\gamma }({\Lambda }^{(4k)})$ from Sect. 3.6).

#### Theorem 6.4

Let $\mathcal U$ be unbalanced unrooted (class (d)). Then

$$\begin{aligned} \max \left( {\gamma }\left( {\Lambda }^{(4k)}\right) ,{\mu }^{-1}\left( \mathcal S\mathcal G^\textbf{1}\left( {\Lambda }^{(2k)}\right) \right) \right) \leqslant \exp \left( \frac{\log ^2(1/q)}{{\delta }q^{\alpha }}\right) . \end{aligned}$$

The remainder of Sect. 6.2 is dedicated to the proof of Theorem 6.4. Naturally, Theorem 6.4 results from 4*k* applications of Proposition 4.9 and using Proposition 6.2 as initial input. The second step is somewhat special (see Fig. 5a), since there we need to take into account the exact structure of $\mathcal S\mathcal G^\textbf{1}({\Lambda }^{(0)})$ from Definition 6.1 in the definition of the contracted events appearing in Proposition 4.9. For the remaining steps the reasoning is identical to the proof of Theorem 5.2, but computations are simpler, since there are only boundedly many scales. Following the proof of Theorem 5.2, we start by defining our contracted events (cf. Definition 5.3).

#### Definition 6.5

(*Contracted unbalanced unrooted events*). For $n=2\,km+r\in [4k+1]$ and $r\in [2k]$, define ${\Lambda }_1^{(n)},{\Lambda }^{(n)}_2,{\Lambda }_3^{(n)}$ by Eq. (20).

Let $\overline{\mathcal S\mathcal T}({\Lambda }^{(0)}_1)$ (resp. $\overline{\mathcal S\mathcal T}({\Lambda }^{(0)}_3)$) be the events that ${\Lambda }^{(0)}_1$ (resp. ${\Lambda }^{(0)}_3$) is fully infected and $\overline{\mathcal S\mathcal G}({\Lambda }^{(0)}_2)$ be the event that ${\Lambda }^{(0)}_2\setminus {\Lambda }(\underline{r}^{(0)}-2W\underline{1})$ is fully infected.

Let $\overline{\mathcal S\mathcal G}({\Lambda }^{(1)}_2)$ occur if the following all hold (see Fig. 5a):^5^

- $$\mathcal S\mathcal T^\textbf{1}_W(T(\underline{r}^{(0)}-{\lambda }_1\underline{v}_1,l^{(0)}/2,0))$$ occurs,
- $({\Lambda }(\underline{r}^{(0)}+W\underline{1})\setminus {\Lambda }(\underline{r}^{(0)}-2W\underline{1}))\cap {\Lambda }_2^{(1)}$ is fully infected,
- $$\mathcal S\mathcal T^\textbf{1}_W(T(\underline{r}^{(0)}-{\lambda }_1\underline{v}_1,l^{(0)}/2,2k))$$ occurs,
- for all $j\ne \pm k$ and segment $S\subset {\Lambda }_2^{(1)}$, perpendicular to $u_j$ at distance at most *W* from the $u_j$-side of ${\Lambda }_2^{(1)}$ and of length $\ell ^{\textrm{int}}/W$, the event $\mathcal H^W(S)$ occurs.

Further let $\overline{\mathcal S\mathcal T}({\Lambda }_1^{(1)})$ occur if the following both hold (see Fig. 5a):

- ${\Lambda }(\underline{r}^{(0)}+W\underline{1})\cap {\Lambda }_1^{(1)}$ is fully infected,
- for all $j\ne \pm k$ and segment $S\subset {\Lambda }_1^{(1)}$ perpendicular to $u_j$ of length $\ell ^{\textrm{int}}/W$ the event $\mathcal H^W(S)$ occurs.

We define $\overline{\mathcal S\mathcal T}({\Lambda }_3^{(1)})$ analogously.

Let $i\in [2,4k)$. We say that $\overline{\mathcal S\mathcal T}({\Lambda }^{(i)}_1)$ occurs (see Fig. 5b) if for all $j\in [4k]$ and $m\in \{i-1,i\}$ every segment $S\subset {\Lambda }^{(i)}_1$ perpendicular to $u_j$ of length $s_j^{(m)}/W$ at distance at most *W* from the $u_j$-side (parallel to *S*) of ${\Lambda }^{(m)}$, the event $\mathcal H^W(S)$ occurs. We define $\overline{\mathcal S\mathcal T}({\Lambda }^{(i)}_3)$ similarly. Let $\overline{\mathcal S\mathcal G}({\Lambda }^{(i)}_2)$ occur if the following all hold (see Fig. 5b):

- $$\mathcal S\mathcal G^\textbf{1}({\Lambda }^{(i-2)})$$ occurs;
- for each $m\in \{0,2k\}$ the following occurs
  $$\begin{aligned}{} & {} \mathcal S\mathcal T^\textbf{1}_W\left( T\left( \underline{r}^{(i-2)},l^{(i-2)}/2-\sqrt{W},i-2+m\right) \right) \\{} & {} \quad \cap \mathcal S\mathcal T^\textbf{1}_W\left( T\left( \underline{r}^{(i-1)}-\sqrt{W}\left( \underline{v}_i+\underline{v}_{i+2k}\right) ,l^{(i-1)}/2-\sqrt{W},i-1+m\right) \right) ; \end{aligned}$$
- for all $j\in [4k]$, $m\in \{i-2,i-1,i\}$ and segment $S\subset {\Lambda }_2^{(i)}$, perpendicular to $u_j$ of length $s_j^{(m)}/W$ at distance at most *W* from the $u_j$-side of ${\Lambda }^{(m)}$, the event $\mathcal H^W(S)$ holds.

Before moving on, let us make a few comments on how Definition 6.5 of $\overline{\mathcal S\mathcal G}({\Lambda }^{(n)}_1)$ and $\overline{\mathcal S\mathcal T}({\Lambda }^{(n)}_3)$ is devised. Recall that our goal is to satisfy Eq. (18), that is, $\overline{\mathcal S\mathcal G}({\Lambda }_2^{(n)})\times \overline{\mathcal S\mathcal T}({\Lambda }_3^{(n)})\subset \mathcal S\mathcal G^\textbf{1}({\Lambda }^{(n)})$, so as to apply Proposition 4.9. For that reason, for the various values of *n*, we have required the (more than) parts of the event $\mathcal S\mathcal G^\textbf{1}({\Lambda }^{(n)})$ which can be witnessed in each of ${\Lambda }_2^{(n)}$ and ${\Lambda }_3^{(n)}$. Since $\mathcal S\mathcal G^\textbf{1}({\Lambda }^{(0)})$ corresponds to an infected ring of width roughly *W* and radius being fully infected (see Definition 6.1), we have required for $n\in \{0,1\}$ a ring of the same radius, but three times thicker to be infected. Similarly to Definition 5.3, we have slightly reduced the length of traversable tubes present in (recall Definition 6.3), but thinned the corresponding parallelograms in Fig. 2b. We have further asked for *W*-helping sets around all boundaries so as to compensate for the shortening of the tubes. The construction takes advantage of the fact that for $n\geqslant 2$ the droplet ${\Lambda }^{(n-2)}$ is far from the boundaries of ${\Lambda }^{(n)}$ (see Fig. 5b), so the event $\mathcal S\mathcal G^\textbf{1}({\Lambda }^{(n-2)})$ can be directly incorporated into $\overline{\mathcal S\mathcal G}({\Lambda }^{(n)}_2)$, rather than being decomposed into one part in ${\Lambda }_2^{(n)}$ and one in ${\Lambda }_3^{(n)}$.

#### Lemma 6.6

(CBSEP-extension relaxation condition). For all $n\in [4k]$ we have $\overline{\mathcal S\mathcal G}({\Lambda }_2^{(n)})\times \overline{\mathcal S\mathcal T}({\Lambda }_3^{(n)})\subset \mathcal S\mathcal G^\textbf{1}({\Lambda }_2^{(n)}\cup {\Lambda }_3^{(n)})$ and similarly for ${\Lambda }_1^{(n)}$ instead of ${\Lambda }_3^{(n)}$.

#### Proof

The proof for $n\geqslant 2$ is essentially identical to the one of Lemma 5.4 and $n=0$ is immediate from Definitions 6.1 and 6.5. We therefore focus on the case $n=1$ and on ${\Lambda }_3^{(1)}$, since ${\Lambda }_1^{(1)}$ is treated analogously. Assume $\overline{\mathcal S\mathcal G}({\Lambda }_2^{(1)})$ and $\overline{\mathcal S\mathcal T}({\Lambda }_3^{(1)})$ occur. Recalling Definition 4.7, it suffices to prove that $\mathcal S\mathcal G^\textbf{1}_{l^{(0)}/2}({\Lambda }^{(1)})$ occurs.

Firstly, note that

$$\begin{aligned} \mathcal S\mathcal T^\textbf{1}\left( T\left( \underline{r}^{(0)},l^{(0)}/2,2k\right) \right) \supset \mathcal S\mathcal T^\textbf{1}_W\left( T\left( r^{(0)}-{\lambda }_1\underline{v}_1,l^{(0)}/2,2k\right) \right) , \end{aligned}$$

recalling from Eq. (10) that $\langle \underline{v}_1,\underline{e}_j\rangle =0$ for all $j\in \{k+1,\dots ,3k-1\}$ and $\langle \underline{v}_1,\underline{e}_j\rangle \leqslant O(1)\ll W$ for $j\in \{k,3k\}$. Similarly, for any $\eta \in {\Omega }_{{\Lambda }^{(1)}}$ we have

$$\begin{aligned} \eta \in \mathcal S\mathcal T^\textbf{1}_W\left( T\left( \underline{r}^{(0)}-{\lambda }_1\underline{v}_1,l^{(0)}/2,0\right) \right) \Rightarrow \eta \in \mathcal S\mathcal T^{\eta _{{\Lambda }^{(1)}\setminus T}\cdot \textbf{1}_{{\mathbb Z} ^2\setminus {\Lambda }^{(1)}}}(T), \end{aligned}$$

where $T=(\underline{r}^{(0)},l^{(0)}/2-{\lambda }_1/\langle u_1,u_0\rangle ,0)$. Furthermore, the fourth condition in the definition of $\overline{\mathcal S\mathcal G}({\Lambda }_2^{(1)})$ and the second condition in the definition of $\overline{\mathcal S\mathcal T}({\Lambda }_3^{(1)})$ (see Definition 6.5) imply the occurrence of $\mathcal S\mathcal T^\textbf{1}(u_0(l^{(0)}/2-{\lambda }_1/\langle u_1,u_0\rangle )+T(\underline{r}^{(0)},{\lambda }_1/\langle u_1,u_0\rangle ,0))$. Using Lemma 4.3 to combine these two facts, we obtain that $\mathcal S\mathcal T^\textbf{1}(\underline{r}^{(0)},l^{(0)}/2,0)$ occurs.

Thus, it remains to show that $\mathcal S\mathcal G^\textbf{1}({\Lambda }^{(0)})$ occurs. But, in view of Definition 6.1, this is the case by the second condition in the definition of $\overline{\mathcal S\mathcal G}({\Lambda }_2^{(1)})$ and the first condition of $\overline{\mathcal S\mathcal T}({\Lambda }_3^{(1)})$ (see Definition 6.5). $\square$

By Lemma 6.6, Eq. (18) holds, so we may apply Proposition 4.9. Together with the Harris inequality Eq. (8), this gives

35 $$\begin{aligned} {\gamma }\left( {\Lambda }^{(4k)}\right) \leqslant \frac{{\gamma }({\Lambda }^{(0)})\exp (O(C^{2})\log ^2(1/q))}{\prod _{i\in [4k]}{\mu }(\mathcal S\mathcal G^\textbf{1}({\Lambda }^{(i+1)})){\mu }(\overline{\mathcal S\mathcal T}({\Lambda }^{(i)}_1)){\mu }(\overline{\mathcal S\mathcal G}({\Lambda }_2^{(i)})){\mu }(\overline{\mathcal S\mathcal T}({\Lambda }^{(i)}_3))}.\nonumber \\ \end{aligned}$$

In view of Proposition 6.2, it suffices to prove that each of the terms in the denominator above is at least $\exp (-C^{O(1)}\log ^2(1/q)/q^{\alpha })$.

Inspecting Definitions  6.3 and 6.5, we see that each $\mathcal S\mathcal G$, $\overline{\mathcal S\mathcal G}$ and $\overline{\mathcal S\mathcal T}$ event in Eq. (35) requires at most $C\ell ^{\textrm{int}}$ fixed infections, $W^{O(1)}$ *W*-helping sets and *O*(1) $(\textbf{1},W)$-symmetrically traversable tubes. We claim that the probability of each tube being $(\textbf{1},W)$-symmetrically traversable is $q^{O(W)}$. Assuming this, the Harris inequality Eq. (7) and the above give that, for all $i\in [4k+1]$,

$$\begin{aligned} {\mu }\left( \mathcal S\mathcal G^\textbf{1}\left( {\Lambda }^{(i)}\right) \right) \geqslant q^{C\ell ^{\textrm{int}}}q^{W^{O(1)}}q^{O(W)}=\exp \left( -C^{O(1)}\log ^2(1/q)/q^{\alpha }\right) \end{aligned}$$

and similarly for the other events.

To prove the claim, let us consider for concreteness and notational convenience the event

$$\begin{aligned} \mathcal E=\mathcal S\mathcal T_W^\textbf{1}\left( T\left( \underline{r}^{(1)},l^{(1)},1\right) \right) , \end{aligned}$$

all tubes being treated identically. As in Eq. (31), applying Definition 4.1, Lemma 4.2, and Observation 3.11, we get

36 $$\begin{aligned} {\mu }(\mathcal E)\geqslant q^{O(W)}\left( 1-e^{-q^{\alpha }\ell ^{\textrm{int}}/O(1)}\right) ^{O(l^{(1)})}\left( 1-e^{-q^W\ell ^{\textrm{mes}-}/O(W)}\right) ^{O(l^{(1)})}. \end{aligned}$$

Here we noted that in directions $i\in (-k+2,k-1)$ symmetric traversability only requires helping sets (since the only hard directions are assumed to be $u_k$ and $u_{-k}$) and the corresponding side lengths of ${\Lambda }^{(1)}$ are $\ell ^{\textrm{int}}+O(1)$, while for $i=k$ it requires *W*-helping sets, but the $u_k$-side of ${\Lambda }^{(1)}$ has length $\ell ^{\textrm{mes}-}+O(1)$. Recalling Sect. 3.4 and the fact that $l^{(1)}=\Theta (\ell ^{\textrm{mes}-})$, Eq. (36) becomes ${\mu }(\mathcal E)\geqslant q^{O(W)}$, as claimed. This concludes the proof of Theorem 6.4.

### CBSEP global dynamics

With Theorem 6.4 established, we are ready to conclude the proof of Theorem 1(d) as in Sect. 5.2.

#### Proof of Theorem 1(d)

Let $\mathcal U$ be unbalanced unrooted. Recall the droplets ${\Lambda }^{(n)}$ from Sect. 6.2. Set ${\Lambda }^{\textrm{mes}+}={\Lambda }^{(4k)}$ and ${\Lambda }^{\textrm{mes}-}={\Lambda }^{(2k)}$. Condition (1) of Proposition 5.8 is satisfied by Theorem 6.4, while condition (2) is verified as in Sect. 5.2.

Thus, Proposition 5.8 applies and, together with Theorem 6.4, it yields

$$\begin{aligned} {\mathbb E} _{{\mu }}[{\tau }_0]\leqslant \exp \left( \frac{\log ^2(1/q)}{{\varepsilon }q^{\alpha }}\right) , \end{aligned}$$

concluding the proof. $\square$

## Semi-directed Models

In this section we aim to treat semi-directed update families $\mathcal U$ (class (f)). The internal dynamics (Sect. 7.1) based on East extensions is the most delicate. The mesoscopic and global dynamics (Sects. 7.2 and 7.3) use the CBSEP mechanism along the same lines as in Sects. 5 and 6.

### East internal dynamics

In view of Remark 1.6, in Sect. 7.1 we work not only with semi-directed models (class (f)), but slightly more generally, in order to also treat balanced rooted models with finite number of stable directions (class (e)), whose update rules are contained in the axes of the lattice (in which case $k=1$—recall Sect. 3.2). In either case we have that ${\alpha }(u_j)\leqslant {\alpha }$ for all $j\in [4k]\setminus \{3k-1,3k\}$ and this is the only assumption on $\mathcal U$ we use.

Recalling Sect. 3.4, set37

#### Remark 7.1

Note that despite the extremely fast divergence of $\ell ^{(n)}q^{\alpha }$, for $n\in (N^{\textrm{cr}},N^{\textrm{int}}]$ it holds that $W\leqslant \ell ^{(n+1)}/\ell ^{(n)}<(\ell ^{(n)}q^{\alpha })^2<\log ^4(1/q)$. The sharp divergence ensures that some error terms below sum to the largest one. This prevents additional factors of the order of $N^{\textrm{int}}-N^{\textrm{cr}}$ in the final answer, particularly for the semi-directed class (f) (recall Sect. 2.4.3). This technique was introduced in [25], Eq. (16)], while the geometrically increasing scale choice relevant for small *n* originates from [16]. It should be noted that this divergence can be further amplified up to a tower of exponentials of height linear in $n-N^{\textrm{cr}}$. In that case the $\log \log \log (1/q)$ error term in Theorem 8.5 and (4) below becomes $\log _*(1/q)$, but is, alas, still divergent.

Recall Sect. 3.3. Let $\underline{r}^{(0)}=(r^{(0)}_j)_{j\in [4k]}$ be a symmetric sequence of radii such that $\underline{r}=\Theta (1/{\varepsilon })$, the vertices of ${\Lambda }(\underline{r}^{(0)})$ are in $2{\mathbb Z} ^2$ and the corresponding side lengths $\underline{s}^{(0)}$ are also $\Theta (1/{\varepsilon })$. For $n\in {\mathbb N}$ and $j\in [4k]$, we define $s_j^{(n)}=s_j^{(0)}\ell ^{(n)}$. We denote ${\Lambda }^{(n)}={\Lambda }(\underline{r}^{(n)})$, where $\underline{r}^{(n)}$ is the sequence of radii corresponding to $\underline{s}^{(n)}$ such that $r^{(n)}_{3k}=r_{3k}^{(0)}$ and $r^{(n)}_{3k-1}=r^{(0)}_{3k-1}$ (see Fig. 6).

For $j\in [2k]$, we write $l^{(n+j/(2k))}=s_{j+k}^{(n+1)}-s_{j+k}^{(n)}=\Theta (\ell ^{(n+1)}/{\varepsilon })$ and set $\underline{r}^{(n+(j+1)/(2k))}=\underline{r}^{(n+j/(2k))}+l^{(n+j/(2k))}\underline{v}_j$, which is consistent with the definition of $\underline{r}^{(n+1)}$ above. Thus, denoting ${\Lambda }^{(n+j/(2k))}={\Lambda }(\underline{r}^{(n+j/(2k))})$ for $n\in {\mathbb N}$ and $j\in (0,2k)$ (see Fig. 6), we may define SG events of these droplets by extension (recall Definition 4.4 and Fig. 2a for East-extensions).

#### Definition 7.2

(*Semi-directed internal SG*). Let $\mathcal U$ be semi-directed or balanced rooted with finite number of stable directions and $k=1$. We say that ${\Lambda }^{(0)}$ is SG ($\mathcal S\mathcal G^\textbf{1}({\Lambda }^{(0)})$ occurs), if all sites in ${\Lambda }^{(0)}$ are infected. We then recursively define $\mathcal S\mathcal G^\textbf{1}({\Lambda }^{(n+(j+1)/(2k))})$, for $n\in [N^{\textrm{int}}]$ and $j\in [2k]$, by East-extending ${\Lambda }^{(n+j/(2k))}$ in direction $u_j$ by $l^{(n+j/(2k))}$ (see Fig. 6).

As usual, we seek to bound the probability of $\mathcal S\mathcal G^\textbf{1}({\Lambda }^{(N^{\textrm{int}})})$ and associated ${\gamma }({\Lambda }^{(N^{\textrm{int}})})$ (recall Sect. 3.6).

#### Theorem 7.3

Let $\mathcal U$ be semi-directed (class (f)) or balanced rooted with finite number of stable directions (class (e)) and $k=1$. Then

$$\begin{aligned} {\gamma }\left( {\Lambda }^{(N^{\textrm{int}})}\right)&{}\leqslant \exp \left( \frac{\log \log (1/q)}{{\varepsilon }^6q^{\alpha }}\right) ,&{\mu }\left( \mathcal S\mathcal G^\textbf{1}\left( {\Lambda }^{(N^{\textrm{int}})}\right) \right)&{}\geqslant \exp \left( \frac{-1}{{\varepsilon }^2q^{\alpha }}\right) . \end{aligned}$$

The rest of Sect. 7.1 is dedicated to the proof of Theorem 7.3. The probability bound is fairly easy, as in Eq. (29), while the relaxation time is bounded by iteratively using Proposition 4.6 and then carefully estimating the product appearing there with the help of Lemma 4.11.

Note that ${\gamma }({\Lambda }^{(0)})=1$, since Eq. (15) is trivial, as $\mathcal S\mathcal G^\textbf{1}({\Lambda }^{(0)})$ is a singleton. For $n\in 1/(2k){\mathbb N}$, $j\in [2k]$ and $m\geqslant 1$, such that $n<N^{\textrm{int}}$ and $n-j/(2k)\in {\mathbb N}$ set

38 $$\begin{aligned} a^{(n)}_{m}={\mu }^{-1}\left( \left. \mathcal S\mathcal G^\textbf{1}\left( {\Lambda }^{(n)}+\left( \left\lfloor (3/2)^{m+1}\right\rfloor -\left\lfloor (3/2)^{m}\right\rfloor \right) {\lambda }_j u_j\right) \right| \mathcal S\mathcal G^\textbf{1}\left( {\Lambda }^{(n)}\right) \right) . \end{aligned}$$

We further let

39 $$\begin{aligned} M^{(n)}=\min \left\{ m:{\lambda }_j(3/2)^{m+1}\geqslant l^{(n)}\right\} =\log l^{(n)}/\log (3/2)+O(1). \end{aligned}$$

For the sake of simplifying expressions we abusively assume that $l^{(n)}={\lambda }_j\lfloor (3/2)^{M^{(n)}+1}\rfloor$. Without this assumption, one would need to treat the term corresponding to $m=M^{(n)}$ below separately, but identically.

We next seek to apply Proposition 4.6 with $\underline{r}=\underline{r}^{(n)}$ and $l=l^{(n)}$. Let us first analyse the term $a_m$ in Eq. (17). By Definition 4.4 and the Harris inequality Eq. (9), we have

40 $$\begin{aligned} a_m\leqslant \frac{a_m^{(n)}}{{\mu }(\mathcal T^\textbf{1}(T+(\lfloor (3/2)^{m+1}\rfloor -\lfloor (3/2)^{m}\rfloor ){\lambda }_ju_j)|\mathcal T^\textbf{1}(T))}=\frac{a_m^{(n)}}{b_m^{(n)}}, \end{aligned}$$

using Lemma 4.3 in the equality and setting

$$\begin{aligned} T&{}=T\left( \underline{r}^{(n)},{\lambda }_j\lfloor (3/2)^m\rfloor ,j\right) \\ b_m^{(n)}&{}={\mu }\left( \mathcal T^\textbf{1}\left( T\left( \underline{r}^{(n)},\left( \left\lfloor (3/2)^{m+1}\right\rfloor -\left\lfloor (3/2)^{m}\right\rfloor \right) {\lambda }_j,j\right) \right) \right) \end{aligned}$$

Moreover, by Lemmas  4.2 and 4.3 we have

41 $$\begin{aligned} \prod _{m=1}^{M^{(n)}}b_m^{(n)}&{}=q^{-O(WM^{(n)})}{\mu }\left( \mathcal T^\textbf{1}\left( T\left( \underline{r}^{(n)},l^{(n)},j\right) \right) \right) \nonumber \\&{}=q^{-O(WM^{(n)})}\frac{{\mu }(\mathcal S\mathcal G^\textbf{1}({\Lambda }^{(n+1/(2k))}))}{{\mu }(\mathcal S\mathcal G^\textbf{1}({\Lambda }^{(n)}))}, \end{aligned}$$

where the second equality uses Definitions  4.4 and 7.2.

Applying Proposition 4.6 successively and using Eqs. (39) and (40), we get

42 $$\begin{aligned} {\gamma }\left( {\Lambda }^{(N^{\textrm{int}})}\right)&{}\leqslant \max _{n\leqslant N^{\textrm{int}}}{\mu }^{-1}\left( \mathcal S\mathcal G^\textbf{1}\left( {\Lambda }^{(n)}\right) \right) \prod _{n=0}^{N^{\textrm{int}}-1/(2k)}e^{O(C^2)\log ^{2}(1/q)}\prod _{m=1}^{M^{(n)}}\frac{a_m^{(n)}}{b_m^{(n)}}\nonumber \\&{}\leqslant \frac{{\mu }(\mathcal S\mathcal G^\textbf{1}({\Lambda }^{(0)}))e^{O(C^2)N^{\textrm{int}}\log ^2(1/q)}}{{\mu }^2(\mathcal S\mathcal G^\textbf{1}({\Lambda }^{(N^{\textrm{int}})}))}\prod _{n=0}^{N^{\textrm{int}}-1/(2k)}q^{-O(WM^{(n)})}\prod _{m=1}^{M^{(n)}}a_m^{(n)}\nonumber \\&{}\leqslant \frac{\exp (\log ^{O(1)}(1/q))}{{\mu }^2(\mathcal S\mathcal G^\textbf{1}({\Lambda }^{(N^{\textrm{int}})}))}\prod _{n=0}^{N^{\textrm{int}}-1/(2k)}\prod _{m=1}^{M^{(n)}}a_m^{(n)}, \end{aligned}$$

where in the second inequality we used Eq. (41) and the fact that ${\mu }(\mathcal S\mathcal G^\textbf{1}({\Lambda }^{(n)}))$ is non-increasing in *n* (recall Definitions 4.4 and 7.2); in the third inequality we used $N^{\textrm{int}}\leqslant \log (1/q)$ by Eq. (37) and $M^{(n)}\leqslant O(\log (1/q))$ by Eq. (37) and (39). Note that in Eq. (42) and below products on *n* run over $1/(2k){\mathbb N}$.

To evaluate the r.h.s. of Eq. (42) we need the following lemma.

#### Lemma 7.4

Let $n\in 1/(2k){\mathbb N}$ be such that $n\leqslant N^{\textrm{int}}$ and $m\geqslant 1$. Then

43 $$\begin{aligned} a_m^{(n)}\leqslant {\mu }^{-1}\left( \mathcal S\mathcal G^\textbf{1}\left( {\Lambda }^{(n)}\right) \right) \leqslant \min \left( ({\delta }q^{\alpha }W^n)^{-W^n/{\varepsilon }^2},e^{1/({\varepsilon }^2q^{\alpha })}\right) . \end{aligned}$$

Moreover, if

44 $$\begin{aligned} \ell ^{(\lfloor n\rfloor )}&{}\geqslant 1/\left( q^{{\alpha }}\log ^W(1/q)\right) ,&M^{(n)}{}&\geqslant m+W,&(3/2)^m&{}\leqslant 1/q^{\alpha }, \end{aligned}$$

setting

45 $$\begin{aligned} n_m=\min \left\{ n'\in {\mathbb N} :\ell ^{(n')}\geqslant 1/\left( q^{\alpha }\log ^W(1/q)\right) ,M^{(n')}\geqslant m+W\right\} \leqslant n, \end{aligned}$$

the following improvements hold

46 $$\begin{aligned} a_m^{(n)}\leqslant {}&\exp \left( \frac{(3/2)^m}{{\varepsilon }^4}\left( (N^{\textrm{cr}}-n_m)^2+{\mathbb {1}} _{n\geqslant N^{\textrm{cr}}}\log ^{2/3}\log (1/q)\right) \right) \nonumber \\&\times {\left\{ \begin{array}{ll} \exp \left( 1/\left( q^{\alpha }\log ^{W-O(1)}(1/q)\right) \right) &{}m\leqslant \frac{\log (1/(q^{\alpha }\log ^W(1/q)))}{\log (3/2)}\\ \exp \left( 1/\left( q^{\alpha }\log ^{W-O(1)}\log (1/q)\right) \right) &{}m> \frac{\log (1/(q^{\alpha }\log ^W(1/q)))}{\log (3/2)}. \end{array}\right. } \end{aligned}$$

Let us finish the proof of Theorem 7.3 before proving Lemma 7.4. The second inequality in Theorem 7.3 is contained in Eq. (43), so we focus on ${\gamma }({\Lambda }^{(N^{\textrm{int}})})$ based on Eq. (42). Set47Using the trivial bound $a_m^{(n)}\leqslant \exp (1/({\varepsilon }^2q^{\alpha }))$ from Eq. (43) and then Eqs. (37) and (39), we get48which is the main contribution. Note that by Eqs. (37) and (39), $n<N^{\textrm{cr}}-1/{\varepsilon }$ implies $M^{(n)}<M_\alpha$, so Eq. (48) exhausts the terms in Eq. (42) with $m\geqslant M_{\alpha }$.

Next set49Using the first bound on $a_m^{(n)}$ from Eqs. (43) and (39), we obtain

50 $$\begin{aligned} \prod _{n=0}^{N_W}\prod _{m=1}^{M^{(n)}}a_m^{(n)}&{}\leqslant \prod _{n=0}^{N_W}({\delta }q^{\alpha }W^n)^{-O(\log (1/q)W^n/{\varepsilon }^2)}\nonumber \\&{}\leqslant \exp \left( -\log ^{O(1)}(1/q)\sum _{n=0}^{N_W}W^n\right) \nonumber \\&{}\leqslant \exp \left( 1/\left( q^{\alpha }\log ^{W-O(1)}(1/q)\right) \right) . \end{aligned}$$

We next turn to the range $N_W\leqslant n<n_m$ with $m<M_\alpha$. Recalling Eqs. (37), (39) and (45), we get that $N_W\leqslant n<n_m$ implies $M^{(n)}<m+W$ and therefore $l^{(n)}\leqslant O((3/2)^{m+W})$, so $W^n\leqslant (3/2)^m$. Plugging this into the first bound on $a_m^{(n)}$ from Eq. (43), we get

51 $$\begin{aligned} \prod ^{M_{\alpha }-1}_{m=1}\prod _{n=N_W}^{n_m-1/(2k)}a_m^{(n)}&{}\leqslant \exp \left( -\sum ^{M_{\alpha }}_{m=1}\frac{(3/2)^m\log ({\delta }q^{\alpha }(3/2)^m)}{{\varepsilon }^3}\right) \leqslant e^{1/(q^{\alpha }{\varepsilon }^4)}. \end{aligned}$$

It remains to treat the range $n_m\leqslant n<N^{\textrm{int}}$ with $m< M_{\alpha }$. Note that by Eqs. (37), (45) and (49) $N_W\leqslant n_m$ for any *m* and set

52 $$\begin{aligned} M_W=\left\lfloor \log \left( 1/\left( q^{\alpha }\log ^W(1/q)\right) \right) /\log (3/2)\right\rfloor . \end{aligned}$$

Then Eq. (46) gives

53 $$\begin{aligned} \sum ^{M_{\alpha }-1}_{m=1}\sum _{n=n_m}^{N^{\textrm{int}}-1/(2k)}\log a_m^{(n)}\leqslant {}&\frac{2k}{{\varepsilon }^4}\sum _{m=1}^{M_{\alpha }-1}(3/2)^m(N^{\textrm{cr}}-n_m)^2(N^{\textrm{int}}-N^{\textrm{cr}}+N^{\textrm{cr}}-n_m)\nonumber \\&+\frac{2k}{{\varepsilon }^4}(N^{\textrm{int}}-N^{\textrm{cr}})\log ^{2/3}\log (1/q)\sum _{m=1}^{M_{\alpha }-1}(3/2)^m\nonumber \\&+\frac{2kM_{\alpha }N^{\textrm{int}}}{q^{\alpha }\log ^{W-O(1)}(1/q)}+\frac{2k(M_{\alpha }-M_W)(N^{\textrm{int}}-N_W)}{q^{\alpha }\log ^{W-O(1)}\log (1/q)}\nonumber \\ \leqslant {}&\frac{8k}{{\varepsilon }^4}\log \log \log (1/q)\sum _{m=1}^{M_{\alpha }-1}(3/2)^m(N^{\textrm{cr}}-n_m)^3\nonumber \\&+\frac{\log ^{2/3}\log (1/q)\log \log \log (1/q)}{{\varepsilon }^5q^{\alpha }}\nonumber \\&+\frac{1}{q^{\alpha }\log ^{W-O(1)}(1/q)}+\frac{1}{q^{\alpha }\log ^{W-O(1)}\log (1/q)}, \end{aligned}$$

where we used that $N^{\textrm{int}}-N^{\textrm{cr}}\leqslant 2\log \log \log (1/q)$ by Eq. (37), $M_{\alpha }\leqslant \log ^{O(1)}(1/q)$ by Eq. (47), $N^{\textrm{int}}\leqslant \log ^{O(1)}(1/q)$ by (37), $M_{\alpha }-M_W\leqslant \log ^{O(1)}\log (1/q)$ by Eqs. (47) and (52) and $N^{\textrm{int}}-N_W\leqslant \log ^{O(1)}\log (1/q)$ by Eqs. (37) and (49). In order to bound the last sum in Eq. (53), we note that by Eqs. (37), (45), (47), and (52), for any $m\in [M_W,M_{\alpha })$ we have $N^{\textrm{cr}}-n_m\leqslant (M_{\alpha }-m)/{\varepsilon }$. Plugging this back into Eq. (53), we get

$$\begin{aligned} \sum ^{M_{\alpha }-1}_{m=1}\sum _{n=n_m}^{N^{\textrm{int}}-1/(2k)}\log a_m^{(n)}\leqslant {}&\frac{\log \log \log (1/q)}{{\varepsilon }^{O(1)}}\left( M_{\alpha }^4(3/2)^{M_W}+(3/2)^{M_{\alpha }}\right) \\&+\frac{\log ^{3/4}\log (1/q)}{2q^{\alpha }}\\ \leqslant {}&\frac{\log ^{3/4}\log (1/q)}{q^{\alpha }}. \end{aligned}$$

Plugging the last result and Eqs. (48), (50) and (51) in Eq. (42), we conclude the proof of Theorem 7.3, since ${\mu }(\mathcal S\mathcal G^\textbf{1}({\Lambda }^{(N^{\textrm{int}})}))\geqslant e^{-1/({\varepsilon }^2q^{\alpha })}$ by Eq. (43).

#### Proof of Lemma 7.4

Let us fix *m* and *n* as in the statement for Eq. (43). The bound $a_m^{(n)}\leqslant {\mu }^{-1}(\mathcal S\mathcal G^\textbf{1}({\Lambda }^{(n)}))$ follows from the Harris inequality Eq. (8). To upper bound the latter term we note that by Definitions 4.4 and 7.2,

54 $$\begin{aligned} {\mu }\left( \mathcal S\mathcal G^\textbf{1}\left( {\Lambda }^{(n)}\right) \right) ={\mu }\left( \mathcal S\mathcal G^\textbf{1}\left( {\Lambda }^{(0)}\right) \right) \prod _{p=0}^{n-1/(2k)}{\mu }\left( \mathcal T^\textbf{1}\left( T\left( \underline{r}^{(p)},l^{(p)},j(p)\right) \right) \right) , \end{aligned}$$

setting $j(p)\in [2k]$ such that $p-j(p)/(2k)\in {\mathbb N}$ and letting products on *p* run over $1/(2k){\mathbb N}$. Clearly,

55 $$\begin{aligned} {\mu }\left( \mathcal S\mathcal G^\textbf{1}\left( {\Lambda }^{(0)}\right) \right) =q^{|{\Lambda }^{(0)}|}=q^{\Theta (1/{\varepsilon }^2)}. \end{aligned}$$

Let us fix $p\in 1/(2k){\mathbb N}$, $p<N^{\textrm{int}}$. Then, using Lemma 4.2, Definition 4.1, Observation 3.11, and the Harris inequality Eq. (7), we get

56 $$\begin{aligned}{} & {} {\mu }\left( \mathcal T^\textbf{1}\left( T\left( \underline{r}^{(p)},l^{(p)},j(p)\right) \right) \right) \nonumber \\{} & {} \quad \geqslant {} q^{O(W)}\left( 1-e^{-q^{\alpha }\ell ^{(\lfloor p\rfloor )}/O({\varepsilon })}\right) ^{O(l^{(p)})}\nonumber \\{} & {} \quad \geqslant {}q^{O(W)} {\left\{ \begin{array}{ll} ({\delta }q^{\alpha }W^p)^{W^p/({\delta }{\varepsilon })}&{}p\leqslant N^{\textrm{cr}},\\ \exp \left( -1/\left( q^{{\alpha }}\exp \left( W^{\exp (\lfloor p\rfloor -N^{\textrm{cr}})}/{\delta }\right) \right) \right) &{}p> N^{\textrm{cr}}.\end{array}\right. } \end{aligned}$$

In the last inequality we took into account $1/{\varepsilon }\gg 1/{\delta }\gg W\gg 1$, $\ell ^{(N^{\textrm{cr}})}=W^{O(1)}/q^{\alpha }$ and the explicit expressions Eq. (37). From Eqs. (54)–(56) it is not hard to check Eq. (43) (recalling Sect. 3.4).

We next turn to proving Eq. (46), so we fix $n\leqslant N^{\textrm{int}}$ and $m\geqslant 1$ satisfying Eq. (44). Denote $s_m=(\lfloor (3/2)^{m+1}\rfloor -\lfloor (3/2)^{m}\rfloor ){\lambda }_j u_j$ for $j=j(n)$, so that Eq. (38) spells

$$\begin{aligned} a_m^{(n)}={\mu }^{-1}\left( \left. \mathcal S\mathcal G^\textbf{1}\left( {\Lambda }^{(n)}+s_m\right) \right| \mathcal S\mathcal G^\textbf{1}\left( {\Lambda }^{(n)}\right) \right) . \end{aligned}$$

By the Harris inequality, Eqs. (8) and (9), Definitions 4.4 and 7.2 we have

57 $$\begin{aligned} a_m^{(n)}\leqslant {}&{\mu }^{-1}\left( \mathcal S\mathcal G^\textbf{1}\left( {\Lambda }^{(n_m)}\right) \right) \nonumber \\&\times \prod _{p=n_m}^{n-1/(2k)}{\mu }^{-1}\left( \left. \mathcal T^\textbf{1}\left( T\left( \underline{r}^{(p)},l^{(p)},j(p)\right) +s_m\right) \right| \mathcal T^\textbf{1}\left( T\left( \underline{r}^{(p)},l^{(p)},j(p)\right) \right) \right) . \end{aligned}$$

Our goal is then to bound the last factor, using Lemma 4.11, which quantifies the fact that “small perturbations $s_m$ do not modify traversability much.”

Let us fix $p\in [n_m,n)\cap (1/2k){\mathbb N}$ and denote

$$\begin{aligned} \mathcal T&{}=\mathcal T^\textbf{1}\left( T\left( \underline{r}^{(p)},l^{(p)},j(p)\right) \right)&\mathcal T'&{}=\mathcal T^\textbf{1}\left( T\left( \underline{r}^{(p)},l^{(p)},j(p)\right) +s_m\right) . \end{aligned}$$

In order to apply Lemma 4.11 with ${\Delta }=\max (C^2,\Vert s_m\Vert )$, we check that $W^3 (3/2)^m\leqslant \ell ^{(\lfloor p\rfloor )}/{\varepsilon }$ (so that the sides of ${\Lambda }^{(p)}$ are large enough). If $\ell ^{(\lfloor p\rfloor )}\geqslant 1/q^{\alpha }$, this follows from the assumption $(3/2)^m\leqslant 1/q^{\alpha }$ of Lemma 7.4. Otherwise, by Eqs. (39) and (45)

$$\begin{aligned} W^3(3/2)^m&{}\leqslant (3/2)^{M^{(n_m)}-W/2}\leqslant l^{(n_m)}/e^{{\Omega }(W)}=\Theta \left( \ell ^{(n_m+1)}\right) /\left( {\varepsilon }e^{{\Omega }(W)}\right) \\&{}\leqslant \ell ^{(n_m)}/{\varepsilon }\leqslant \ell ^{(\lfloor p\rfloor )}/{\varepsilon }, \end{aligned}$$

where in the last but one inequality we used that $\ell ^{(n_m+1)}\leqslant W^{O(1)}\ell ^{(n_m)}$, since $n_m\leqslant p$ and $\ell ^{(p)}\leqslant 1/q^{\alpha }$ (recall Eq. (37)). The remaining hypotheses of Lemma 4.11 are immediate to verify.

For $\Vert s_m\Vert =\Theta ((3/2)^m)\leqslant C^2$, Lemma 4.11 gives

$$\begin{aligned} {\mu }\left( \left. \mathcal T'\right| \mathcal T\right) \geqslant q^{O(C^2)}\left( 1-q^{1-o(1)}\right) ^{O(l^{(p)})}\geqslant \exp \left( -q^{-{\alpha }+1/2}\right) , \end{aligned}$$

as $l^{(p)}\leqslant \ell ^{(N^{\textrm{int}})}/{\varepsilon }\leqslant q^{-{\alpha }-o(1)}$. If, on the contrary, $\Vert s_m\Vert \geqslant C^2$, Lemma 4.11 gives

58 $$\begin{aligned} {\mu }(\mathcal T'|\mathcal T)\geqslant {}&q^{O(W)}\times \left( 1-(1-q^{\alpha })^{\ell ^{(\lfloor p\rfloor )}/O({\varepsilon })}\right) ^{O((3/2)^m)}\nonumber \\&\times \left( 1-O(W{\varepsilon })(3/2)^m/\ell ^{(\lfloor p\rfloor )}-q^{1-o(1)}\right) ^{O(\ell ^{(\lfloor p\rfloor +1)}/{\varepsilon })} \nonumber \\ \geqslant {}&q^{O(W)}\times {\left\{ \begin{array}{ll}({\delta }q^{\alpha }W^p)^{O((3/2)^m)}&{}p\leqslant N^{\textrm{cr}}\\ \exp \left( -(3/2)^m\exp \left( -W^{\exp (\lfloor p\rfloor -N^{\textrm{cr}})}/{\delta }\right) \right) &{}p>N^{\textrm{cr}}\end{array}\right. }\nonumber \\&\times {\left\{ \begin{array}{ll} \exp \left( -q^{-{\alpha }+1/2-o(1)}\right) &{}(3/2)^m\leqslant q^{-{\alpha }+1/2-o(1)}\\ \exp \left( -W^2(3/2)^m\frac{\ell ^{(\lfloor p\rfloor +1)}}{\ell ^{(\lfloor p\rfloor )}}\right) &{}(3/2)^m> q^{-{\alpha }+1/2-o(1)}, \end{array}\right. } \end{aligned}$$

in view of Eq. (37). Further notice that if $(3/2)^m\leqslant q^{-{\alpha }+1/2-o(1)}$ or $p>N^{\textrm{cr}}$, the third term dominates, while otherwise the second one does. Moreover, if  with

59 $$\begin{aligned} \Psi =\log \frac{\log \log \log (1/q)}{3\log W}, \end{aligned}$$

then the Harris inequality Eq. (8), translation invariance and Eq. (56) directly give the bound

60 $$\begin{aligned} {\mu }(\mathcal T'|\mathcal T)\geqslant {\mu }(\mathcal T')={\mu }(\mathcal T)\geqslant \exp \left( -1/\left( q^{\alpha }\log ^W\log (1/q)\right) \right) . \end{aligned}$$

Finally, we can plug Eqs. (43), (58) and (60) in Eq. (57) to obtain the following bounds. If $(3/2)^m\leqslant q^{-{\alpha }+1/2-o(1)}$, then

$$\begin{aligned} a_m^{(n)}\leqslant \exp \left( 1/\left( q^{\alpha }\log ^{W}(1/q)\right) \right) , \end{aligned}$$

because the contribution from Eq. (58) is negligible, since $n\leqslant N^{\textrm{int}}\leqslant \log (1/q)$, while by Eqs. (37) and (45), $W^{n_m}=\ell ^{(n_m)}\leqslant W/(q^{\alpha }\log ^W(1/q))$. If, on the contrary, $(3/2)^m>q^{-{\alpha }+1/2-o(1)}$, then

$$\begin{aligned} a_m^{(n)}\leqslant {}&{\left\{ \begin{array}{ll} \exp \left( 1/\left( q^{\alpha }\log ^{W-O(1)}(1/q)\right) \right) &{}(3/2)^m\leqslant 1/\left( q^{\alpha }\log ^W(1/q)\right) \\ \left( {\delta }q^{\alpha }W^{n_m}\right) ^{-(3/2)^m/{\varepsilon }^3}&{}(3/2)^m> 1/\left( q^{\alpha }\log ^W(1/q)\right) \end{array}\right. }\\&\times \prod _{p=n_m}^{\min (n,N^{\textrm{cr}})}({\delta }q^{\alpha }W^p)^{-O((3/2)^m)}\\&{}\times {\left\{ \begin{array}{ll} 1&{}n\leqslant N^{\textrm{cr}}\\ \exp \left( (3/2)^mW^{2\exp (\Psi )}/{\delta }\right) &{}n>N^{\textrm{cr}}\end{array}\right. }\\&\times {\left\{ \begin{array}{ll} \exp \left( 1/\left( q^{\alpha }\log ^{W-O(1)}(1/q)\right) \right) &{}(3/2)^m\leqslant 1/\left( q^{\alpha }\log ^W(1/q)\right) \\ \exp \left( 1/\left( q^{\alpha }\log ^{W-O(1)}\log (1/q)\right) \right) &{}(3/2)^m> 1/\left( q^{\alpha }\log ^W(1/q)\right) , \end{array}\right. } \end{aligned}$$

the terms corresponding to ${\mu }^{-1}(\mathcal S\mathcal G^\textbf{1}({\Lambda }^{(n_m)}))$ and to values of *p* in the intervals $[n_m,N^{\textrm{cr}}]$,  and  respectively. Indeed, in the last term for small *m* we used Eq. (58), while for large *m*, we directly applied Eq. (60). Observing that the product of the second case for the first term, the second term and the third term can be bounded by

$$\begin{aligned} \exp \left( \frac{(3/2)^m}{{\varepsilon }^4}\left( (N^{\textrm{cr}}-n_m)^2 +{\mathbb {1}} _{n\geqslant N^{\textrm{cr}}}\log ^{2/3}\log (1/q)\right) \right) , \end{aligned}$$

we obtain the desired Eq. (46). $\square$

### CBSEP mesoscopic dynamics

In this section we assume that $\mathcal U$ is semi-directed (class (f)) and w.l.o.g. ${\alpha }(u_i)\leqslant {\alpha }$ for all $i\in [4k]\setminus \{3k\}$. The approach to the mesoscopic dynamics is very similar to the one of Sect. 6.2, employing a bounded number of CBSEP-extensions to go from the internal to the mesoscopic scale. Once again, the geometry of our droplets is as in Fig. 4a, but extensions are much longer so that we go from scale $\ell ^{\textrm{int}}$ to $\ell ^{\textrm{mes}-}$ in 2*k* extensions and then to $\ell ^{\textrm{mes}+}$ in another 2*k* extensions.

Recall from Sect. 7.1 that we defined ${\Lambda }^{(N^{\textrm{int}})}$, a symmetric droplet with side lengths $\underline{s}^{(N^{\textrm{int}})}$ equal to $\Theta (\ell ^{(N^{\textrm{int}})}/{\varepsilon })$, as well as $\mathcal S\mathcal G^\textbf{1}({\Lambda }^{(N^{\textrm{int}})})$ in Definition 7.2. Further recall Sect. 3.4. Following Sect. 6.2, for $i\in [1,2k]$ we definewhile for $i\in (2k,4k]$, we set61We then define ${\Lambda }^{(N^{\textrm{int}}+i)}={\Lambda }(\underline{r}^{(N^{\textrm{int}}+i)})$ with $\underline{r}^{(N^{\textrm{int}}+i)}$ the sequence of radii associated to $\underline{s}^{(N^{\textrm{int}}+i)}$ satisfying

$$\begin{aligned} {\Lambda }\left( \underline{r}^{(N_i+i)}\right)&{}={\Lambda }\left( \underline{r}^{(N^{\textrm{int}}+i-1)}+l^{(N^{\textrm{int}}+i-1)}\left( \underline{v}_{i-1}+\underline{v}_{i+2k-1}\right) /2\right) ,\\ l^{(N^{\textrm{int}}+i-1)}&{}=s^{(N^{\textrm{int}}+i)}_{i+k-1}-s^{(N^{\textrm{int}}+i-1)}_{i+k-1}={\left\{ \begin{array}{ll}(1-o(1))\ell ^{\textrm{mes}-}&{}i\in [1,2k],\\ (1-O({\delta }))\ell ^{\textrm{mes}+}&{}i\in (2k,4k].\end{array}\right. } \end{aligned}$$

We then define the corresponding SG events by CBSEP-extension as in Definition 6.3.

#### Definition 7.5

(*Semi-directed mesoscopic SG*). Let $\mathcal U$ be semi-directed. For $i\in [4k]$ we define $\mathcal S\mathcal G^\textbf{1}({\Lambda }^{(N^{\textrm{int}}+i+1)})$ by CBSEP-extending ${\Lambda }^{(N^{\textrm{int}}+i)}$ by $l^{(N^{\textrm{int}}+i)}$ in direction $u_i$.

We then turn to bounding ${\gamma }({\Lambda }^{(N^{\textrm{int}}+4k)})$ (recall Sect. 3.6).

#### Theorem 7.6

Let $\mathcal U$ be semi-directed (class (f)). Then

$$\begin{aligned} {\gamma }\left( {\Lambda }^{(N^{\textrm{int}}+4k)}\right)&{}\leqslant \exp \left( \frac{\log \log (1/q)}{{\varepsilon }^{O(1)}q^{\alpha }}\right) ,\\ {\mu }\left( \mathcal S\mathcal G^\textbf{1}\left( {\Lambda }^{(N^{\textrm{int}}+2k)}\right) \right)&{}\geqslant \exp \left( \frac{-1}{{\varepsilon }^{O(1)}q^{\alpha }}\right) . \end{aligned}$$

The rest of Sect. 7.2 is dedicated to the proof of Theorem 7.6. The proof proceeds exactly like Theorem 6.4, except that the first two steps are much more delicate. Namely, they require taking into account the internal structure of $\mathcal S\mathcal G^\textbf{1}({\Lambda }^{(N^{\textrm{int}})})$ on all scales down to 0. This structure is, alas, rather complex (recall Fig. 6) and also not symmetric w.r.t. the reflection interchanging $u_0$ and $u_{2k}$. This is not unexpected and is, to some extent, the crux of semi-directed models.

**Fig. 7:**
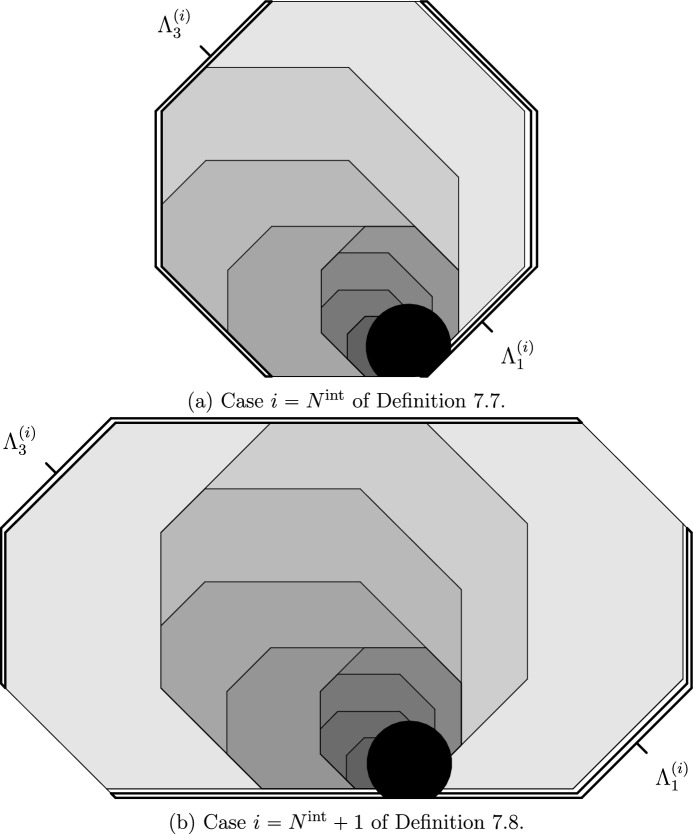
The events $\overline{\mathcal S\mathcal T}({\Lambda }_1^{(i)})$, $\overline{\mathcal S\mathcal G}({\Lambda }^{(i)}_2)$ and $\overline{\mathcal S\mathcal T}({\Lambda }_3^{(i)})$. The microscopic black regions are entirely infected. Shaded tubes are $(\textbf{1},W)$-traversable. *W*-helping sets are required close to all boundaries

As before, we define ${\Lambda }_1^{(i)},{\Lambda }_2^{(i)},{\Lambda }_3^{(i)}$ by Eq. (20) for $i\in [N^{\textrm{int}},N^{\textrm{int}}+4k)$. The next definitions are illustrated in Fig. 7 and are the analogue of Definition 6.5, but taking into account Definition 7.2. Correspondingly, the intuition behind them is the same, the only difference being that we need to modify traversability events at all scales, because ${\Lambda }^{(i)}$ touches the boundary of ${\Lambda }^{(N^{\textrm{int}})}$ for all $i\leqslant N^{\textrm{int}}$ (compare Figs. 4a and 6).

#### Definition 7.7

(*Contracted semi-directed events on scale* $N^{\textrm{int}}$). Let us define $\overline{\mathcal S\mathcal T}({\Lambda }_3^{(N^{\textrm{int}})})$ to be the event that for all $j\in [-k+1,k-1]$ and, for every segment $S\subset {\Lambda }_3^{(N^{\textrm{int}})}$, perpendicular to $u_j$ of length $s^{(N^{\textrm{int}})}_{j}/W$, the event $\mathcal H^W(S)$ occurs.

Let $\overline{\mathcal S\mathcal T}({\Lambda }_1^{(N^{\textrm{int}})})$ be the event that for all $j\in [k+1,3k-2]$ every segment $S\subset {\Lambda }_1^{(N^{\textrm{int}})}$, perpendicular to $u_j$ of length $s^{(N^{\textrm{int}})}_j/W$, the event $\mathcal H^W(S)$ occurs and all sites in ${\Lambda }_1^{(N^{\textrm{int}})}$ at distance at most $\sqrt{W}/{\varepsilon }$ from the origin are infected.

For $n\in [0,N^{\textrm{int}}]$ such that $2kn\in {\mathbb N}$ let ${\Lambda }'^{(n)}={\Lambda }(\underline{r}^{(n)}-{\lambda }_0(\underline{v}_0+\underline{v}_{2k}))$. Define $\mathcal S\mathcal G'({\Lambda }'^{(n)})$ recursively exactly like $\mathcal S\mathcal G^\textbf{1}({\Lambda }^{(n)})$ in Definition 7.2 with all droplets replaced by their contracted versions ${\Lambda }'$ and all traversability events required in East-extensions (see Definition 4.4) replaced by the corresponding $(\textbf{1},W)$-traversability events^5^ ($\mathcal T^\textbf{1}_W$, see Definition 4.1). Let $\mathcal W'$ be the event that for every $n\in [0,N^{\textrm{int}}]$, $j\in [4k]$ and segment $S\subset {\Lambda }^{(N^{\textrm{int}})}_2$, perpendicular to $u_j$ of length $s_j^{(n)}/W$ at distance at most *W* from the $u_j$-side of ${\Lambda }^{(n)}$, the event $\mathcal H^W(S)$ holds. Let $\mathcal I'$ be the event that all sites in ${\Lambda }^{(N^{\textrm{int}})}_2$ at distance at most $\sqrt{W}/{\varepsilon }$ from the origin are infected. Finally, set

$$\begin{aligned} \overline{\mathcal S\mathcal G}\left( {\Lambda }_2^{(N^{\textrm{int}})}\right) =\mathcal S\mathcal G'\left( {\Lambda }'^{(N^{\textrm{int}})}\right) \cap \mathcal W'\cap \mathcal I'. \end{aligned}$$

#### Definition 7.8

(*Contracted semi-directed events on scale* $N^{\textrm{int}}+1$). We define $\overline{\mathcal S\mathcal T}({\Lambda }_1^{(N^{\textrm{int}}+1)})$ to be the event that for all $j\in [k+2,3k-1]$ and every segment $S\subset {\Lambda }_1^{(N^{\textrm{int}}+1)}$, perpendicular to $u_j$ of length $s_j^{(N^{\textrm{int}})}/W$, the event $\mathcal H^W(S)$ occurs and all sites in ${\Lambda }_1^{(N^{\textrm{int}}+1)}$ at distance at most $\sqrt{W}/{\varepsilon }$ from the origin are infected.

Let $\overline{\mathcal S\mathcal T}({\Lambda }_3^{(N^{\textrm{int}}+1)})$ be the event that for all $j\in [4k]$, $m\in \{N^{\textrm{int}},N^{\textrm{int}}+1\}$ and every segment $S\subset {\Lambda }_3^{(N^{\textrm{int}}+1)}$, perpendicular to $u_j$ of length $s_j^{(m)}/W$ at distance at most *W* from the $u_j$-side of ${\Lambda }^{(m)}$, the event $\mathcal H^W(S)$ occurs.

For $n\in [0,N^{\textrm{int}}]$ such that $2k n\in {\mathbb N}$ let

$$\begin{aligned} {\Lambda }''^{(n)}={\Lambda }\left( \underline{r}''^{(n)}\right) ={\Lambda }\left( \underline{r}^{(n)}-{\lambda }_1\left( \underline{v}_1+\underline{v}_{2k+1}\right) \right) \end{aligned}$$

and define $\mathcal S\mathcal G''({\Lambda }''^{(n)})$ like $\mathcal S\mathcal G'({\Lambda }'^{(n)})$ in Definition 7.7. Further let

$$\begin{aligned} \mathcal S\mathcal G''\left( {\Lambda }''^{(N^{\textrm{int}}+1)}\right) =\mathcal S\mathcal G''\left( {\Lambda }''^{(N^{\textrm{int}})}\right) \cap \bigcap _{j\in \{0,2k\}}\mathcal S\mathcal T^\textbf{1}_W\left( T\left( \underline{r}''^{(N^{\textrm{int}})},l^{(N^{\textrm{int}})}/2,j\right) \right) . \end{aligned}$$

Let $\mathcal W''$ (resp. $\mathcal I''$) be defined like $\mathcal W'$ (resp. $\mathcal I'$) in Definition 7.7 with ${\Lambda }'$ replaced by ${\Lambda }''$ and $N^{\textrm{int}}$ replaced by $N^{\textrm{int}}+1$. Finally, we set

$$\begin{aligned} \overline{\mathcal S\mathcal G}\left( {\Lambda }_2^{(N^{\textrm{int}}+1)}\right) =\mathcal S\mathcal G''\left( {\Lambda }''^{(N^{\textrm{int}}+1)}\right) \cap \mathcal W''\cap \mathcal I''. \end{aligned}$$

Notice that Definition 6.5 for $i\in [2,4k)$ does not inspect the internal structure of $\mathcal S\mathcal G^\textbf{1}({\Lambda }^{(0)})$ (see Fig. 5b). Thus, we may use the exact same definition for $\overline{\mathcal S\mathcal T}({\Lambda }_1^{(N^{\textrm{int}}+i)})$, $\overline{\mathcal S\mathcal G}({\Lambda }_2^{(N^{\textrm{int}}+i)})$ and $\overline{\mathcal S\mathcal T}({\Lambda }_3^{(N^{\textrm{int}}+i)})$ with $i\in [2,4k)$.

We may now turn to the analogue of Lemma 6.6.

#### Lemma 7.9

For all $n\in [N^{\textrm{int}},N^{\textrm{int}}+4k)$ we have $\overline{\mathcal S\mathcal G}({\Lambda }_2^{(n)})\times \overline{\mathcal S\mathcal T}({\Lambda }_3^{(n)})\subset \mathcal S\mathcal G^\textbf{1}({\Lambda }_2^{(n)}\cup {\Lambda }_3^{(n)})$ and similarly for ${\Lambda }_1^{(n)}$ instead of ${\Lambda }_3^{(n)}$.

#### Proof

For $n\geqslant N^{\textrm{int}}+2$ the proof is the same as in Lemmas 5.4 and 6.6.

Assume that $\overline{\mathcal S\mathcal G}({\Lambda }_2^{(N^{\textrm{int}})})$ and $\overline{\mathcal S\mathcal T}({\Lambda }_3^{(N^{\textrm{int}})})$ occur. We seek to prove by induction that for all $n\leqslant N^{\textrm{int}}$ the event $\mathcal S\mathcal G^\textbf{1}({\Lambda }^{(n)})$ occurs. For $n=0$ this is true, since $\mathcal I'$ and the corresponding part of $\overline{\mathcal S\mathcal T}({\Lambda }_3^{(N^{\textrm{int}})})$ in Definition 7.7 give that ${\Lambda }^{(0)}$ is fully infected. By Definition 4.4 and 7.2, it remains to show that for all $n<N^{\textrm{int}}$ the event $\mathcal T=\mathcal T^\textbf{1}(T(\underline{r}^{(n)},l^{(n)},j))$ occurs, where $j\in [4k]$ is such that $n-j/(2k)\in {\mathbb N}$. But by Definition 7.7 the corresponding event $\mathcal T'=\mathcal T^\textbf{1}_W(T(\underline{r}'^{(n)},l^{(n)},j)$ occurs, where ${\Lambda }'^{(n)}={\Lambda }(\underline{r}'^{(n)})$. It therefore remains to observe that $\mathcal W'$, the *W*-helping sets in the definition of $\overline{\mathcal S\mathcal T}({\Lambda }_3^{(N^{\textrm{int}})})$ and $\mathcal T'$ imply $\mathcal T$. Indeed, *W*-helping sets ensure the occurrence of $\mathcal H^\textbf{1}_{C^2}(S)$ for the first and last $\Theta (W)$ segments *S* in Definition 4.1 for $\mathcal T$, while the remaining ones are provided by $\mathcal T'$, since $\underline{r}'^{(n)}$ and $\underline{r}^{(n)}$ only differ by $O(1)\ll W$. We omit the details, which are very similar to those in the proof of Lemma 6.6 (see Fig. 7a).

The remaining three cases (${\Lambda }_1^{(N^{\textrm{int}})}$ instead of ${\Lambda }_3^{(N^{\textrm{int}})}$ and/or $N^{\textrm{int}}+1$ instead of $N^{\textrm{int}}$) are treated analogously (see Fig. 7). $\square$

By Lemma 7.9, Eq. (18) holds, so we may apply Proposition 4.9. Together with the Harris inequality, Eqs. (7) and (8), this gives

62 $$\begin{aligned}{} & {} {\gamma }\left( {\Lambda }^{(N^{\textrm{int}}+4k)}\right) \nonumber \\{} & {} \quad \leqslant \frac{{\gamma }({\Lambda }^{(N^{\textrm{int}})})\exp (O(C^2)\log ^2(1/q))}{\displaystyle \prod _{i=N^{\textrm{int}}}^{N^{\textrm{int}}+4k-1}{\mu }(\mathcal S\mathcal G^\textbf{1}({\Lambda }^{(i+1)})){\mu }(\overline{\mathcal S\mathcal T}({\Lambda }^{(i)}_1)){\mu }(\overline{\mathcal S\mathcal G}({\Lambda }_2^{(i)})){\mu }(\overline{\mathcal S\mathcal T}({\Lambda }^{(i)}_3))}. \end{aligned}$$

In view of Theorem 7.3, it remains to bound each of the terms in the denominator by $\exp (-1/({\varepsilon }^{O(1)}q^{\alpha }))$ in order to conclude the proof of Theorem 7.6.

Notice that a total of ${\varepsilon }^{-O(1)}$ fixed infections and $W^{O(1)}N^{\textrm{int}}=q^{o(1)}$ *W*-helping sets are required in all the events in Eq. (62). This amounts to a negligible factor. The probability of $\mathcal S\mathcal G'({\Lambda }'^{(N^{\textrm{int}})})$ and $\mathcal S\mathcal G''({\Lambda }''^{(N^{\textrm{int}})})$ can be bounded exactly like $\mathcal S\mathcal G^\textbf{1}({\Lambda }^{(N^{\textrm{int}})})$ in Lemma 7.4. This yields a contribution of $\exp (1/({\varepsilon }^{O(1)}q^{\alpha }))$. Finally, the remaining bounded number of $\mathcal S\mathcal T^\textbf{1}_W$ events are treated as in Theorem 6.4 to give a negligible $q^{-O(W)}$ factor. Hence, the proof of Theorem 7.6 is complete.

### Global CBSEP dynamics

The global dynamics is also based on the CBSEP mechanism and proceeds as in Sects. 5.2 and 6.3

#### Proof of 1(f)

Let $\mathcal U$ be semi-directed. Recall the droplets ${\Lambda }^{(N^{\textrm{int}}+i)}$ for $i\in [4k+1]$ from Sect. 7.2. Set ${\Lambda }^{\textrm{mes}+}={\Lambda }^{(N^{\textrm{int}}+4k)}$ and ${\Lambda }^{\textrm{mes}-}={\Lambda }^{(N^{\textrm{int}}+2k)}$. Condition (1) of Proposition 5.8 is satisfied by Theorem 7.6, while condition (2) is verified as in Sect. 5.2.

Thus, Proposition 5.8 applies and, together with Theorem 7.6 it yields

$$\begin{aligned} {\mathbb E} _{{\mu }}[{\tau }_0]\leqslant \exp \left( \frac{\log \log (1/q)}{{\varepsilon }^{O(1)} q^{\alpha }}\right) , \end{aligned}$$

concluding the proof. $\square$

## Balanced Rooted Models with Finite Number of Stable Directions

In this section we deal with balanced rooted models with finite number of stable directions (class (e)). The internal dynamics (Sect. 8.1) uses a two-dimensional version of East-extensions. As usual, it requires the most work, but applies directly also to balanced models with infinite number of stable directions (class (b)). The mesoscopic and global dynamics are imported from [22] in Sect. 8.2.

### East internal dynamics

In this section we simultaneously treat balanced rooted models (classes (b) and (e)). We may therefore assume that ${\alpha }(u_j)\leqslant {\alpha }$ for all $j\in [-k+1,k]$ and this is the only assumption on $\mathcal U$ we use.

Let us start by motivating the coming two-dimensional East-extension we need. By the above assumption on the difficulties, we are allowed to use East-extensions in directions $u_0$ and $u_1$. Indeed, recalling Definition 4.4, we see that for these directions the traversability events (recall Definition 4.1) only require helping sets and not *W*-helping sets. In principle, one could alternate East-extensions in these two directions similarly to what we did e.g. in Sect. 7.1 for directions $u_0,\dots ,u_{2k-1}$. However, this would not work, because extensions in directions $u_0$ and $u_1$ only increase the length of the sides parallel to $u_0$ and $u_1$, while all others remain unchanged (see Fig. 2a). Thus, the traversability events would be too unlikely, since they would require helping sets also for the other sides, e.g. the one with outer normal $u_{2-k}$, which are too small. This would make the probability of the SG event too large. Notice that this issue does not arise when $k=1$, as we saw in Sect. 7.1.

**Fig. 8:**
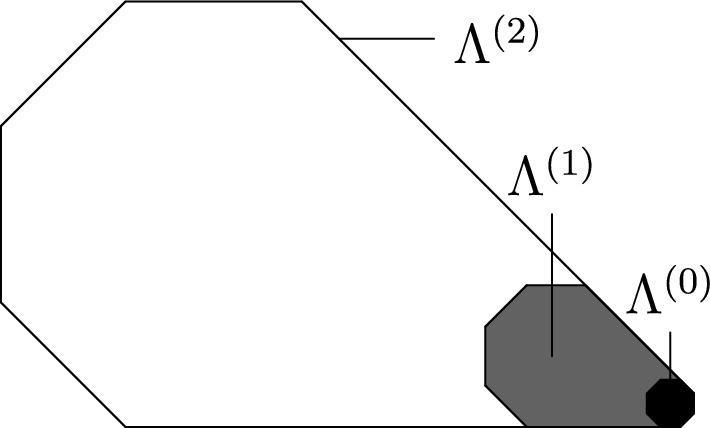
Geometry of the droplets used for balanced rooted models in Sect. 8.1 in the case $k=2$. The nested black, grey and white polygons are the droplets ${\Lambda }^{(0)}$, ${\Lambda }^{(1)}$ and ${\Lambda }^{(2)}$ respectively

**Fig. 9:**
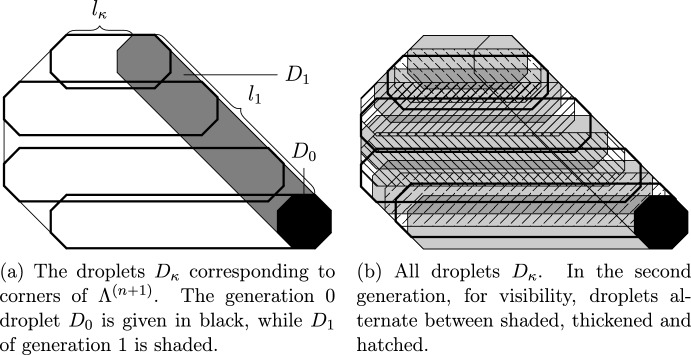
Geometry of the droplets $(D_{\kappa })_{{\kappa }\in [K]}$ used in the two-dimensional East-extension in Definition 8.3. Also recall Fig. 8

For $k>1$, however, we therefore need to make the $u_j$-sides of our successive droplets grow for all $j\in [-k+1,k]$. A natural way to achieve this is as depicted in Fig. 8. The drawback is that we can no longer achieve this directly with one-directional East-extensions as in Definition 4.4 and Fig. 2a, so we need some more definitions. However, morally, one such two-dimensional extension can be achieved by two East-extensions in the sense that, East-extending in direction $u_0$ and then $u_1$ yields a droplet which contains the desired droplet as in Fig. 8. Unfortunately, our approach heavily relies on not looking at the configuration outside the droplet itself. For that reason we instead need to find for each point in the droplet appropriate lengths of the East-extensions in directions $u_0$ and $u_1$, so as to cover the point without going outside the target droplet (see Fig. 9).

Following Sect. 7.1 we define $N^{\textrm{cr}},N^{\textrm{int}},\ell ^{(n)}$ by Eq. (37). In this section there are no fractional scales, so *n* is an integer. Further let ${\Lambda }^{(0)}$ be as in Sect. 7.1 with radii $\underline{r}^{(0)}$ and side lengths $\underline{s}^{(0)}$. For $n\in [N^{\textrm{int}}]$ set

$$\begin{aligned} s_j^{(n)}={\left\{ \begin{array}{ll}s_j^{(0)}\ell ^{(n)}&{}-k<j\leqslant k\\ s_j^{(0)}&{}k+1<j<3k\end{array}\right. } \end{aligned}$$

and $s_{-k}^{(n)}$ and $s_{k+1}^{(n)}$ as required for $\underline{s}^{(n)}$ to be the side lengths of a droplet. Let $\underline{r}^{(n)}$ be the corresponding radii such that $r_{-k}^{(n)}=r_{-k}^{(0)}$ and $r_{k+1}^{(n)}=r_{k+1}^{(0)}$. Finally, set ${\Lambda }^{(n)}={\Lambda }(\underline{r}^{(n)})$ as usual (see Fig. 8).

Fix $n\in [N^{\textrm{int}}]$. Observe that we can cover ${\Lambda }^{(n+1)}$ with droplets $(D_{{\kappa }})_{{\kappa }\in [K]}$ so that the following conditions all hold (see Fig. 9).

- For all $${\kappa }\in [K]$$, $$D_{{\kappa }}\subset {\Lambda }^{(n+1)}$$;
- $$\bigcup _{{\kappa }=2}^{K-1}D_{\kappa }={\Lambda }^{(n+1)}$$;
- $$K=O(\ell ^{(n+1)}/\ell ^{(n)})$$;
- any segment of length $\ell ^{(n)}/(C{\varepsilon })$ perpendicular to $u_j$ for some $j\in [4k]$ intersects at most *O*(1) of the $D_{{\kappa }}$;
- droplets are assigned a *generation* $g\in \{0,1,2\}$, so that only $D_0={\Lambda }^{(n)}$ is of generation $g=0$, only $D_1={\Lambda }(\underline{r}^{(n)}+l_1\underline{v}_1)$ is of generation $g=1$, where
  $$\begin{aligned} l_1=\frac{r^{(n+1)}_k-r^{(n)}_k}{\langle u_1,u_k\rangle }, \end{aligned}$$
  so that $D_1$ spans the $u_{k+1}$-side of ${\Lambda }^{(n+1)}$;
- if ${\kappa }\geqslant 2$, then $D_{{\kappa }}$ is of generation $g=2$, and is of the form
  $$\begin{aligned} D_{\kappa }=y_{{\kappa }}u_1+{\Lambda }\left( \underline{r}^{(n)}+l_{{\kappa }}\underline{v}_0\right) \end{aligned}$$
  for certain $l_{{\kappa }}\geqslant 0$ and $y_{{\kappa }}\in [0,l_1]$ multiple of ${\lambda }_1$.

To construct the $D_{\kappa }$ of generation 2, it essentially suffices to increment $y_{\kappa }$ by $\Theta (\ell ^{(n)}/{\varepsilon })$ and define $l_{\kappa }$ to be the largest possible, so that $D_{\kappa }\subset {\Lambda }^{(n+1)}$. Finally, we add to our collection of droplets the ones with $y_{\kappa }$ corresponding to a corner of ${\Lambda }^{(n+1)}$ and again take $l_{\kappa }$ maximal (see Fig. 9). Note that one is able to get $K=O(\ell ^{(n+1)}/\ell ^{(n)})$ thanks to the fact that $s^{(n)}_{-k}$ and $s_{k+1}^{(n)}$ are $\Theta (\ell ^{(n)}/{\varepsilon })$. We direct the interested reader to [4, Appendix E] for the explicit details of a similar construction in arbitrary dimension.

#### Definition 8.1

(*n*-*traversability*). Fix $n\in [N^{\textrm{int}}]$ and let $R\subset {\Lambda }^{(n+1)}$ be a region of the form

63 $$\begin{aligned} \bigcup _{I\in \mathcal I}\left( \bigcap _{{\kappa }\in I} D_{\kappa }\setminus \bigcup _{{\kappa }\in [K]\setminus I} D_{\kappa }\right) \end{aligned}$$

for some family $\mathcal I$ of subsets of [*K*]. We say that *R* is *n*-*traversable* ($\mathcal T_n(R)$ occurs^7^) if for all $j\in (-k,k)$ and every segment $S\subset R$ perpendicular to $u_j$ of length at least ${\delta }\ell ^{(n)}/{\varepsilon }$ the following two conditions hold.

- If *S* is at distance at least *W* from the boundary of all $D_{{\kappa }}$, then the event $\mathcal H(S)$ occurs.
- If *S* is at distance at most *W* from a side of a $D_{{\kappa }}$ parallel to *S* for some ${\kappa }\in [K]$, but *S* does not intersect any non-parallel side of any $D_{{\kappa }'}$, then the event $\mathcal H^W(S)$ occurs.

Roughly speaking, *R* must be one of the polygonal pieces into which the boundaries of all $D_{\kappa }$ cut ${\Lambda }^{(n+1)}$. It is *n*-traversable, if segments of the size slightly smaller than ${\Lambda }^{(n)}$ contain helping sets for the directions in $(-k,k)$. However, we only require this slightly away from the boundaries of $D_{\kappa }$ and instead add *W*-helping sets close to boundaries, so that we can still cross them but keep the following independence.

#### Remark 8.2

Note that *n*-traversability events are product over the disjoint regions into which all the boundaries of $(D_{\kappa })_{{\kappa }\in [K]}$ partition ${\Lambda }^{(n+1)}$.

#### Definition 8.3

(*Two-dimensional East-extension*). For $n\in [N^{\textrm{int}}]$ we say that we *East-extend* ${\Lambda }^{(n)}$ to ${\Lambda }^{(n+1)}$ if $\mathcal S\mathcal G^\textbf{1}(D_1)$ is defined by East-extending ${\Lambda }^{(n)}$ by $l_1$ in direction $u_1$ and $\mathcal S\mathcal G^\textbf{1}({\Lambda }^{(n+1)})=\mathcal S\mathcal G^\textbf{1}(D_1)\cap \mathcal T_n({\Lambda }^{(n+1)}{\setminus } D_1)$.

Indeed, Definition 8.1 gives $\mathcal T_n({\Lambda }^{(n+1)}\setminus D_1)$, since Eq. (63) is satisfied:

$$\begin{aligned} {\Lambda }^{(n+1)}\setminus D_1=\bigcup _{{\kappa }\in [K]}D_{\kappa }\setminus D_1=\bigcup _{I\subset [K]\setminus \{0,1\}}\left( \bigcap _{{\kappa }\in I}D_{\kappa }\setminus \bigcup _{{\kappa }\not \in I}D_{\kappa }\right) . \end{aligned}$$

Armed with this notion, we are ready to define our SG events up to the internal scale for our models of interest.

#### Definition 8.4

(*Balanced rooted internal SG*). Let $\mathcal U$ be balanced rooted. We say that ${\Lambda }^{(0)}$ is SG ($\mathcal S\mathcal G^\textbf{1}({\Lambda }^{(0)}$ occurs), if all sites in ${\Lambda }^{(0)}$ are infected. We then recursively define $\mathcal S\mathcal G^\textbf{1}({\Lambda }^{(n+1)})$ for $n\in [N^{\textrm{int}}]$ by East-extending ${\Lambda }^{(n)}$ to ${\Lambda }^{(n+1)}$ (see Definition 8.3).

We are now ready to state our bound on the probability of $\mathcal S\mathcal G^\textbf{1}({\Lambda }^{(N^{\textrm{int}})})$ and ${\gamma }({\Lambda }^{(N^{\textrm{int}})})$ (recall Sect. 3.6).

#### Theorem 8.5

Let $\mathcal U$ be balanced rooted (classes (b) and (e)). Then

$$\begin{aligned} {\gamma }\left( {\Lambda }^{(N^{\textrm{int}})}\right)&{}\leqslant \exp \left( \frac{\log (1/q)\log \log \log (1/q)}{{\varepsilon }^3q^{\alpha }}\right) ,\\ {\mu }\left( \mathcal S\mathcal G^\textbf{1}\left( {\Lambda }^{(N^{\textrm{int}})}\right) \right)&{}\geqslant \exp \left( \frac{-1}{{\varepsilon }^2q^{\alpha }}\right) . \end{aligned}$$

The rest of Sect. 8.1 is dedicated to the proof of Theorem 8.5. As usual, the probability bound is not hard (see Lemma 8.7 below), while the relaxation time is bounded recursively. However, we need to obtain such a recursive relation, using Proposition 4.6 twice (see Lemma 8.6 below). Yet, thanks to the additional $\log (1/q)$ factor as compared to Theorem 7.3 (and the $\log \log \log (1/q)$ one, see Remark 1.6), the computations need not be as precise and, in particular, do not rely on Lemma 4.11.

Note that ${\gamma }({\Lambda }^{(0)})=1$, since Eq. (15) is trivial, as $\mathcal S\mathcal G^\textbf{1}({\Lambda }^{(0)})$ is a singleton. For $m\geqslant 1$ and $n\in [N^{\textrm{int}}]$ denote

64 $$\begin{aligned} a^{(n)}_{m}=\max _{j\in \{0,1\}}{\mu }^{-1}\left( \left. \mathcal S\mathcal G^\textbf{1}\left( {\Lambda }^{(n)}+\left( \lfloor (3/2)^{m+1}\rfloor -\lfloor (3/2)^{m}\rfloor \right) {\lambda }_j u_j\right) \right| \mathcal S\mathcal G^\textbf{1}\left( {\Lambda }^{(n)}\right) \right) .\nonumber \\ \end{aligned}$$

For the sake of simplifying expressions we abusively assume that for all ${\kappa }\in [K]$ the length $l_{\kappa }$ is of the form ${\lambda }_0\lfloor (3/2)^m\rfloor$ with integer *m*. Without this assumption, one would need to treat the term corresponding to $m=M-1$ in Proposition 4.6 separately, but identically. We next deduce Theorem 8.5 from the following two lemmas.

#### Lemma 8.6

For $n<N^{\textrm{int}}$ we have

$$\begin{aligned} {\gamma }\left( {\Lambda }^{(n+1)}\right) \leqslant \frac{{\gamma }({\Lambda }^{(n)})e^{O(C^2)\log ^2(1/q)}}{({\mu }(\mathcal S\mathcal G^\textbf{1}({\Lambda }^{(n+1)})){\mu }(\mathcal T_n({\Lambda }^{(n+1)})))^{O(1)}}\prod _{m=1}^{M^{(n)}} a_m^{(n)},\end{aligned}$$

where .

#### Lemma 8.7

For any $n\leqslant N^{\textrm{int}}$ and $m\geqslant 1$ we have

65 $$\begin{aligned} a_m^{(n)}\leqslant {}&{\mu }^{-1}\left( \mathcal S\mathcal G^\textbf{1}\left( {\Lambda }^{(n)}\right) \right) \leqslant {\mu }^{-1}\left( \mathcal S\mathcal G^\textbf{1}\left( {\Lambda }^{(n)}\right) \right) {\mu }^{-1}\left( \mathcal T_{n-1}\left( {\Lambda }^{(n)}\right) \right) \nonumber \\ \leqslant {}&\min \left( \left( {\delta }q^{\alpha }W^n\right) ^{-W^n/{\varepsilon }^2},e^{1/({\varepsilon }^2q^{\alpha })}\right) . \end{aligned}$$

From Lemmas 8.6 and 8.7 and the explicit expressions Eq. (37), we get

$$\begin{aligned} {\gamma }\left( {\Lambda }^{(N^{\textrm{int}})}\right)&{}\leqslant e^{\log ^{O(1)}(1/q)}\prod _{n=0}^{N^{\textrm{int}}-1}\left( {\mu }\left( \mathcal S\mathcal G^\textbf{1}\left( {\Lambda }^{(n+1)}\right) \right) {\mu }\left( \mathcal T_n\left( {\Lambda }^{(n+1)}\right) \right) \right) ^{-O(1)}\prod _{m=1}^{M^{(n)}}a_m^{(n)}\\&{}\leqslant e^{\log ^{O(1)}(1/q)}\prod _{n=0}^{N^{\textrm{int}}-1}\left( {\mu }\left( \mathcal S\mathcal G^\textbf{1}\left( {\Lambda }^{(n+1)}\right) \right) {\mu }\left( \mathcal T_n\left( {\Lambda }^{(n+1)}\right) \right) \right) ^{-O(\log (1/q))}\\&{}\leqslant \exp \left( \frac{\log (1/q)\log \log \log (1/q)}{{\varepsilon }^3q^{\alpha }}\right) . \end{aligned}$$

Since the second inequality in Theorem 8.5 is contained in Lemma 8.7, this concludes the proof of the theorem modulo Lemmas 8.6 and 8.7.

#### Proof of Lemma 8.6

Let us start by recalling a general fact about product measures. Consider two disjoint regions $A,B\subset {\mathbb Z} ^2$ and a product measure ${\nu }$ on ${\Omega }_{A}\times {\Omega }_B$. The law of total variance and convexity give

66 $$\begin{aligned} {\text {Var}}_{{\nu }_{A\cup B}}(f)={\nu }_{B}\left( {\text {Var}}_{{\nu }_A}(f)\right) +{\text {Var}}_{{\nu }_{B}}\left( {\nu }_{A}(f)\right) \leqslant {\nu }({\text {Var}}_{{\nu }_A}(f)+{\text {Var}}_{{\nu }_B}(f)). \end{aligned}$$

Fix $n\in [N^{\textrm{int}}]$. Applying Eq. (66) several times (in view of Remark 8.2 and Definition 8.3), we obtain

67 $$\begin{aligned}&{\text {Var}}_{{\Lambda }^{(n+1)}}\left( f|\mathcal S\mathcal G^\textbf{1}\left( {\Lambda }^{(n+1)}\right) \right) \nonumber \\&{}\leqslant {\mu }_{{\Lambda }^{(n+1)}}\left( \left. {\text {Var}}_{D_1}\left( f|\mathcal S\mathcal G^\textbf{1}(D_1)\right) +\sum _{{\kappa }=2}^{K-1}{\text {Var}}_{R_{\kappa }}\left( f|\mathcal T_n\left( R_{\kappa }\right) \right) \right| \mathcal S\mathcal G^\textbf{1}\left( {\Lambda }^{(n+1)}\right) \right) \nonumber \\&{}\leqslant \sum _{{\kappa }=1}^{K-1}{\mu }_{{\Lambda }^{(n+1)}}\left( \left. {\text {Var}}_{D_{\kappa }\cup D_1}\left( f|\mathcal S\mathcal G^\textbf{1}(D_1),\mathcal T_n(D_{\kappa }\setminus D_1)\right) \right| \mathcal S\mathcal G^\textbf{1}\left( {\Lambda }^{(n+1)}\right) \right) , \end{aligned}$$

where $R_{\kappa }=D_{\kappa }\setminus \bigcup _{{\kappa }'=1}^{{\kappa }-1}D_{{\kappa }'}$. Since the terms above are treated identically (except ${\kappa }=1$, which is actually simpler), without loss of generality we focus on ${\kappa }=2$.

Recall from Definition 8.3 that $\mathcal S\mathcal G^\textbf{1}(D_1)$ was defined by East-extending $D_0$ in direction $u_1$. Further East-extend $D_0$ by $l_2$ (recall that $D_2=y_2u_1+{\Lambda }(\underline{r}^{(n)}+l_2\underline{v}_0)$) in direction $u_0$, so that $\mathcal S\mathcal G^\textbf{1}(D_2)$ is also defined. Let $V=D_1\cup D_2$ (that is a $\dashv$ shaped region in Fig. 9) and

68 $$\begin{aligned} \mathcal S\mathcal G^\textbf{1}(V)=\mathcal S\mathcal G^\textbf{1}(D_1)\cap \mathcal T_n(D_2\setminus D_1). \end{aligned}$$

Using a two-block dynamics (see e.g. Lemma A.1), we have

69 $$\begin{aligned}{} & {} {\text {Var}}_{V}(f|\mathcal S\mathcal G^\textbf{1}(V))\nonumber \\{} & {} \quad \leqslant \frac{{\mu }_{V}({\text {Var}}_{D_1}(f|\mathcal S\mathcal G^\textbf{1}(D_1))+{\mathbb {1}} _{\mathcal E}{\text {Var}}_{V\setminus D_1}(f|\mathcal T_n(V\setminus D_1))|\mathcal S\mathcal G^\textbf{1}(V))}{{\Omega }({\mu }(\mathcal E|\mathcal S\mathcal G^\textbf{1}(V)))}, \end{aligned}$$

where

70 $$\begin{aligned} \mathcal E=\mathcal S\mathcal G^\textbf{1}\left( {\Lambda }^{(n)}+y_2u_1\right) \cap \mathcal T_n\left( D_1\cap D_2\right) \subset {\Omega }_{D_1}. \end{aligned}$$

Recalling Definitions 4.1, 8.1 and 8.3, Eq. (70) and the fact that each segment of length $\ell ^{(n)}/({\varepsilon }C)\gg {\delta }\ell ^{(n)}/{\varepsilon }$ intersects at most *O*(1) droplets, we see that

71 $$\begin{aligned} \mathcal E\cap \mathcal T_n(V\setminus D_1)&{}\subset \mathcal S\mathcal G^\textbf{1}\left( {\Lambda }^{(n)}+y_2u_1\right) \cap \mathcal T^\textbf{1}\left( D_2\setminus \left( {\Lambda }^{(n)}+y_2u_1\right) \right) \nonumber \\&{}= \mathcal S\mathcal G^\textbf{1}(D_2). \end{aligned}$$

By Eq. (71) and convexity of the variance, we obtain

72 $$\begin{aligned}{} & {} {\mu }_{V}\left( \left. {\mathbb {1}} _{\mathcal E}{\text {Var}}_{V\setminus D_1}(f|\mathcal T_n(V\setminus D_1))\right| \mathcal S\mathcal G^\textbf{1}(V)\right) \nonumber \\{} & {} \quad {}\leqslant \frac{{\mu }(\mathcal E)}{{\mu }(\mathcal S\mathcal G^\textbf{1}(V))}{\mu }_{V}\left( {\text {Var}}_{D_2}\left( f|\mathcal E\cap \mathcal T_n\left( V\setminus D_1\right) \right) \right) \nonumber \\{} & {} \quad {}\leqslant \frac{{\mu }(\mathcal E){\mu }(\mathcal S\mathcal G^\textbf{1}(D_2)){\mu }_{V}({\text {Var}}_{D_2}(f|\mathcal S\mathcal G^\textbf{1}(D_2)))}{{\mu }(\mathcal S\mathcal G^\textbf{1}(V)){\mu }(\mathcal E\cap \mathcal T_n(V\setminus D_1))}\nonumber \\{} & {} \quad {}\leqslant \frac{{\mu }_{V}({\text {Var}}_{D_2}(f|\mathcal S\mathcal G^\textbf{1}(D_2)))}{{\mu }^2(\mathcal T_n({\Lambda }^{(n+1)}))}. \end{aligned}$$

Indeed, in the last line we recalled the definitions of $\mathcal S\mathcal G^\textbf{1}(D_2)$, $\mathcal S\mathcal G^\textbf{1}(V)$ and $\mathcal E$ (see Definition 4.4 and Eqs. (68) and (70)), while in the second one we took into account that for any events $\mathcal A\subset \mathcal B$ with ${\mu }(\mathcal A)>0$ it holds that

73 $$\begin{aligned} {\text {Var}}(f|\mathcal A)=\min _{c\in {\mathbb R} }{\mu }\left( \left. (f-c)^2\right| \mathcal A\right) \leqslant \frac{{\mu }((f-{\mu }(f|\mathcal B))^2{\mathbb {1}} _{\mathcal A})}{{\mu }(\mathcal A)}\leqslant \frac{{\mu }(\mathcal B)}{{\mu }(\mathcal A)}{\text {Var}}(f|\mathcal B)\nonumber \\ \end{aligned}$$

and Eq. (71).

We plug Eq. (72) in Eq. (69) and note that by the Harris inequality, Eqs. (7), (8), ${\mu }(\mathcal E|\mathcal S\mathcal G^\textbf{1}(V))\geqslant {\mu }(\mathcal E)\geqslant {\mu }(\mathcal S\mathcal G^\textbf{1}({\Lambda }^{(n)})){\mu }(\mathcal T_n({\Lambda }^{(n+1)}))$. This yields

74 $$\begin{aligned} {\text {Var}}_{V}\left( f|\mathcal S\mathcal G^\textbf{1}(V)\right)&{}\leqslant \frac{O(1){\mu }_{V}({\text {Var}}_{D_1}(f|\mathcal S\mathcal G^\textbf{1}(D_1))+{\text {Var}}_{D_2}(f|\mathcal S\mathcal G^\textbf{1}(D_2)))}{{\mu }(\mathcal S\mathcal G^\textbf{1}({\Lambda }^{(n)})){\mu }(\mathcal S\mathcal G^\textbf{1}(V)){\mu }^3(\mathcal T_n({\Lambda }^{(n+1)}))}\nonumber \\&{}\leqslant \frac{O(1){\mu }_{V}({\text {Var}}_{D_1}(f|\mathcal S\mathcal G^\textbf{1}(D_1))+{\text {Var}}_{D_2}(f|\mathcal S\mathcal G^\textbf{1}(D_2)))}{{\mu }^2(\mathcal S\mathcal G^\textbf{1}({\Lambda }^{(n+1)})){\mu }^3(\mathcal T_n({\Lambda }^{(n+1)}))} \end{aligned}$$

where the second inequality uses Eq. (68) and Definition 8.3.

As in Eqs. (40) and (41), Proposition 4.6 gives

75 $$\begin{aligned}{} & {} {\gamma }(D_2)\leqslant \max \left( {\gamma }\left( {\Lambda }^{(n)}\right) ,{\mu }^{-1}\left( \mathcal S\mathcal G^\textbf{1}\left( {\Lambda }^{(n)}\right) \right) \right) e^{O(C^2)\log ^2(1/q)}q^{-O(WM)}\nonumber \\{} & {} \quad \times \frac{{\mu }(\mathcal S\mathcal G^\textbf{1}({\Lambda }^{(n)}))}{{\mu }(\mathcal S\mathcal G^\textbf{1}(D_2))}\prod _{m=1}^{M}a_m^{(n)} \end{aligned}$$

with $M=\min \{m:{\lambda }_0(3/2)^{m+1}\geqslant l_2\}\leqslant M^{(n)}$. Plugging Eqs. (15) and (75) (and their analogues for $D_1$) into Eq. (74), we obtain

$$\begin{aligned} {\gamma }(V)&{}\leqslant \frac{{\gamma }({\Lambda }^{(n)})e^{O(C^2)\log ^2(1/q)}\prod _{m=1}^{M^{(n)}}a_m^{(n)}}{{\mu }^3(\mathcal S\mathcal G^\textbf{1}({\Lambda }^{(n+1)})){\mu }^3(\mathcal T_n({\Lambda }^{(n+1)}))\min _{\kappa }{\mu }(\mathcal S\mathcal G^\textbf{1}(D_{\kappa }))}\\&{}\leqslant \frac{{\gamma }\left( {\Lambda }^{(n)}\right) e^{O(C^2)\log ^2(1/q)}\prod _{m=1}^{M^{(n)}}a_m^{(n)}}{{\mu }^4\left( \mathcal S\mathcal G^\textbf{1}\left( {\Lambda }^{(n+1)}\right) \right) {\mu }^4(\mathcal T_n({\Lambda }^{(n+1)}))}, \end{aligned}$$

where the last inequality uses Eq. (71) and that $\mathcal S\mathcal G^\textbf{1}(D_1)\supset \mathcal S\mathcal G^\textbf{1}({\Lambda }^{(n+1)})$ by Definition 8.3. Plugging this into Eq. (67), concludes the proof of Lemma 8.6, since $K=O(\ell ^{(n+1)}/\ell ^{(n)})\leqslant O(\log ^4(1/q))$, as noted in Remark 7.1. $\square$

#### Proof of Lemma 8.7

The first inequality in Eq. (65) follows from the Harris inequality Eq. (8), while the second one is trivial. Therefore, we turn to the last one and fix $n\in [N^{\textrm{int}}]$. Note that by Definitions 4.4, 8.1 and 8.3

76 $$\begin{aligned} {\mu }\left( \mathcal S\mathcal G^\textbf{1}\left( {\Lambda }^{(n+1)}\right) \right) \geqslant {\mu }\left( \mathcal S\mathcal G^\textbf{1}\left( {\Lambda }^{(n)}\right) \right) {\mu }\left( \mathcal T_n\left( {\Lambda }^{(n+1)}\right) \right) {\mu }\left( \mathcal T^\textbf{1}\left( D_1\setminus D_0\right) \right) . \end{aligned}$$

We therefore proceed by induction starting with

77 $$\begin{aligned} {\mu }\left( \mathcal S\mathcal G^\textbf{1}\left( {\Lambda }^{(0)}\right) \right) =q^{|{\Lambda }^{(0)}|}=q^{\Theta (1/{\varepsilon }^2)}. \end{aligned}$$

We observe that from Definition 8.1, in order to ensure the occurrence of $\mathcal T_n({\Lambda }^{(n+1)})$, it suffices to have $O(WK\ell ^{(n+1)})/(\ell ^{(n)}{\delta })$ well-placed *W*-helping sets and $O((\ell ^{(n+1)})^2)/(\ell ^{(n)}{\delta }{\varepsilon })$ helping sets for segments of length ${\delta }\ell ^{(n)}/(3{\varepsilon })$. Indeed, we may split lines perpendicular to each $u_j$ for $j\in (-k,k)$ into successive disjoint segments of length ${\delta }\ell ^{(n)}/(3{\varepsilon })$ with a possible smaller leftover. It is then sufficient to place *W*-helping sets or helping sets depending on whether the segment under consideration is close to a parallel boundary of one of the $D_{\kappa }$ or not. Note that here we crucially use the assumption that each segment of length $\ell ^{(n)}/(C{\varepsilon })\gg {\delta }\ell ^{(n)}/{\varepsilon }$ intersects only *O*(1) droplets.

Recall that $1/{\varepsilon }\gg 1/{\delta }\gg W\gg 1$, $\ell ^{(N^{\textrm{cr}})}=W^{O(1)}q^{\alpha }$, $K=O(\ell ^{(n+1)}/\ell ^{(n)})\leqslant \log ^{O(1)}(1/q)$, the explicit expressions Eq. (37) and Observation 3.11. Then the Harris inequality Eq. (7), yields

78 $$\begin{aligned}{} & {} {\mu }\left( \mathcal T_n\left( {\Lambda }^{(n+1)}\right) \right) \nonumber \\{} & {} \quad \geqslant {} q^{O(W^2K\ell ^{(n+1)})/(\ell ^{(n)}{\delta })}\left( 1-e^{-q^{\alpha }{\delta }\ell ^{(n)}/O({\varepsilon })}\right) ^{O((\ell ^{(n+1)})^2/(\ell ^{(n)}{\delta }{\varepsilon }))}\nonumber \\{} & {} \quad \geqslant {}e^{-\log ^{O(1)}(1/q)}\times {\left\{ \begin{array}{ll} \left( {\delta }q^{\alpha }W^n\right) ^{W^n/({\delta }^2{\varepsilon })}&{}n\leqslant N^{\textrm{cr}}\\ \exp \left( -1/\left( q^{{\alpha }}\exp \left( W^{\exp (n-N^{\textrm{cr}})}\right) \right) \right) &{}n> N^{\textrm{cr}}. \end{array}\right. } \end{aligned}$$

Essentially the same computation leads to the same bound for ${\mu }(\mathcal T^\textbf{1}(D_1\setminus D_0))$ (see Eq. (56)). The only difference is that only *O*(1) *W*-helping sets and $O(\ell ^{(n+1)}/{\varepsilon })$ helping sets are needed. Further recalling Eqs. (76) and (77), it is not hard to check Eq. (65). $\square$

### FA-1f global dynamics

We next import the global FA-1f dynamics together with much of the mesoscopic multi-directional East one simultaneously from [22].

#### Proposition 8.8

Let $\mathcal U$ have a finite number of stable directions, $T=\exp (\log ^4(1/q)/q^{\alpha })$ and $\underline{r}^{\textrm{int}}$ be such that the associated side lengths satisfy $C\leqslant s^{\textrm{int}}_j\leqslant O(\ell ^{\textrm{int}})$ for all $j\in [4k]$. Assume that for all $l\in [0,\ell ^{\textrm{mes}}]$ multiple of ${\lambda }_0$ the event $\mathcal S\mathcal G^\textbf{1}({\Lambda }(\underline{r}^{\textrm{int}}+l\underline{v}_0))$ is nonempty, decreasing, translation invariant and satisfies

$$\begin{aligned} \left( 1-{\mu }\left( \mathcal S\mathcal G^\textbf{1}\left( {\Lambda }\left( \underline{r}^{\textrm{int}}+l\underline{v}_0\right) \right) \right) \right) ^T T^W=o(1). \end{aligned}$$

Then,

$$\begin{aligned} {\mathbb E} _{{\mu }}[{\tau }_0]\leqslant \frac{\max _{l\in [0,\ell ^{\textrm{mes}}]}{\gamma }({\Lambda }(\underline{r}^{\textrm{int}}+l\underline{v}_0))}{(q^{1/{\delta }}\min _{l\in [0,\ell ^{\textrm{mes}}]}{\mu }(\mathcal S\mathcal G^\textbf{1}({\Lambda }(\underline{r}^{\textrm{int}}+l\underline{v}_0))))^{\log (1/q)/{\delta }}}. \end{aligned}$$

The proof is as in [22], up to the following minor modifications. Firstly, one needs to replace the base of the snail by , which has a similar shape by hypothesis. Secondly, the event that the base is super good on [22] should be replaced by $\mathcal S\mathcal G^\textbf{1}({\Lambda }^{\textrm{mes}})$. Finally, [22, Proposition 4.9] is substituted by the definition Eq. (15) of ${\gamma }({\Lambda }^{\textrm{mes}})$. As Proposition 8.8 is essentially the entire content of [22] (see particularly Proposition 4.12 and Remark 4.8 there), we refer the reader to that work for the details.

#### Proof of Theorem 1(e)

Let $\mathcal U$ be balanced rooted with finite number of stable directions. Recall ${\Lambda }^{(N^{\textrm{int}})}={\Lambda }(\underline{r}^{(N^{\textrm{int}})})$ with $\underline{r}^{(N^{\textrm{int}})}=:\underline{r}^{\textrm{int}}$ from Sect. 7.1 if $k=1$ and from Sect. 8.1 if $k\geqslant 2$. Fix $l\in [0,\ell ^{\textrm{mes}}]$ multiple of ${\lambda }_0$ and East-extend ${\Lambda }^{(N^{\textrm{int}})}$ by *l* in direction $u_0$. It is not hard to check from Definition 4.4 and Observation 3.11 that

$$\begin{aligned} \frac{{\mu }(\mathcal S\mathcal G^\textbf{1}({\Lambda }(\underline{r}^{\textrm{int}}+l\underline{v}_0)))}{{\mu }(\mathcal S\mathcal G^\textbf{1}({\Lambda }(\underline{r}^{\textrm{int}})))}={\mu }\left( \mathcal T^\textbf{1}\left( T\left( \underline{r}^{\textrm{int}},l,0\right) \right) \right) =q^{O(W)} \end{aligned}$$

(see Eq. (36)). Then, by Proposition 4.6, Theorems 7.3 and 8.5 and the Harris inequality Eq. (7), we obtain

$$\begin{aligned} {\mu }\left( \mathcal S\mathcal G^\textbf{1}\left( {\Lambda }\left( \underline{r}^{\textrm{int}}+l\underline{v}_0\right) \right) \right)&{}\geqslant \exp \left( \frac{-2}{{\varepsilon }^2q^{\alpha }}\right) \\ {\gamma }\left( {\Lambda }\left( \underline{r}^{\textrm{int}}+l\underline{v}_0\right) \right)&{}\leqslant {\left\{ \begin{array}{ll} \exp \left( \frac{\log (1/q)}{{\varepsilon }^3q^{\alpha }}\right) &{}k=1,\\ \exp \left( \frac{2\log (1/q)\log \log \log (1/q)}{{\varepsilon }^3q^{\alpha }}\right) &{}k\geqslant 2.\end{array}\right. } \end{aligned}$$

Plugging this in Proposition 8.8, we obtain

79 $$\begin{aligned} {\mathbb E} _{{\mu }}[{\tau }_0]\leqslant {\left\{ \begin{array}{ll} \exp \left( \frac{2\log (1/q)}{{\varepsilon }^3q^{\alpha }}\right) &{}k=1,\\ \exp \left( \frac{3\log (1/q)\log \log \log (1/q)}{{\varepsilon }^3q^{\alpha }}\right) &{}k\geqslant 2,\end{array}\right. } \end{aligned}$$

which concludes the proof of Theorem 1(e) in the case $k=1$ and of Eq. (4) for $k\geqslant 2$. The full result of Theorem 1(e) for $k\geqslant 2$ is proved identically, replacing Theorem 8.5 by the stronger Theorem C.1. $\square$

## Balanced Models with Infinite Number of Stable Directions

We finally turn to balanced models with infinite number of stable directions (class (b)). The internal dynamics was already handled in Sect. 8.1. The mesoscopic one (Sect. 9.1) is essentially the same as the the internal one, using two-dimensional East-extensions. The global dynamics (Sect. 9.2) also uses an East mechanism analogous to the FA-1f one from [22] used in Sect. 8.2.

### East mesoscopic dynamics

Given that the bound we are aiming for in Theorem 1(b) is much larger than those in previous sections, there is a lot of margin and our reasoning is far from tight for the sake of simplicity.

Recall $N^{\textrm{int}}$ and $\ell ^{(n)}$ for $n\leqslant N^{\textrm{int}}$ from Eq. (37), the droplets ${\Lambda }^{(n)}$ from Sect. 8.1, their SG events from Definition 8.4. For $n>N^{\textrm{int}}$, we set $\ell ^{(n)}=W^{n-N^{\textrm{int}}}\ell ^{(N^{\textrm{int}})}$ and define $\underline{s}^{(n)},\underline{r}^{(n)},{\Lambda }^{(n)}$ as in Sect. 8.1. Recall Sect. 3.4. Further let $N^{\textrm{mes}}=\textrm{inf}\{n:\ell ^{(n)}/{\varepsilon }\geqslant \ell ^{\textrm{mes}}=q^{-C}\}=\Theta (C\log (1/q)/\log W)$ and assume for simplicity that $\ell ^{(N^{\textrm{mes}})}=q^{-C}{\varepsilon }$. We are only be interested in $n\leqslant N^{\textrm{mes}}$ and extend Definitions 8.1, 8.3 and 8.4 to such *n* without change. With these conventions, our goal is the following.

#### Theorem 9.1

Let $\mathcal U$ be a balanced model with infinitely many stable directions (class (b)). Then

$$\begin{aligned} {\gamma }\left( {\Lambda }^{(N^{\textrm{mes}})}\right)&{}\leqslant \exp \left( \frac{\log ^{2}(1/q)}{{\varepsilon }^3q^{\alpha }}\right) ,&{\mu }\left( \mathcal S\mathcal G^\textbf{1}\left( {\Lambda }^{(N^{\textrm{mes}})}\right) \right)&{}\geqslant \exp \left( \frac{-2}{{\varepsilon }^2q^{\alpha }}\right) . \end{aligned}$$

The rest of Sect. 9.1 is dedicated to the proof of Theorem 9.1. The proof is essentially identical to the one of Theorem 8.5, so we only indicate the necessary changes. To start with, Lemma 8.6 applies without change for $n\in [N^{\textrm{int}},N^{\textrm{mes}})$. Also, the Harris inequality Eq. (8) still implies that $a_m^{(n)}\leqslant {\mu }^{-1}(\mathcal S\mathcal G^\textbf{1}({\Lambda }^{(n)}))\leqslant {\mu }^{-1}(\mathcal S\mathcal G^\textbf{1}({\Lambda }^{(N^{\textrm{mes}})}))$. Therefore,

$$\begin{aligned} {\gamma }\left( {\Lambda }^{(N^m)}\right) \leqslant \frac{{\gamma }({\Lambda }^{(N^{\textrm{int}})})e^{\log ^{O(1)}(1/q)}}{({\mu }(\mathcal S\mathcal G^\textbf{1}({\Lambda }^{(N^{\textrm{mes}})}))\min _{n\in [N^{\textrm{mes}}]}{\mu }(\mathcal T_n({\Lambda }^{n+1})))^{O(N^{\textrm{mes}}M^{(N^{\textrm{mes}}-1)})}}. \end{aligned}$$

Recalling the bound on ${\gamma }({\Lambda }^{(N^{\textrm{int}})})$ established in Theorem 8.5, together with the fact that $N^{\textrm{mes}}\leqslant C\log (1/q)$ and $M^{(N^{\textrm{mes}}-1)}\leqslant O(C\log (1/q))$, it suffices to prove that

80 $$\begin{aligned} {\mu }\left( \mathcal S\mathcal G^\textbf{1}\left( {\Lambda }^{(N^{\textrm{mes}})}\right) \right) \min _{n\in [N^{\textrm{mes}}]}{\mu }\left( \mathcal T_n\left( {\Lambda }^{n+1}\right) \right) \geqslant \exp \left( -2/\left( {\varepsilon }^2q^{\alpha }\right) \right) , \end{aligned}$$

in order to conclude the proof of Theorem 9.1.

Once again, the proof of Eq. (80) proceeds similarly to the one of Eq. (65) in Lemma 8.7. Indeed, the same computation as Eq. (78) in the present setting gives that for $n\in [N^{\textrm{int}}, N^{\textrm{mes}})$ we have

81 $$\begin{aligned} {\mu }\left( \mathcal T_n\left( {\Lambda }^{(n+1)}\right) \right) \geqslant q^{O(W^3/{\delta })}\exp \left( -e^{-q^{\alpha }{\delta }\ell ^{(n)}/O({\varepsilon })}O\left( W^2\ell ^{(n)}/({\delta }{\varepsilon })\right) \right) \end{aligned}$$

and similarly for ${\mu }(\mathcal T^\textbf{1}(D_1\setminus D_0))$ (as in the proof of Lemma 8.7, also see Eq. (56)). From Eq. (76) it follows that

$$\begin{aligned}{} & {} {\mu }\left( \mathcal S\mathcal G^\textbf{1}\left( {\Lambda }^{(N^{\textrm{mes}})}\right) \right) \geqslant {\mu }\left( \mathcal S\mathcal G^\textbf{1}\left( {\Lambda }^{(N^{\textrm{int}})}\right) \right) \\{} & {} \quad \times \prod _{n=N^{\textrm{int}}}^{N^{\textrm{mes}}-1}{\mu }\left( \mathcal T^\textbf{1}(D_1\setminus D_0)\right) {\mu }\left( \mathcal T_n\left( {\Lambda }^{(n+1)}\setminus {\Lambda }^{(n)}\right) \right) . \end{aligned}$$

Plugging Eqs. (65), (81) in the r.h.s., this yields Eq. (80) as desired.

### East global dynamics

For the global dynamics we use a simpler version of the procedure of [22, Sect. 5] with East dynamics instead of FA-1f.

**Fig. 10:**
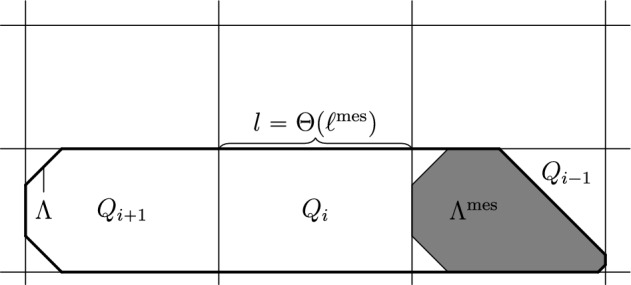
Illustration of the East global dynamics (Sect. 9.2). The shaded droplet ${\Lambda }^{\textrm{mes}}$ inscribed in the box *Q* is extended by 2*l* to the thickened one ${\Lambda }$

#### Proof of Theorem 1(b)

Let $\mathcal U$ be balanced with infinite number of stable directions and recall Sect. 9.1. Set $T=\exp (1/q^{3{\alpha }})$, $\underline{s}^{\textrm{mes}}=\underline{s}^{(N^{\textrm{mes}})}$, $\underline{r}^{\textrm{mes}}=\underline{r}^{(N^{\textrm{mes}})}$ and ${\Lambda }^{\textrm{mes}}={\Lambda }^{(N^{\textrm{mes}})}$. In particular, $s^{\textrm{mes}}_j=\Theta (\ell ^{\textrm{mes}})$ for $j\in [-k, k+1]$ and $s^{\textrm{mes}}_j=\Theta (1/{\varepsilon })$ for $j\in [k+2,3k-1]$. We East-extend ${\Lambda }^{\textrm{mes}}$ by $2l=2({\lambda }_0+r^{\textrm{mes}}_0+r^{\textrm{mes}}_{2k})$ in direction $u_0$ to obtain ${\Lambda }={\Lambda }(\underline{r}^{\textrm{mes}}+2l\underline{v}_0)$. Proposition 4.6,Theorem 9.1 and Definition 4.4, the Harris inequality Eq. (8) and the simple fact that ${\mu }(\mathcal T^\textbf{1}(T(\underline{r}^{\textrm{mes}},2l,0)))=q^{O(W)}$ (by Observation 3.11 and Lemma 4.2 as usual) give

82 $$\begin{aligned} {\gamma }({\Lambda })\leqslant {}&\exp \left( \frac{\log ^2(1/q)}{{\varepsilon }^{O(1)}q^{\alpha }}\right) ,&{\mu }\left( \mathcal S\mathcal G^\textbf{1}({\Lambda })\right) \geqslant {}&\exp \left( \frac{-3}{{\varepsilon }^2q^{\alpha }}\right) . \end{aligned}$$

A similar argument to the rest of the proof was already discussed thoroughly in [22, Sect. 5] and then in [24, Sect. 5], so we only provide a sketch. The adapted approach of [22, Sect. 5] proceeds as follows.

1. Denoting $t_*=\exp (-1/({\varepsilon }^W q^{2{\alpha }}))$, by the main result of [31] it suffices to show that $T{\mathbb P} _{{\mu }}({\tau }_0>t_*)=o(1)$, in order to deduce ${\mathbb E} _{{\mu }}[{\tau }_0]\leqslant t_*+o(1)$.
2. By finite speed of propagation we may work with the $\mathcal U$-KCM on a large discrete torus of size $T\gg t_*$.
3. We partition the torus into strips and the strips into translates of the box $Q={\mathbb H} _{u_0}({\lambda }_0+r^{\textrm{mes}}_0)\cap {\mathbb H} _{u_k}({\rho }_k+r^{\textrm{mes}}_k)\cap {\overline{{\mathbb H} }}_{u_{-k}}(r^{\textrm{mes}}_{-k})\cap {\overline{{\mathbb H} }}_{u_{2k}}(r^{\textrm{mes}}_{2k})$ as shown in Fig. 10. We say *Q* is *good* ($\mathcal G(Q)$ occurs) if for each segment $S\subset Q$ perpendicular to some $u\in {\widehat{\mathcal S}}$ of length ${\varepsilon }\ell ^{\textrm{mes}}$ the event $\mathcal H^W(S)$ occurs. Further define $\mathcal S\mathcal G(Q)$ to occur if the only (integer) translate of ${\Lambda }^{\textrm{mes}}$ contained in *Q* is SG. We say that *the environment is good* ($\mathcal E$ occurs) if all boxes are good and in each strip at least one box is super good. The sizes are chosen so that it is sufficiently likely for this event to always occur up to time $t_*$. Indeed, we have $(1-{\mu }(\mathcal S\mathcal G^\textbf{1}({\Lambda }^{\textrm{mes}})))^TT^W=o(1)$ by Theorem 9.1 and $(1-{\mu }_Q(\mathcal G))T^W=o(1)$ by Observation 3.11.
4. By a standard variational technique it then suffices to prove a Poincaré inequality, bounding the variance of a function conditionally on $\mathcal E$ by the Dirichlet form on the torus. Moreover, since ${\mu }$ and $\mathcal E$ are product w.r.t. the partition of Fig. 10, it suffices to prove this inequality on a single strip.
5. Finally, we prove such a bound, using an auxiliary East dynamics for the boxes and the definition of ${\gamma }$ to reproduce the resampling of the state of a box by moves of the original $\mathcal U$-KCM.

Let us explain the last step above in more detail, as it is the only one that genuinely differs from [22].

Let $Q_i=Q+ilu_0$ and ${\mathbb T} =\bigcup _{i\in [T]}Q_i$ be our strip of interest (indices are considered modulo *T*, since the strip is on the torus). As explained above, our goal is to prove that for all $f:{\Omega }_{\mathbb T} \rightarrow {\mathbb R}$ it holds that

83 $$\begin{aligned} {\text {Var}}_{{\mathbb T} }(f|\mathcal E)\leqslant \exp \left( 1/\left( {\varepsilon }^{O(1)}q^{2{\alpha }}\right) \right) \sum _{x\in {\mathbb T} }{\mu }_{{\mathbb T} }\left( c_x^{{\mathbb T} ,\textbf{1}}{\text {Var}}_x(f)\right) , \end{aligned}$$

where $c_x^{{\mathbb T} ,\textbf{1}}$ takes into account the periodic geometry of ${\mathbb T}$.

By [31, Proposition 3.4] on the generalised East chain we have

84 $$\begin{aligned} {\text {Var}}_{\mathbb T} (f|\mathcal E)\leqslant \exp \left( 1/\left( {\varepsilon }^5q^{2{\alpha }}\right) \right) \sum _{i\in [T]}{\mu }_{{\mathbb T} }\left( \left. {\mathbb {1}} _{\mathcal S\mathcal G(Q_{i-1})}{\text {Var}}_{Q_i}\left( f|\mathcal G\left( Q_i\right) \right) \right| \mathcal E\right) , \end{aligned}$$

since Theorem 9.1 and the Harris inequality Eq. (8) give ${\mu }(\mathcal S\mathcal G(Q)|\mathcal G(Q))\geqslant \exp (-2/({\varepsilon }^2q^{\alpha }))$.^8^

Next observe that ${\Lambda }_i\supset Q_i$, where ${\Lambda }_i={\Lambda }+(i-1)lu_0$ (see Fig. 10). Let $\mathcal G({\Lambda }_i{\setminus } Q_i)\subset \mathcal G(Q_{i+1})\cap \mathcal G(Q_{i-1})$ be the event that $\mathcal H^W(S)$ holds for all segments $S\subset {\Lambda }_i\setminus Q_i$ of length $2{\varepsilon }\ell ^{\textrm{mes}}$ perpendicular to some $u\in {\widehat{\mathcal S}}$. Hence, by convexity of the variance and the fact that ${\mu }(\mathcal E)=1-o(1)$ we have

$$\begin{aligned}{} & {} {\mu }_{\mathbb T} \left( \left. {\mathbb {1}} _{\mathcal S\mathcal G(Q_{i-1})}{\text {Var}}_{Q_i}(f|\mathcal G(Q_i))\right| \mathcal E\right) \\{} & {} \quad \leqslant {}(1+o(1)){\mu }_{\mathbb T} \left( {\text {Var}}_{{\Lambda }_{i}}(f|\mathcal S\mathcal G(Q_{i-1})\cap \mathcal G(Q_i)\cap \mathcal G({\Lambda }_i\setminus Q_i))\right) ,\\{} & {} \quad \leqslant {}(1+o(1)){\mu }_{\mathbb T} \left( {\text {Var}}_{{\Lambda }_i}\left( f|\mathcal S\mathcal G^\textbf{1}({\Lambda }_i)\right) \right) . \end{aligned}$$

Here we used Eq. (73) and $\mathcal S\mathcal G(Q_{i-1})\cap \mathcal G(Q_i)\cap \mathcal G({\Lambda }_i{\setminus } Q_i)\subset \mathcal S\mathcal G^\textbf{1}({\Lambda }_i)$ (recall Definition 4.4) for the second inequality. Finally, recalling Eqs. (15), (82) and (84), we obtain Eq. (83) as desired. $\square$

As already noted, all lower bounds in Theorem 1 are known from [20] and the upper ones for classes (a) and (c) were proved in [22] and [31] respectively. Thus, the proof of Theorem 1 is complete.

## Footnotes

1 We write $f=\Theta (g)$ if there exist $c,C>0$ such that $cg(q)<f(q)<Cg(q)$ for all *q* small enough and use other standard asymptotic notation (see e.g. [20, Sect. 1.2]).

2 See Remark 1.6.

3 Note that in [24, Proposition 4.7] a much larger internal relaxation time of order $\exp (q^{-1/2+o(1)})$ was obtained, but the cost ${\rho }_{\textrm{D}}^{-1}$ of SG droplets was much smaller than the one in the present work, so our treatment here is by no means as sharp for FA-2f as [24].

4 We use a bar to denote “contracted” versions of events (recall Sect. 2.3.1).

5 Recall from Definition 4.1 that $\mathcal S\mathcal T_W$ refers to symmetric traversability with parallelograms in Fig. 2: a shrunken by *W*, but not necessarily requiring *W*-helping sets. Further recall from Sect. 4.1 that for isotropic models $\mathcal T$ and $\mathcal S\mathcal T$ events are the same.

6 Due to the difference between Eq. (15) and [24, Eq. (4.5)], the factor ${\mu }_{{\Lambda }_{i,j}}(\mathcal S\mathcal G({\Lambda }_{i,j}))$ in [24, last display of Sect. 5] cancels out with $\pi (\mathcal S_1)^{-1}$ in [24, Eq. (5.11)] up to a $q^{O(C)}$ factor, rather than compensating the conditioning in [24, last display of Sect. 5], which is absent in our setting.

7 The *n*-traversability $\mathcal T_n$ should not be confused with $({\omega },d)$-traversability $\mathcal T_d^{\omega }$ from Definition 4.1, which only features with $d=0$ and ${\omega }=\textbf{1}$ in the present section.

8 Strictly speaking [31] does not deal with the torus conditioned on having an infection, but this issue is easily dealt with by the method of [6].
